# Perspectives on App-Assisted Self-Testing Using Rapid Diagnostic Tests Among Community Members, Health Care Providers, and Public Health Leaders in Kenya, South Africa, and Zambia: Qualitative Study

**DOI:** 10.2196/70273

**Published:** 2025-11-26

**Authors:** Anjali Sharma, Chanda Mwamba, Patricia Maritim, Hilton Humphries, Zachary Kwena, Derek Pollard, Gabrielle O’Malley, Shawna Cooper, Meagan Bemer, Elizabeth Anne Bukusi, Amber Lauff, Lenika Naiken, Thandanani Madonsela, Jennifer Faith Morton, Dino Rech, Norton Sang, Ayanda Tshazi, Alastair van Heerden, Anna Winters, Paul K Drain

**Affiliations:** 1 Centre for Infectious Disease Research in Zambia Lusaka, Lusaka Zambia; 2 Department of Global Health University of Washington Seattle, WA United States; 3 Department of Health Policy and Management, University of Zambia School of Public Health Lusaka, Lusaka Zambia; 4 Human Sciences Research Council Pietermaritzburg, KwaZulu-Natal South Africa; 5 Division of Social and Behavioural Sciences Faculty of Health Sciences, School of Public Health University of Cape Town Cape Town, Western Cape South Africa; 6 Department of Psychology University of KwaZulu-Natal Pietermaritzburg, KwaZulu-Natal South Africa; 7 Akros Lusaka Zambia; 8 Audere Seattle, WA United States; 9 Kenya Medical Research Institute Nairobi Kenya; 10 Audere Johannesburg South Africa; 11 Vanderbilt University Medical Centre Nashville, TN United States; 12 Akros Missoula, MT United States; 13 School of Medicine University of Washington Seattle, WA United States; 14 Department of Epidemiology University of Washington Seattle, WA United States

**Keywords:** acceptability, home-based testing, mHealth, rapid-diagnostic tests, self-care, self-testing, sub-Saharan Africa

## Abstract

**Background:**

Self-testing using rapid diagnostic tests (RDTs), integrated with mobile phone–based apps, offers potential to improve health outcomes in low-resource settings, including sub-Saharan Africa. Despite its advantages, uptake remains limited because of concerns about accuracy, accessibility, and integration within existing health care systems.

**Objective:**

This study aimed to assess the acceptability of self-testing using RDTs for various health conditions, both independently and app-assisted, among community members, health care providers (HCPs), and community and public health leaders in Kenya, South Africa, and Zambia.

**Methods:**

From May 15 to August 24, 2023, we conducted an exploratory qualitative study across rural and urban areas in the 3 countries. We used key informant interviews with community leaders and public health leaders in the ministries or departments of health, in-depth interviews (IDIs) with HCPs, and IDIs and focus group discussions (FGDs) with community members. Using framework analysis, we categorized data guided by the Theoretical Framework of Acceptability to assess affective attitude, burden, ethicality, intervention coherence, opportunity costs, perceived effectiveness, and self-efficacy toward self-testing using RDTs, with and without assistance from an app, across countries and participant types.

**Results:**

The study involved 178 participants, including 24 key informants, 41 HCPs, and 113 community members who participated in 55 IDIs and 11 FGDs across the 3 countries. Self-testing, both standalone and app-assisted, was highly acceptable to community members, HCPs, and community and public health leaders in the 3 countries for its ability to empower individuals, enhance access to health care, and improve efficiencies in health care delivery. Self-testing was aligned with values of privacy, confidentiality, and autonomy and increased reach to underresourced areas. Participants valued its potential to save time, reduce opportunity costs, and facilitate early diagnosis and treatment, while easing burdens on the health care systems. Participants perceived the benefits of self-testing to outweigh possible risks, including human error, false readings, and emotional distress from unexpected results, especially if linked by an app to real-time guidance and improved health care coordination. Apps were praised for modernity, convenience, and ability to streamline processes for users and health care systems. Foreseeable challenges included resistance from less tech-savvy individuals, ethical concerns such as misuse of self-tests, technological barriers, data security, training needs, and insufficient digital and physical infrastructure for equitable implementation. Regular education, system support, integration, and community trust were identified as critical for success.

**Conclusions:**

Self-testing, both alone and app-assisted, was viewed as acceptable and relevant for increasing health care accessibility and efficiency in these low-resource settings. However, to optimize its benefits, efforts must address challenges related to test accuracy, infrastructure development, data privacy, and integration into existing health care systems. Policies should further emphasize education, support, user-friendly design, and minimal costs to ensure equitable access and effective implementation across diverse populations.

## Introduction

Self-testing integrated with mobile phone–based interventions has the potential to greatly improve the early detection and treatment of communicable and noncommunicable diseases (NCDs) in low-resource settings. In sub-Saharan Africa (SSA), NCDs—including hypertension, diabetes, and cancers—are increasingly contributing to the region’s overall disease burden, alongside prevalent communicable diseases such as malaria, tuberculosis (TB), and HIV [1-5]. Both communicable diseases and NCDs often remain undetected when people delay seeking care for reasons such as lack of symptoms, low illness or risk-perception, limited knowledge of available services, fear and mistrust of the public health care system, and preference for local pharmacies or alternate therapies [6-13]. Other deterrents to timely care seeking include the cost, distance, and lack of transportation to reach the clinic, especially in rural areas, and clinic-level barriers such as inconvenient clinic schedules, overcrowding, long waiting times, health care provider (HCP) attitudes, stigma and discrimination, and lack of privacy and confidentiality [6-13]. Additionally, inadequate diagnostic infrastructure (eg, skilled personnel and supplies), shortage and high cost of medicines, along with costly, delayed, or lost test results, late referrals, and uncoordinated care, contribute to misdiagnosis and suboptimal treatments, which erode public trust and discourage care seeking [6-13]. The ability to self-test, either alone or assisted by a trusted person or mobile phone apps [14,15], can decrease these persistent challenges to care seeking, facilitate timely and appropriate diagnosis and treatment, and support home-based self-care, as endorsed by the World Health Organization [16].

Despite evidence of high acceptability and feasibility of self-testing in SSA, uptake has been lower than hoped [11,17-24]. Self-testing for health conditions such as HIV is widely accepted across SSA, being relatively easy, pain-free, and minimally invasive, while providing convenience, privacy, autonomy, anonymity, safety, reduced stigma, and swift results, all of which enable self-care [14-22,24-29]. First-time testers and people with low-risk perception may also self-test out of curiosity, especially if self-tests are available at no or subsidized cost; in convenient locations such as community-based venues, schools, and youth-friendly safe testing spaces; and distributed by peers, community health workers (CHWs), and sexual partners [17,19-21,26,30-32]. Mobile health (mHealth) has further eased self-testing by providing interactive, personalized support through each step of self-testing, results capture and interpretation, health monitoring and follow-up, and linkage to care using geospatial networking apps, instructional videos, and virtual support systems [18,19,22,25,30,33]. Nonetheless, people may not self-test because of low awareness, limited accessibility, costs, stigma, lack of privacy, fear of positive results, low self-efficacy, concerns about test reliability, and challenges in navigating uncertainties, including those related to personal risk, the necessity to test, the validity of testing method, interpreting test results, and knowing what to do next [18-21,25,26,29,32-34]. Thus, self-testing implementation must be integrated within existing conceptualization of care and consider its impact on people, workflows, systems, and policies across time and contexts [18,20,24,26,31,34,35].

Ahead of a trial of different self-test implementation models, we sought to explore the acceptability of self-testing using rapid diagnostic tests (RDTs), both alone and supported by a mobile phone app, HealthPulse TestNow (Audere; henceforth referred to as the app; Multimedia Appendix 1), among public health and community leaders, HCPs, and community members in Kenya, South Africa, and Zambia. Although the study was intentionally broad to capture priority conditions, including when asking about the app and related mHealth questions, the app demonstrated self-testing for HIV and malaria. Acceptability from both provider and user perspectives is necessary for the successful implementation and use of readily available self-tests [18,20,25,31,34,35]. For this study, we drew on the Theoretical Framework of Acceptability (TFA) of Sekhon et al [36], which provides a lens to capture (1) the multifaceted nature of acceptability, (2) emotional and cognitive responses to the intervention (self-testing alone and with an app), (3) implementer and user perspectives of the intervention, and (4) temporal changes in acceptability that can occur before, during, and after intervention delivery. Thus, using the TFA allows us to identify opportunities and challenges to implementing different models of self-testing in 3 different countries and to identify and address uncertainties expressed by leaders, HCPs, and community members before implementation [35]. The findings are poised to contribute to the growing body of knowledge on the acceptability of self-testing integrated with mobile phone–based apps and provide recommendations for improving uptake of health services in low-resource settings.

## Methods

### Overview

From May 15 to August 24, 2023, we conducted an exploratory qualitative study to assess the acceptability of home-based self-testing using RDTs, both alone and supported by an app, in rural and urban settings in Migori County, Kenya; Umsunduzi Sub-District in KwaZulu-Natal, South Africa; and Luanshya District, Zambia. We conducted key informant interviews (KIIs) with community leaders and public health leaders at the Ministry or Department of Health, in-depth interviews (IDIs) with HCPs operating at health facilities within the study areas, and both IDIs and focus group discussions (FGDs) among community members living in the catchment areas of these health facilities.

### Study Sites

Over a 2-month period, qualitative research assistants from the Human Sciences Research Council, the Kenya Medical Research Institute, and Akros collected data in South Africa, Kenya, and Zambia respectively. The study sites are described below.

### Umgungundlovu District, South Africa

The study took place in Umsunduzi, a sub district within the Umgungundlovu District in Pietermaritzburg, KwaZulu-Natal, South Africa. Umsunduzi includes urban, rural, and peri-urban areas and has a population of approximately 618,536 predominantly isiZulu-speaking people. Primary health care clinics, community health care centers, and public hospitals in the area cater to the community’s health care needs. The community has a high burden of HIV, sexually transmitted infections, early pregnancy, and type 2 diabetes. Data were collected from the catchment areas of Umsunduzi, which is served by 1 hospital, 3 health care clinics, and a mobile clinic.

### Migori County, Kenya

Migori County lies in the southwest of Kenya and borders Lake Victoria and Tanzania. The multiethnic county has a population of 1.1 million, mostly living in rural areas. The county is served by 4 county hospitals and 5 subcounty hospitals. The communities are predominantly Dholuo-speaking and face high burdens of HIV and malaria. We collected data from communities and HCPs in both rural and urban settings to include all levels of the health care system and communities from diverse economic backgrounds.

### Luanshya District, Zambia

We collected data from communities in Mikomfwa and Kafubu Block, Luanshya District, in Copperbelt Province. The district has an estimated population of 212,864 predominantly Icibemba-speaking people. Mikomfwa is a highly populated periurban area, 1.4 km from the town center, with access to health facilities, schools, and internet service providers. The community is slowly developing as people transition from low- to moderate-paying jobs that allows them to build pucca houses and own cars. Kafubu Block is a rural area, located 15.7 km from the town center, populated by an older population of small-scale farmers living in thatched huts, who are served by the 1 clinic and have poor network coverage. The communities face a high burden of HIV, malaria, and early pregnancy. We collected data from the catchment areas of 2 public health clinics to include both the town-based population with easy access to health care and a rural population often living beyond easy reach of the clinic.

### Recruitment

We recruited a convenience sample of HCPs working in clinics within the study areas for the IDIs and community members living within the catchment areas of these clinics for the IDIs and FGDs. We purposefully sought public health leaders at their workplaces from among national and subnational policymakers and program implementers of programs such as those working on HIV, TB, malaria, and information, communication, and technology. Additionally, we sought community leaders such as religious, traditional, and local leaders, teachers, and area councilors from within the catchment areas served by theses clinics.

With the help of community health volunteers (CHVs) and CHWs, we sought a convenience sample of residents of the study communities aged 16 years and older who were able to read and write in either English or any of the local languages commonly spoken in the area (Kiswahili, Dholuo, isiZulu, and Icibemba). The CHVs and CHWs helped identify households with potential participants or linked the research assistants (RAs) to community gatherings or meetings for recruitment. The South Africa team also conducted community drives in busy communal locations such as taxi ranks and shopping areas to recruit community members, while the Zambia team also used house-to-house recruitment, skipping entire areas to ensure diversity and privacy. For FGDs, the teams sought representation across ages and genders, with an age range of 16 to 60 years. Once identified for an FGD, participants agreed to the details (date and time) of the discussion.

Additionally, community leaders residing in the study communities, including teachers, area councilors, and religious and traditional leaders, were identified through consultations with local leaders, CHWs, and community-based organizations. RAs met these leaders at their workspaces or residences within the study areas to assess eligibility based on roles, responsibilities, residency, literacy, and willingness to participate. The Ras provided details about the study and its potential impact on the community. Snowball sampling was used to ensure diversity in terms of community and health system position and sphere of influence.

At the health facility, the RAs shared study details with the facility leadership to identify and make appointments with different cadres of HCPs in various departments available at the facility that day. Thereafter, they invited HCPs individually to schedule appointments to receive information about the study. In all countries, eligibility criteria included being currently employed as an HCP, CHW, or CHV; being able to read and write; and providing services to test and treat NCDs and infectious diseases.

All country-teams used official government channels, including government records and relevant departments, to identify and contact potential participants from among public health leaders. Once identified through their relevant positions and organizations, RAs initiated formal contact through official appointments or written invitations, followed by phone calls or in-person visits, during which they explained the research objectives and requested participation.

### Enrollment and Data Collection

In all 3 countries, the recruiter confirmed eligibility during recruitment and the RAs on the day of the interview. After being introduced as study staff to community members at households or community meetings, RAs invited interested adults and adolescents aged 16-17 years to participate in IDIs or FGDs, per the daily field plan. Interviews with community members and leaders took place in convenient community locations, including research institutes, churches, health facilities, community halls, and homes. Groups of 4 to 7 participants aged 18-24 years and those older than 25 years, segregated by sex, were invited to attend FGDs by appointment in similar settings. RAs obtained consent and interviewed HCPs within health facilities in a private spaces and public health leaders in their offices or preferred locations at their convenience.

All participants who agreed to participate provided written informed consent or assent and brief sociodemographic information, which was captured in Research Electronic Data Capture (REDCap; Vanderbilt University) [37,38]. On the interview day, data collectors provided the informed consent sheet relevant to the participant type to ensure that participants had comprehensive study information. They also verbally provided detailed explanations, focusing on addressing questions and concerns. Participant comprehension was confirmed before obtaining informed consent for the interview and for audio recording to ensure data accuracy. For FGDs, all procedures were conducted as a group except for signing the consent form, which occurred in private after any remaining concerns and questions had been addressed out of earshot of the rest of the group.

Qualitative researchers at the Centre of Infectious Disease Research in Zambia trained the RAs in South Africa (AS), Kenya (AS, CM), and Zambia (AS, PM) through didactic sessions, role-plays, and practice sessions. They reviewed initial memos and transcripts to provide additional training as needed. The RAs had at least a high school diploma and a minimum of 3 years of experience as qualitative interviewers. They were all male in Kenya, all female in Zambia, and of mixed sex in South Africa.

The RAs used REDCap to collect basic demographic information and semistructured interview and discussion guides to explore disease priorities for self-testing and perspectives on proposed intervention elements (RDTs, self-testing, and app-assisted self-testing; Multimedia Appendix 2). The design of the interview guides aimed to answer the study questions while capturing the wider sociocultural and health systems context. In IDIs and FGDs with community members, we explored decision-making to seek health care through patient journeys, which diseases concerned the community, experiences and preference for self-testing using RDTs including assisted by a hypothetical app, and reactions to the proposed app and delivery package. In IDIs, we went in-depth into individual experience with care-seeking and diagnostic testing to understand process flows and pain points. In FGDs, we explored sociocultural norms, communal experiences, and communal reactions to the app and the possibility of using it to populate and access a digital community dashboard. In IDIs with HCPs and KIIs with community and public health leaders, we explored health concerns; experiences and preferences for home-based rapid testing, community dashboard and an app to assist with decision-making as well as entities involved in community health, rapid testing, and mHealth. Additionally, key informants provided reflections on the introduction of novel diagnostic and mHealth technologies as well as the policymaking process and its operationalization in the health system. All questions related to using the app to self-test, store and share data, provide mHealth, and create dashboards were preceded with a demonstration, taking participants through step-by-step instructions for self-testing and showing examples of data storage and dashboards.

Interviews lasted 30-45 minutes, while FGDs took 45-90 minutes and were audio recorded with participant permission.

### Data Analysis

Data were analyzed using framework analysis, which is useful for organizing large datasets and comparing findings between distinct participant groups [39,40].

First, the RAs wrote analytical memos shortly after each interview and discussion. Then, all audio-recorded interviews were transcribed, and those in local languages were directly translated into English. Once transcribed, the Ras or qualitative team members quality-checked the scripts against the original recordings in each country. During familiarization, CM and PM iteratively developed a codebook based on the RAs’ analytical memos and repeated readings of a sample of transcripts. Using NVivo (QSR International), CM and PM jointly coded transcripts for South Africa to synchronize codes and independently coded those for Kenya and Zambia (Table 1). They extracted all relevant excerpts for each of the TFA components and an emerging component of perceived benefits in Microsoft Word.

**Table 1:** The Theoretical Framework of Acceptability (TFA): constructs and definitions.

Construct number	TFA construct	Definition
1	Affective attitude	How an individual feels about the intervention.
2	Burden	The perceived amount of effort required to participate in the intervention.
3	Ethicality	The extent to which the intervention has a good fit with an individual’s value system.
4	Intervention coherence	The extent to which the participant understands the intervention and how it works, that is, whether they use apps, how often they use them, including to aid in decision-making.
5	Opportunity cost	The extent to which benefits, profits, or values must be given up to engage in the intervention.
6	Perceived effectiveness	The extent to which the intervention is perceived as likely to achieve its intended purpose.
7	Self-efficacy	The participant’s confidence that they can perform the behaviors required to participate in the intervention.
8	Perceived benefits	Perceived benefits acquired through taking part in the intervention.

Framework analyses followed the steps of data familiarization, framework identification, indexing, charting, and mapping and interpretation [39,40]. AS indexed data in Microsoft Excel by participant type and country, using inductive reasoning to identify subcomponents (Multimedia Appendix 3 provides an example). Themes within and across FGDs were further scrutinized for shared concerns, collective understandings, and divergent views to identify sociocultural norms and communal experiences. These were charted to summarize insights per TFA component by country and participant type, allowing for comparisons across and within countries and participant types, and across and within TFA components, to identify and interpret variation and clusters of data (Multimedia Appendix 3). Principal investigators and qualitative teams in each country discussed draft findings and interpretations of each construct and their interrelationships to ensure that contextual information was not missed or misunderstood. Finally, AS synthesized the results into a coherent narrative, which was reviewed and approved by all coauthors.

While pragmatic considerations determined a sample size upwards of 30, we observed meaning saturation during analyses, which aligns with estimates of a sample size of 24 interviews when assessing meaning saturation [41] and of 20-40 interviews when assessing for meta-themes across multiple countries [42]. We observed code saturation for affective attitude, burden, and perceived benefits (>250), and for intervention coherence, ethicality, and perceived effectiveness (>140). Fewer items were coded for self-efficacy (n=52) and opportunity cost (n=19) due to overlap with affective attitude, burden, and perceived benefits. However, these codes were retained for unique findings and nuances around the ability to self-test and trade-offs between risks and benefits.

### Ethical Considerations

The proposal for this study was approved by the following bodies: the Kenya Medical Research Institute’s Scientific and Ethics Review Unit (KEMRI/SERU/CMR/P00229-011- 2022/4701) and the National Commission for Science, Technology, and Innovation (950724) in Kenya; the Human Sciences Research Council Research Ethics Committee (8/23/11/22) and the Provincial Department of Health, KwaZulu-Natal, South Africa (KZ_202303_023) in South Africa; and the Where Research Ethics and Science Converge Institutional Review Board (2022-Dec-001) and the National Health Research Authority (Akros Research, NHRA-214/20/03/2023) in Zambia. The ethical bodies in Kenya and South Africa granted a waiver for parental consent for adolescents aged 16-17 years. Additional permissions were granted by the County Health and Management Team and Migori County Research Department in Kenya, and the Copperbelt Provincial and District Health Offices in Zambia.

As described above, all participants in the 3 countries provided written informed consent, or assent in the case of those aged 16-17 years, with a waiver of parental consent applied in South Africa and Kenya. All presented data are anonymous and deidentified, with quotes labeled as “country, group.” All participants received time or transport reimbursements in cash or goods (oil, soap) equivalent to approximately US $7.00, with refreshments provided during FGDs. This manuscript meets the requirements of the Consolidated Criteria for Reporting Qualitative Research (COREQ; Multimedia Appendix 4) [43].

## Results

### Overview

Across the 3 countries, we conducted 24 KIIs with community and public health leaders, 41 IDIs with HCPs, and 55 IDIs and 11 FGDs with community members (Table 2). Of the 11 FGDs, 5 were conducted in South Africa, consisting of 4-5 participants each; 2 in Kenya, with 7 participants each; and 4 in Zambia, with 5 participants each.

**Table 2:** Number of participants in the qualitative study by interview type in Kenya, South Africa, and Zambia, May 15 to August 24, 2023. There were 11 focus group discussions (FGDs) in total.

Participant category	Interview type	Number of participants, n			
		Kenya (n=47)	South Africa (n=70)	Zambia (n=61)	Total (N=178)
Community and public health leaders	KIIs^a^	5	7	12	24
Health care providers	IDIs^b^	12	15	14	41
Community members	IDIs	16	24	15	55
Community members	FGDs	14	24	20	58

^a^KII: key informant interview.

^b^IDI: in-depth interview.

Most participants were community members (113/178, 66%). About half were female and between the ages of 25 and 44 years, one-third were unemployed, and more than one-third of the 42 HCPs worked as nurses or midwives (Table 3). While Kenya had fewer participants overall (47/178, 26%), South Africa had the most participants who were female (41/70, 59%), community members (48/70, 69%), and older than 45 years (24/70, 34%), whereas Zambia had the most community and public health leaders (12/61, 20%) and participants aged 25-30 years (37/61, 61%).

**Table 3:** Sociodemographic data of participants in the qualitative study by interview type in Kenya, South Africa, and Zambia, May 15 to August 24, 2023.

Category		Kenya (n=47)	South Africa (n=70)	Zambia (n=61)	Total (N=178)
**Method, n**					
	Focus group	14	23	20	57
	In-depth interview	33	47	41	121
**Participant type, n**					
	Community member	30	48	35	113
	Provider	12	15	14	41
	Community and public health leaders	5	7	12	24
**Gender, n**					
	Female	18	41	28	87
	Male	29	29	33	91
**Age (years), n**					
	16-17	0	7	0	7
	18-24	12	12	12	36
	25-34	9	14	23	46
	35-44	15	13	14	42
	45-54	8	16	8	32
	55-64	3	7	4	14
	≥65	0	1	0	1
**Occupation (health care providers), n**					
	Nurse	3	9	2	14
	Midwife	0	0	2	2
	CHW^a^ or CHV^b^	2	3	1	6
	Clinical officer	3	0	1	4
	General doctor or specialist	2	0	0	2
	Laboratory technician	1	0	1	2
	Other	2	3	7	12
**Occupation (other participants), n**					
	Farming or agriculture	12	0	7	19
	Other manual labor	4	1	0	5
	Office or clerical work	1	0	0	1
	Small-market sales or trade	2	1	1	4
	Housewife	1	0	1	2
	Student	3	8	1	12
	Unemployed	2	33	17	52
	Other	5	5	13	23

^a^CHW: community health worker.

^b^CHV: community health volunteer.

### Priority Diseases for Rapid Testing

In all 3 countries, participants’ list of diseases they would prioritize for rapid testing showed awareness of the double burden of communicable and NCDs. Among the top 8 conditions mentioned, HIV and sexually transmitted infections were consistently identified as priorities across the 3 countries. Malaria was frequently mentioned in Kenya and Zambia, whereas TB featured more prominently in South Africa and Zambia than in Kenya. Mention of diabetes (blood sugar) and hypertension (blood pressure) testing in all 3 countries highlighted concerns about NCDs. Cancer and pregnancy tests received more mentions in Kenya and South Africa than in Zambia.

### Acceptability

In the following sections, we present the findings with regard to self-testing according to the TFA constructs: (1) affective attitude (overall feeling), (2) burden (level of effort), (3) ethicality (alignment with values), (4) intervention coherence (understanding the intervention), (5) opportunity cost (trade-offs), (6) perceived effectiveness (benefits), and (7) self-efficacy (confidence in ability to self-test). Multimedia Appendix 3 contains select quotes and intensity mapping for each subcomponent, followed by those between components.

#### Affective Attitude

All groups—community and public health leaders, HCPs, and community members—in the 3 countries expressed positive feelings towards self-testing, both alone and assisted by a mobile phone app, as a “very good thing in the community” that “would be wow” for improving individual and population health. They marveled at the “beauty” of self-testing, alone and with the app, in increasing efficiencies and rapid decision-making for both patients and HCPs. Community members strongly trusted self-testing results, with one Zambian participant emphasizing that “these things do not lie.” Community members trusted self-test results that they could see for themselves for conditions such as HIV, diabetes, or malaria, rather than relying on HCPs’ words or laboratory reports, with a Kenyan youth providing the example of self-testing that confirmed her suspicion of a false-positive laboratory pregnancy test. Community members and leaders appreciated the app’s ability to bring health care “closer” and empower community members to manage their own health. In Zambia and Kenya, community members further associated app-assisted self-testing with modernity, emphasizing its place “in a modern world” and “digital life,” as well as noting that “the world is moving towards technological solutions.” Public health leaders and HCPs in all countries also expressed enthusiasm and optimism for the app’s ability to streamline processes and improve the accuracy and speed of diagnosis and data collection. Nonetheless, participants anticipated pockets of resistance from those accustomed to old ways—for example, among HCPs and older people—who would resist “new” things, especially if they did not have smartphones or were not used to social media apps such as Facebook, TikTok, and WhatsApp. In South Africa, one community member suggested that some people may object to apps saying, “That is white people business; white people are playing with us,” a statement that, although expressed by only one community member, suggests possible racial sensitivities in this context. In contrast, Kenyan community members trusted the proposed app because they felt that, like other existing apps, it would be developed by skilled and learned people.

#### Burden

Self-testing was generally considered “simpler, shorter, and easier” to implement with minimal disruption to daily life. Participants agreed that with proper instruction, support, and simplicity, using the app would be “a walk in the park,” with a Zambian community leader noting that most people, even in rural areas, had access to mobile phones. Participants thought that although self-testing could be complicated due to multiple steps, the type of biospecimen (eg, sputum for TB), and disease perception (severity and stigma), the written and visual step-by-step instructions in the app could guide novice users through self-testing and subsequent steps, such as waste management, home care, or seeking an HCP. HCPs also thought that an app would make real-time data entry and access easier. Communities considered the moderate effort required to set up the app worth the cost savings and efficiency gains. They perceived the app as user-friendly “if guided by the guidelines” or “if [users were] properly educated.”

When asked, participants described the effort required to fulfill their role in cascading self-testing from the national to the household level. Public health leaders anticipated substantial time and resource demands to establish policies, coordination mechanisms, and infrastructure, including roads, tools, supply chains, data safety and monitoring, and continuous training. Kenyan public health leaders emphasized user compliance, biomedical waste management, and motivating CHVs and, along with Zambian public health leaders, accountability mechanisms. South African public health leaders also advocated for continued research and development for conditions such as TB, where self-testing methods are not yet fully developed. Additionally, health facilities may bear increased costs of training, procurement, and distribution. HCPs felt they would have to bear the greatest responsibility until data integration into health systems was completed. They, along with community leaders, thought that people may need time and money to acquire RDTs, which may not be available within their vicinity, and may need to surmount their emotions to conduct and constructively act on test results, particularly for sensitive conditions such as HIV.

When asked about the effort required to include an app to assist with self-testing, public health leaders and HCPs foresaw required national- and local-level planning, coordination, and subsidies to mitigate poor network coverage, limited phone ownership, and the costs of airtime, data bundles, and electricity supply in some parts of each country. Furthermore, app implementation would require skilled personnel to ensure functionality and data integration into existing workflows. Adequate community-based staff would be needed to respond to app-generated information and requests while safeguarding against misuse. HCPs saw a need to exert moderate effort to acquire devices, connectivity, data bundles, and user support. They anticipated significant effort to train and support novice or digitally illiterate users alongside HCPs already accustomed to other systems (such as SmartCare in Zambia), who may be resistant to the steep learning curve and the added work of entering app-generated data, especially if it was perceived to distract from direct patient care. Communities anticipated challenges with poor connectivity, difficulties in learning to use the app, and poor phone battery life.

#### Ethicality

All groups in the 3 countries broadly agreed that self-testing aligned with personal and collective values of privacy, convenience, empowerment, and autonomy that underpin effective health care, aspects further enhanced when assisted by the app. They thought that app-assisted self-testing allowed persons to take personal responsibility for managing their own health, with some Kenyan HCPs stating that self-testing was a “game changer” as it shifted from “clinician-driven” to “individual-driven” health screening, which allowed people, even in remote and underserved areas, to be the “first to know” and act on screening results for conditions like malaria or HIV. Participants said that users would feel “free,” “safe,” and “not scared” of their “secret” being known and causing shame, expressed as, “What are the people in the community going to say about me?” by a Kenyan community member. In South Africa, an app was seen as especially beneficial for men, who often feel uncomfortable discussing sensitive health issues. Participants believed that anonymous access to counselors and doctors and home-delivery of medication would help assure confidentiality, with a Kenyan community member noting the benefit of remote health support in rural, flood-prone, and war-torn areas.

When asked about possible challenges and concerns, participants highlighted potential ethical dilemmas during implementation. Public health leaders and HCPs cautioned that repeated and unnecessary testing, for example, when people doubt the results of the first test, could deplete testing kits, denying those who need them and allowing for malfeasance such as selling counterfeit and used RDTs. Improper storage and disposal of self-testing kits could further raise issues of child safety. Also, self-treatment based on screening results could deprive individuals of additional tests and clinical examination needed to make a diagnosis and uncover other conditions while increasing severity and spread of disease. Furthermore, options for filling prescriptions over an app without a physical examination could violate professional ethics and regulations and reduce social interactions arising from a clinic visit. All groups thought that unexpected results, such as a positive HIV self-test, had potential for causing emotional distress, suicidal ideation, and marital rift. Lack of privacy when self-testing or data breaches could also garner unsolicited medical advice by “Dr. Neighbors,” stigma, and marital issues, leading to the suggestion for a delete feature on the app by South African community members. When implementing an app, all groups advocated for design features that included those with disabilities, such as visual impairment, no or low literacy, “dumb phones,” poor connectivity, as well as non-English speaking and rural populations. HCPs and community members stressed the importance of education, social support, and trust within families and communities so that everyone could benefit equally. In Zambia, an HCP advocated for the cultural value of intergenerational care where the older generation imparts knowledge to younger ones on importance of self-testing, who, in turn, help the older adults use new technology.

#### Intervention Coherence

Participants saw the relevance of self-testing given insufficient health systems and the demands of daily life in under-resourced settings and were familiar with HIV self-testing and malaria (Kenya and Zambia) RDTs. They generally knew the processes of self-testing, related paraphernalia, and the possibility of needing repeat tests, such as for HIV—all of which could differ by disease—and the results of which could help people decide when and where to seek care. For example, they suggested phoning in questions when testing positive for pregnancy or COVID-19 and in-person consultation for chronic conditions like HIV or cancer to facilitate counseling and support. Public health leaders and HCPs could speak about RDTs in medical terms, for example, using terms such “species of malaria,” “false positive,” “false negatives,” and “antigens,” while laypersons may use terms such as “drops of water” to describe reagents. The groups were less conversant with apps and the technical details of how an app functions. All groups emphasized a communal approach to technology adoption to foster collective understanding and support and lead to “openness” and “cooperation,” with a Kenyan community member offering the example of spouses collecting medication for each other after an app-based doctor consultation.

#### Opportunity Costs

All groups perceived self-testing as significantly reducing opportunity costs through time saving, convenience, and improved prevention and care-seeking practices, especially for those who might otherwise delay or forgo testing due to time and logistical constraints. Community leaders and members foresaw minimal sacrifices, appreciating the efficiency gains and ease of integration into daily life. Community members particularly favored the option to buy medicine from a chemist and to receive home visits, which they perceived would free up time for productive activities. When probed for cons, public health leaders highlighted that implementation of app-assisted self-testing could be at the cost of some established practices, HCPs indicated the possibility of job redundancies, and community members cited the cost of purchasing RDTs and apps.

#### Perceived Effectiveness

All groups perceived self-testing as an effective tool for improving access to health care, enabling early diagnosis and treatment, and reducing the overall burden on the health care system. According to public health leaders and HCPs, self-testing could maintain healthier populations and decrease the severity and spread of disease by facilitating early diagnosis, timely care-seeking and treatment, and self-monitoring practices. For example, one South African HCP described how home-based HIV self-testing by pregnant women could detect missed or new HIV infection, prompting antiretroviral therapy and preventing transmission. Participants agreed that self-tests enabled immediate and correct action, which was especially beneficial in remote areas with poor access to health care and for conditions like malaria (Kenya and Zambia), where rapid progression can occur. All agreed that by reducing unnecessary visits, self-testing saved money, eased clinic congestion and wait times, and enhanced privacy. HCPs noted that it released time to attend to other and more complex cases.

According to public health leaders and HCPs, the addition of an app had the potential for two-way information exchange to supplement guidance and diagnostic tools, improve individual- and population-level disease trend tracking, facilitate quicker access to care, and enable shared decision-making through access to real-time data. HCPs remained optimistic that an app would further streamline processes, improve resource allocation, and enhance the accuracy and speed of diagnosis, data recording, reporting, and retrieval processes—ensuring more timely, precise, and coordinated care plans. Community leaders agreed that an app could provide specific and accurate diagnoses based on inputted data, issue immediate advice, and schedule follow-up actions (such as medicine delivery), thereby making health services available “from the palm of my hands.” Participants concurred that the app could increase the effectiveness of self-tests by providing immediate access to advice, education on self-management, and the means to monitor progress, with added benefits of privacy and protection from nosocomial infections. Immediacy and the level of user support were considered critical to realizing the benefits of app-assisted self-testing, with Kenyan community members suggesting that linking the app to hospital services would streamline processes to get “services faster,” and those in South Africa suggesting that designated personnel could answer questions and migrate app-based data to hospital records.

When probed, all groups said that self-testing had potential for false readings due to human error, including in handling specimens and waiting time; malfunctioning devices, such as those for blood pressure or blood sugar readings; and the accuracy of RDTs, especially for diseases with multiple strains like malaria. HCPs speculated that false positives and overlooked comorbidities could deteriorate health and increase costs of follow-up care. However, most felt that the benefits outweighed the risks, with a Kenyan HCP saying that “You know, there are many cases that will be identified that would go like, missed.” Additionally, most participants thought that people should link to care for further tests and evaluations when screening positive for conditions such as HIV and pregnancy (requiring antenatal care).

#### Self-Efficacy

All groups were confident in individuals’ ability to self-test especially if “everything will be guided through the app.” When probed, they cited the possibility of human error, especially for complicated test procedures and among people with limited education, even after training. Participants suggested that regular training, practice, and guidance through HCPs or an app could be useful for less experienced users and to address any misgivings. Community leaders had confidence that app features linking individuals to counseling, treatment, and follow-up could successfully replace or supplement traditional methods of receiving and following medical instructions. Community members’ confidence in performing app-assisted self-testing and linking to care depended on familiarity with and guidance provided by an app, professional input and confirmation of results, and technical and resource barriers. However, unexpected test results could raise self-doubt, for which they would want reassurance from either family members or HCPs.

## Discussion

### Principal Results

We found high acceptability toward self-testing, both independently and assisted by an app, across the 3 countries. All groups—community and public health leaders, HCPs, and community members—expressed a generally positive and favorable attitude toward self-testing (affective attitude). This positive attitude arose from the perceived relevance in the context of high disease burden, overwhelmed health systems, insufficient laboratory services, and shaky public trust (intervention coherence); the ability to continue with priority activities (reduced opportunity costs); and increased access to correct diagnosis and treatment (perceived effectiveness). Participants felt empowered due to the perceived benefits of relieving under-resourced health systems; the anticipated professional satisfaction among HCPs; and the cost saving, convenience, privacy, and autonomy (ethicality), along with ease of use (self-efficacy, burden). The addition of apps to assist with self-testing only enhanced these perceived benefits and brought the additional excitement of novelty and modernity that suggested willingness to embrace change, especially in Kenya and Zambia. However, concerns about inclusivity and equity in reaching households tempered the enthusiastic reception of self-testing, alone or assisted by an app. These concerns were closely intertwined with perceived infrastructural, logistical, and technological challenges that could hinder the uptake of self-testing.

The strongest cross-cutting enablers of uptake arising from this study were autonomy, efficiency, digital appeal, and trust in RDTs, while barriers included digital exclusion, emotional risk, data privacy, and system integration challenges. Country-specific differences underscored the need for localized strategies. In Kenya and Zambia, there was stronger enthusiasm for digital self-testing as a sign of progress and modernity, whereas South African participants expressed greater sociopolitical skepticism. South Africa also emphasized structural constraints such as unreliable internet, limited smartphone access, and energy insecurity due to load shedding more than the other countries. While Kenyan participants frequently cited CHVs as trusted local figures who could support uptake, Zambian participants highlighted intergenerational support systems, particularly the role of youth in helping older adults use apps. Legal and ethical constraints were especially noted in Zambia, where national officials raised concerns about conflicts between app-based care and national policies requiring in-person assessments before prescribing medications—issues less explicitly discussed in Kenya or South Africa. Lastly, Kenyan and Zambian community members were more optimistic that with appropriate training, most people could learn to use the app effectively, whereas older South African participants expressed deeper doubts about digital readiness and ownership of suitable devices.

Our findings revealed priority communicable diseases and NCDs and suggested that early engagement with app-assisted self-testing tools is most likely among youth, urban residents, and those already familiar with mobile apps or self-testing kits, particularly in Kenya and Zambia. However, broader adoption is possible through intentional, community-based strategies that include intergenerational support, culturally appropriate messaging, offline functionality, and integration with trusted health care systems and CHW networks. These insights have informed our ongoing randomized controlled trial. We have prioritized self-testing for HIV, malaria, and pregnancy for which self-tests or RDTs are available. Additionally, our study teams are testing for hypertension at study visits. The study provides all enrolled household members with education on when to self-test. Each household is also assigned to 1 of 3 self-testing locations: the health care facility, at home assisted by a CHW, or at home assisted by a print-out providing step-by-step instructions downloaded from the HealthPulse App developed by Audere. The CHW uses this app to guide them through providing home-based testing during study and interim visits. If desired, households with smartphones could also download the app—designed to work offline—to receive step-by-step instructions and store test results. At the end of the 6-months trial, we will conduct IDIs to learn from households’ experiences with accessing diagnostic and treatment services.

### Comparison With Previous Work

Unique to this study, Zambian communities emphasized not only the importance of younger people helping older adults with technology but also of parents teaching and habituating children to self-testing. Participants in Kenya also uniquely mentioned waste management as the user’s responsibility—an aspect often overlooked in the literature on self-testing [25,35,44].

Though highly accepted, people may not self-test alone or assisted by an app due to the practical challenges identified in this study, which could replicate and perpetuate ongoing societal inequities [15,18,24,44-46]. Younger, digitally literate individuals, particularly those living in urban and periurban areas, emerged as the groups most likely to benefit from app-assisted self-testing tools across Kenya, Zambia, and South Africa. Additionally, community members facing long travel times or stigma at clinics, such as young women seeking pregnancy- or HIV-related services, saw clear advantages in managing their health at home. However, when responsible for testing and linking to care, some populations—such as adolescents, women, and the older adults—may lack the required spousal, parental, familial acceptance, guidance, and financial or practical support [15,24,46-48]. Participants also identified older adults, individuals in rural or remote settings, those with limited education or digital literacy, and people without smartphones or consistent access to electricity and mobile data as less likely to benefit. Other studies corroborate that people living in poverty and distant, rural areas may not have the means to access RDTs, apps, and medication [11,15,44-47]. In Zimbabwe, although young men had higher rates of app-assisted HIV self-testing than young women due to internet access, overall use was low due to shared phone ownership, provider-blocked phones, and intermittent electricity and internet supply [18]. Our participants anticipated these same barriers and the exclusion of non-English speakers, people with low literacy, disabilities, and those less technologically experienced if apps and test kits are not appropriately adapted [14,15,18,46,49]. While South African participants anticipated increased greater health care access for men through self-testing, a meta-analysis on HIV self-testing found low uptake among men with mobile jobs, who were less aware of self-testing options, or skeptical about test accuracy in SSA [50]. Lack of domestic privacy may also limit ability to test at home [33,48]. Thus, the deployment of self-testing will require equity-focused, reflexive approaches that combine technological and traditional mechanisms and support systems to minimize bias and maximize impact for subgroups [14,15,18,21,28,35,45].

Our findings confirm that unexpected results can lead to mistrust, often due to denial of conditions such as HIV or fear of alternate diagnoses and treatments when testing negative for easily treated conditions like malaria [11,18,44]. This reaction may stem from the emotional and cognitive burden of self-testing, as individuals must be psychologically ready both to test and to receive the outcome, especially for conditions such as HIV [14-22,29-35,49-51]. Unexpected results, the use of different specimen collection methods (eg, blood or saliva), and the introduction of new RDTs entering the market can affect people’s perception of the reliability of self-tests, their self-efficacy, and their interpretation of test results [19,22,34,44,46,50]. Being assisted by or trained by HCPs at first use can be a successful strategy to ensure user understanding and autonomy and reduce the anxieties expressed by participants [30,31,44]. This aligns with participants’ request for education, training, and ongoing support for both HCPs and self-testers [11,24,44]. Participants across all health system levels in the 3 countries advocated for inclusive implementation strategies, including app design adaptations such as the use of local languages, audiovisual instructions, offline functionality, and simplified interfaces. As previously mentioned, intergenerational support, in which tech-savvy youth assist older adults, emerged as a promising approach in Zambia. Additionally, participants suggested that trusted peer champions and early adopters could help boost uptake through word-of-mouth and demonstration. These suggestions for user-friendly RDTs, culturally sensitive instructions, and multimodal support systems—including printed, phone-text, video-based, live support, and in-person guidance—could mitigate existing health and digital inequities and hold strong potential to improve access, empower users, and ease health system burdens across diverse settings and populations [14,15,18,19,21,28,44,45,51].

Access to and use of self-testing technologies can be increased by involving private, community, nongovernmental, and governmental partners. Distribution through peers, intimate partners, community members, drugstore vendors, and online platforms shows promise for providing self-tests, counseling, and case management [11,19,46]. However, financial interests may drive unnecessary testing and medication dispensing by the private sector [11,44] if methods to incentivize proper practices are not in place [52]. Our participants also suggested targeted training, especially through CHWs. Reliance on public sector CHVs or CHWs can have mixed receptions due to issues of competency, confidentiality, privacy, familiarity, and stigma [15,24,31]. Also, while advocating for face-to-face counseling [19,22,50], a Kenyan study found that few participants accessed it, possibly because of its repetitive, frustrating, intrusive, and time-consuming nature [31]. Such ongoing barriers to linkage to care, difficulties in communicating results, and poor relations observed between community- and facility-based HCPs highlight the need for a safe, monitored pathway through national integration and health system adaptation to suit users [14,15,19,22,24,28,35,44,46,51].

In all 3 countries, public health leaders must address policy, infrastructure, and resource allocation to integrate self-testing and achieve public health outcomes [19,44,46,48]. This includes building supportive infrastructure, ensuring scalability, maintaining test quality, and implementing safe data management systems to track and respond to disease trends, user compliance, and care quality [14,15,22,48]. Tailored campaigns should address concerns about accuracy, emotional distress, and ethics to ensure acceptance [24,51]. HCPs will play a critical role in implementing culturally sensitive strategies and ensuring sensitive data management to avoid biased statistics, privacy breaches, and public health gaps [14,15,19,44,50,51]. Respondents in this study also emphasized the importance of subsidies or low-cost options for test kits and mobile data, integration with existing health care systems, and hands-on community sensitization campaigns. Other studies corroborate that communities will need accessible commodities, tools, education, and trust-building efforts to support adoption, with clear links to professional care and communal support structures [14,24,35,50,51]. Gaps in transport, digital, and data management infrastructure to reach remote areas will require a multisectoral response involving ministries of finance, planning, development, and information, communication, and technology, increasing costs in the short to medium term and slowing down the cascade of home-based self-testing [14,15,24,35,47,52-55].

### Strengths and Limitations

The strengths of this study lie in its comprehensive exploration of diverse perspectives across multiple countries and stakeholder groups, providing a holistic understanding of the acceptability of app-assisted self-testing [36,55]. The use of the TFA enables a multidimensional analysis of the intervention’s acceptability to highlight how, despite positive perceptions of self-testing and app-assisted self-testing, burdens, effectiveness concerns, ethical issues, and self-efficacy interact to shape overall acceptability [36]. Each factor influences the others, creating a complex picture in which enthusiasm is tempered by practical challenges, particularly in implementation and support systems, raising ethical dilemmas that must be addressed. However, the study also has limitations, including potential biases in self-reported data, such as social desirability bias, and the fact that interviews did not follow the actual use of app-assisted self-testing and thus captured perceptions rather than real-world experiences [55].

### Conclusions

This study underscores the strong acceptability and potential of self-testing, both independently and with app-based support, as an empowering, efficient, and increasingly relevant public health tool across Kenya, Zambia, and South Africa. Participants across stakeholder groups viewed self-testing favorably, especially considering overburdened health systems, diagnostic gaps, and the desire for autonomy, privacy, and timely care. However, this enthusiasm was tempered by valid concerns about equity, digital exclusion, infrastructural limitations, emotional readiness, and regulatory constraints. The findings emphasize the need for context-specific, equity-focused implementation strategies that blend digital innovation with community engagement, intergenerational support, and integration into trusted health care systems. Investments in offline functionality, culturally appropriate education, inclusive app design, and supportive CHW networks are critical to ensuring that self-testing reaches and benefits the most marginalized. To sustain impact and minimize harm, public health systems must not only adapt policies and infrastructure but also prioritize safe, ethical, and user-centered pathways that link self-testing to timely, high-quality care. By balancing innovation with inclusivity and community-led solutions with systemic support, self-testing and app-assisted approaches can transform diagnostics and care delivery in ways that are responsive to both the opportunities and the realities of diverse settings.

## Appendix

Multimedia Appendix 1 Sample screen shown to participants of the Healthpulse Testnow (developed by Audere).

Multimedia Appendix 2 Interview guides.

Multimedia Appendix 3 Indexing, charting and mapping of data.

Multimedia Appendix 4 COREQ Checklist.

## Abbreviations

CHV: community health volunteer
CHW: community health worker
COREQ: Consolidated Criteria for Reporting Qualitative Research
FGD: focus group discussion
HCP: health care provider
IDI: in-depth interview
KII: key informant interview
mHealth: mobile health
NCD: noncommunicable disease
RA: research assistant
RDT: rapid diagnostic test
REDCap: Research Electronic Data Capture
SSA: sub-Saharan Africa
TB: tuberculosis
TFA: Theoretical Framework of Acceptability

## Data Availability

The datasets used and/or analyzed during this study are available from the corresponding author on reasonable request.
