# Supplementary material for: Perspectives on App-Assisted Self-Testing Using Rapid Diagnostic Tests Among Community Members, Health Care Providers, and Public Health Leaders in Kenya, South Africa, and Zambia: Qualitative Study
Source: J Med Internet Res. 2025 Nov 26;27:e70273. doi: 10.2196/70273 (PMC12696451; doi:10.2196/70273)
Supplement: Multimedia Appendix 1 [file jmir_v27i1e70273_app1.pdf]

HealthPulse TestNow: Example app screens

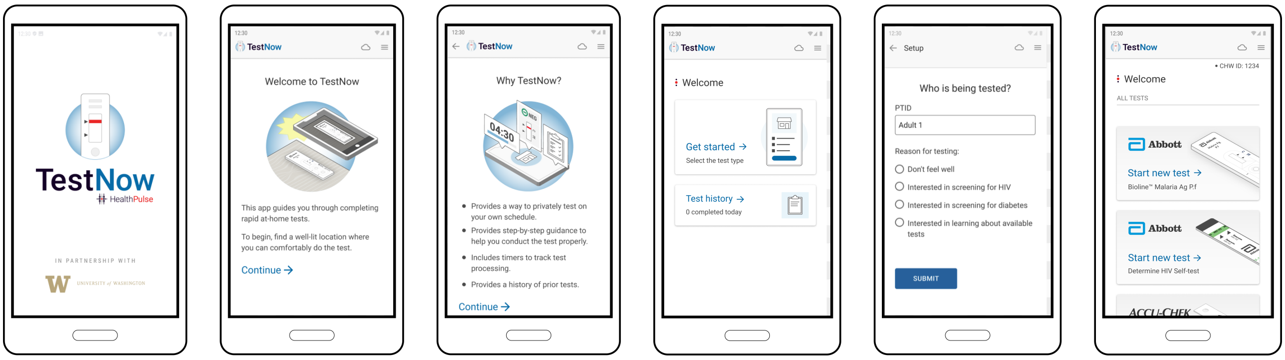

App initiation/loading

Welcome
Welcome patients to the TestNow app and describe the app's purpose.

Home screen
Patients can start a new test or view past results.

Patient setup
Patient and test reason are identified.

Select RDT
Patients selects from list of tests.

Tests:

- Malaria
- HIV
- Blood glucose

Malaria test

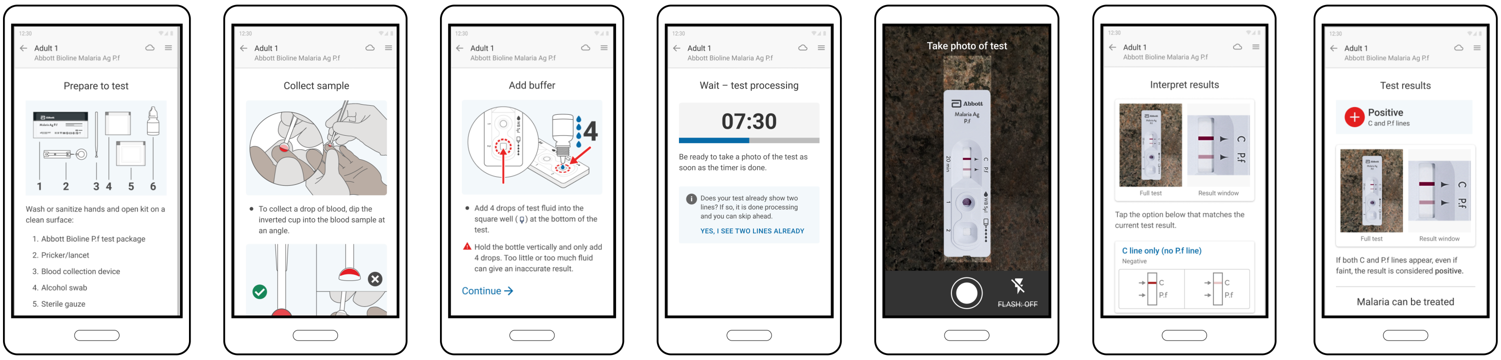

Malaria test administration steps
Sample of visual step by step instructions supporting the patient to properly conduct the test.

Test processing timer
Supports reading results at the right time.

Test photo capture
Directs photo capture of test on mobile device.

Interpret results
Supports patient interpretation of test result.

Test results & next steps
Guides next steps for patient based on result.

HIV test

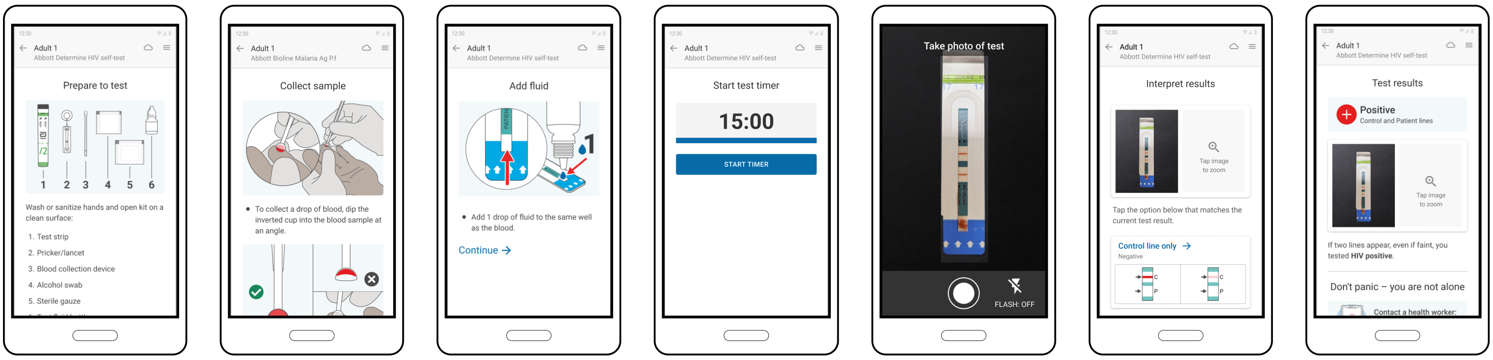

HIV test administration steps
Sample of visual step by step instructions supporting the patient to properly conduct the test.

Test processing timer
Supports reading results at the right time.

Test photo capture
Directs photo capture of test on mobile device.

Interpret results
Supports patient interpretation of test result.

Test results & next steps
Guides next steps for patient based on result.

Blood glucose test

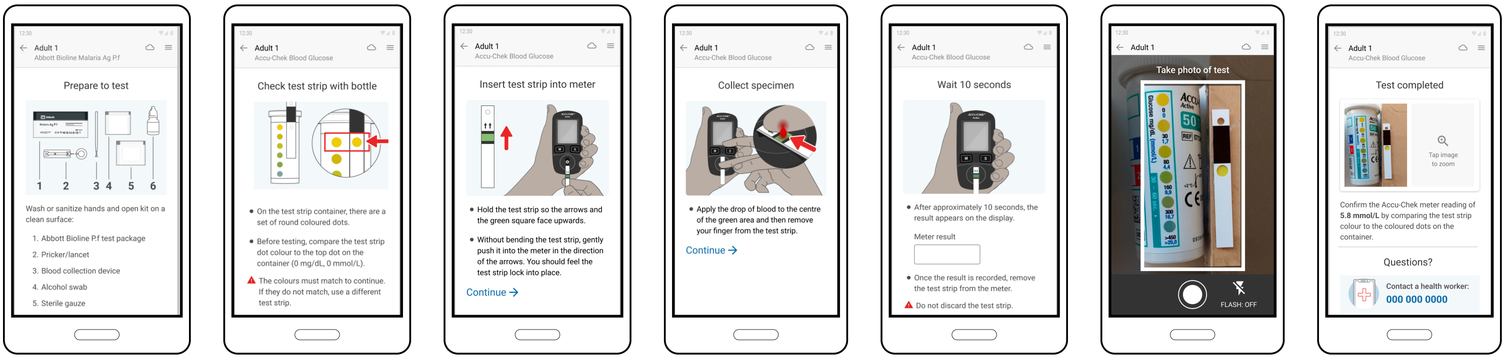

Blood glucose test administration steps
Sample of visual step by step instructions supporting the patient to properly conduct the test using the blood glucose meter.

Record meter result
Record result from meter in the app.

Test photo capture
Photograph test beside reference scale.

Test results & next steps
Guides next steps for patient based on result.
