# Supplementary material for: Perspectives on App-Assisted Self-Testing Using Rapid Diagnostic Tests Among Community Members, Health Care Providers, and Public Health Leaders in Kenya, South Africa, and Zambia: Qualitative Study
Source: J Med Internet Res. 2025 Nov 26;27:e70273. doi: 10.2196/70273 (PMC12696451; doi:10.2196/70273)
Supplement: Multimedia Appendix 2 [file jmir_v27i1e70273_app2.zip › Multimedia 2 DASH interview guides/4. Interview Guide_Providers_V0.5_09042023.docx]

**INTERVIEW GUIDE**

**Healthcare Providers**

**PURPOSE**

One aim of this interview guide is to understand the preferences, feedback, and perspectives of health care providers in the healthcare system to guide a proposed mobile health delivery intervention package.

**INSTRUCTIONS**

There are 2 levels of questions:

• **Numbered questions (1, a, etc):** these questions **must be asked** and discuss with participants.

• **Bulleted Probes:** to serve as suggestions for the facilitator rather than a strict list of questions that *must* be asked. So, **depending on what has already been discussed, and the IDI context, you may ask these probes or not or may phrase probes differently** to try and better understand what the participant is trying to communicate.

**MATERIALS**

1. App phone demo or print-out (**2-3 sets** **to be laminated and shared between participants**)
2. Audere assets last 4 pictures (specific to HCPs)

- Instructions/suggestions to facilitator are in *italics*.

**Background**

1. What kind of work do you do in this healthcare facility?
   - Main responsibilities
   - What has been your experience with delivering testing or treatment services?

**Acceptability of home-based testing**

1. What are your views and perceptions about home-based rapid self-testing for various conditions in the community that you serve?
2. What impact would the introduction of home-based rapid self-testing have on your role as a health care provider?
   - What changes would the use of home-based rapid self-testing have on your work (*role, workload, workflow*)
   - How does the prospect of the changes in your role/work make you feel?
3. How would introducing rapid tests benefit the healthcare system?
   - The facility you work in?
4. What disadvantages could the healthcare system experience if people self-test for various conditions?
   - To this facility?

**Self-test for communities**

1. How do you feel about making these rapid tests available for self-testing in the communities that you serve? *For e.g., TB*. *If unable to articulate, use the example of HIV self-testing)*
   - What would be the benefits of introducing rapid self-testing for the people of the community that you serve? (*Time, cost, etc*.)
   - What limitations would the introduction of rapid self-testing have for the people in the community you serve?
   - What concerns do you have about making home-based diagnostic tests available for the people in the community that you serve? (*Ability, trusting results, misuse, regulatory framework*)
2. For which conditions do you think home-based diagnostic tests would be suitable? (*Probe: HIV, diabetes, hypertension, malaria, pregnancy, STIs, etc*.)
3. In order of priority, which top 3 diseases do you think should be prioritized for rapid testing in the communities that you serve, and why?
4. What resources would the people in your community need in order to start self-testing? (*Information, venue, cost, etc.)*
5. What support would the people in the community you serve need to be able to take up self-testing?

a. Conduct the self-test

b. Read and interpret the results

c. Process the diagnosis

1. Which community venues would be suitable for self-testing for the people in the community that you serve?

**Post self-testing considerations**

1. What do you think would be the most efficient and effective steps after someone has self-tested themselves? By efficient I mean the least number of steps for both you and the patient, and by effective, I mean the optimal health outcome.

**We are trying to put a package of interventions that would provide rapid diagnostic tests to people at home, with an app to help them conduct the tests and store their test results. Here is the prototype of the app**

[**Interviewer,** *please show the phone demo of the app*]

1. What are your thoughts on a patient producing results from an App such as the prototype you were shown, and seeking medical advice from you as a healthcare provider?
   1. What purpose would it achieve?
   2. How would informing the clinic with rapid self-test results from an App benefit the healthcare system compared to without an app? This facility? The patient?
   3. What is the risk to the healthcare system if people get medical advice for various conditions through an app? To this facility?
   4. What are the limitations of app-based medical consultation? (*accuracy of results*, *missing physical examination*)
2. What do you think about the patient using an app to get a prescription?
3. What do you think about the patient using an App to order medication for home delivery?
4. What do you think about patients having access to their medical records, for e.g., test results through this app?

**Mobile Apps, Data and Systems**

[**Interviewer,** *please go to the last 4 pictures in the Audere assets. Show the first 1 phots of the set for HCPs*]

1. What do you think of an app where patients can enter:
   - 1. Symptoms/reasons for self-testing?
     2. Self-test results?
     3. Self-report taking the medicine?
     4. Their geo-location?
2. How would all this information be entered into the DHIS dashboard?
3. Can you tell me about the centralized electronic medical systems in use in your [***facility*** *for providers /* ***national health system/jurisdiction*** *for stakeholders*]?
   1. What kind of data does it contain? (*Clinical, pharmacy, lab, administrative, financial*)
   2. Where does this data come from (*health facilities, community health workers, other sources – for e.g., pharmacies or weather stations or community organizations?)*
   3. What are some kinds of data that are not currently collected, but which you think would be useful?
      1. How would you use this data?
