# Supplementary material for: Perspectives on App-Assisted Self-Testing Using Rapid Diagnostic Tests Among Community Members, Health Care Providers, and Public Health Leaders in Kenya, South Africa, and Zambia: Qualitative Study
Source: J Med Internet Res. 2025 Nov 26;27:e70273. doi: 10.2196/70273 (PMC12696451; doi:10.2196/70273)
Supplement: Multimedia Appendix 2 [file jmir_v27i1e70273_app2.zip › Multimedia 2 DASH interview guides/6. Interview Guide_Community Stakeholders_V.03_09042023.docx]

**INTERVIEW GUIDE**

**Community Stakeholders**

**PURPOSE**

One aim of this interview guide is to understand the preferences, feedback, and perspectives of community stakeholders in the healthcare system to guide a proposed mobile health delivery intervention package.

**INSTRUCTIONS**

There are 2 levels of questions:

• **Numbered questions (1, a, etc):** these questions **must be asked** and discuss with participants.

• **Bulleted Probes:** to serve as suggestions for the facilitator rather than a strict list of questions that *must* be asked. So, **depending on what has already been discussed, and the IDI context, you may ask these probes or not or may phrase probes differently** to try and better understand what the participant is trying to communicate.

**MATERIALS**

1. App phone demo or print-out (**2-3 sets** **to be laminated and shared between participants**)
2. Audere assets middle 4 pictures (specific to Community Stakeholders)

- Instructions/suggestions to interviewer are in *italics*.

**Background**

1. Please tell me about a little bit about what you do and your role in the community.

**Priority Diseases**

1. Which health conditions in your community are cause for concern?

**Rapid diagnostic tests/home-based testing**

1. What do you understand by the term rapid diagnostic test?

*[****Interviewer,*** *If does not know, say these are tests that can give results quickly, for e.g., blood pressure cuff, diabetes test, pregnancy test]*

1. What do communities know about rapid diagnostic tests?
   - Can you tell me which ones they are familiar with?
   - Who uses rapid diagnostic tests?
   - Where are rapid tests used?
   - What are the benefits of using rapid tests?
   - What are the worries and concerns of using rapid self-tests?
2. How would the community feel if diagnostic tests were available to them to conduct the test themselves?
3. Where do you think the people in your community would want to do the rapid self-tests?
4. Which rapid test kits do you think should be prioritized for the people in your community, and why?
   1. What about for
      1. For HIV?
      2. For diabetes?
      3. For hypertension?
      4. For malaria?
      5. Pregnancy?
      6. STIs?
5. In order of priority, what are the top 3 rapid tests should be made available to communities from among those that you mentioned?
6. Which tests do you think the people in your community would be comfortable to self-test in their homes or venue of choice within the community, and why?

**Digital interventions**

11. How have communities reacted to digital interventions introduced in the country to improve care-seeking and access to healthcare? *[An example maybe SMS reminders/ broadcast messages]*

- - Benefits, challenges, concerns

[**Interviewer,** *please show the phone demo of the app*]

1. What do you think about the patient using an App like the one I have shown you, to be guided through self-testing and next steps, such as home care or getting advice from a healthcare provider via the App?
2. How comfortable do you think the people in your community would be with using the App? (*Culture, familiarity with technology, literacy, age, etc.)*

a. What would be the benefits of using the App?

b.What would be the concerns and difficulties of using the App?

1. What do you think about community members using an App to get a prescription?
   - Benefits
   - Challenges
   - Concerns
2. What do you think about community members using an App to order medication for home delivery?
   - Benefits
   - Challenges
   - Concerns

**Mobile Apps, Data and Systems *[potentially show prototype of proposed study app to facilitate questions]***

1. What do you think about individual community members having access to their medical records, for e.g., test results through this app?

[**Interviewer,** *please go to the middle 4 pictures in the Audere assets. Show the first 2 phots of the set for community stakeholders*]

1. Would people in your community be comfortable to use an App like the one we showed you to enter all their personal information for e.g., address, current symptoms, and test results, which a healthcare provider can then use to provide medical advice?

**Innovations [*Only applies to stakeholders who either influence/make policy or implement the policy as program*]**

***Interviewer, ask Policy makers* (political and traditional leaders):**

1. What are the processes for bringing in new rapid diagnostic tests and related digital health interventions, such as apps, into policy?
   1. What information do you need to make these decisions?

***Interviewer, ask Program implementers*** *(NGO, CBOs)*

1. What would the processes for implementing policy on new rapid diagnostic tests and related digital health interventions?
   1. What challenges do you face in supporting implementation of new policy?
   2. What solutions have worked in countering these challenges in the past?
