# Supplementary material for: Perspectives on App-Assisted Self-Testing Using Rapid Diagnostic Tests Among Community Members, Health Care Providers, and Public Health Leaders in Kenya, South Africa, and Zambia: Qualitative Study
Source: J Med Internet Res. 2025 Nov 26;27:e70273. doi: 10.2196/70273 (PMC12696451; doi:10.2196/70273)
Supplement: Multimedia Appendix 2 [file jmir_v27i1e70273_app2.zip › Multimedia 2 DASH interview guides/Training_UW_DASH_QualStudyAssets_FromAudere_Mar30_2023.pdf]

DASH Study Training

Qualitative Survey Prototype & Dashboard Assets

audere

Introducing Audere

Digital solution provider

About Audere

- We are a team of innovators accelerating high impact and quality healthcare delivery in LMIC
- 501(c)(3) public charity founded through generous funding from the Bill & Melinda Gates Foundation
- Focused on delivering solutions that advance health equity by revolutionizing the detection and treatment of diseases, such as malaria, COVID-19, HIV and TB
- HealthPulse and TestNow solutions aid in accurate and appropriate outcomes for rapid diagnostic tests

> "Provide innovative, scalable, interconnected digital health solutions which accelerate accessibility of care for more people globally
>
> –Audere Mission

Use Cases enabled by HealthPulse AI solutions

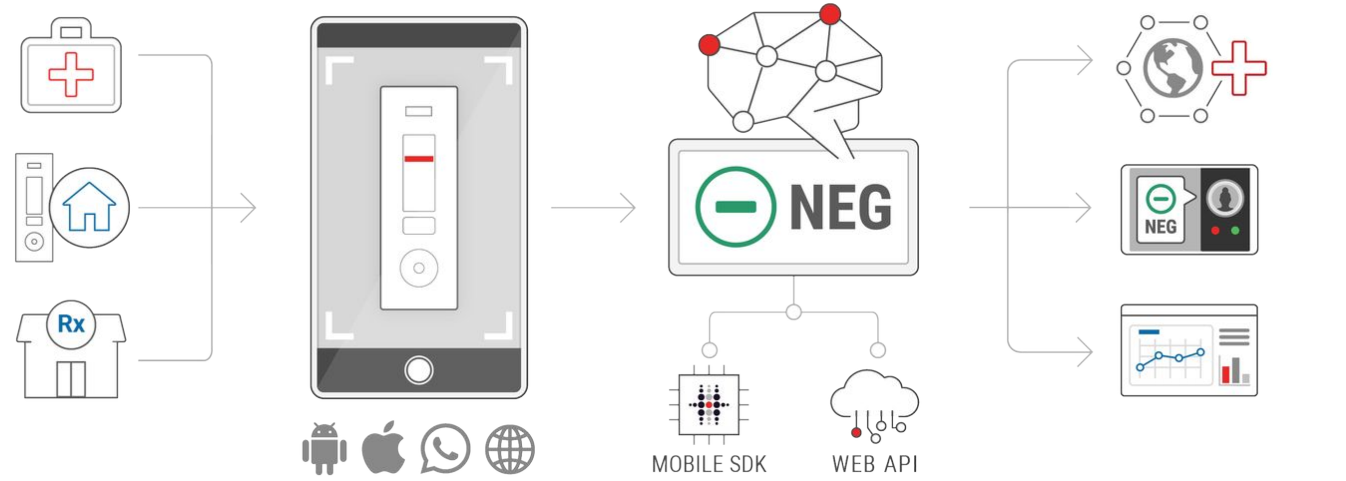

Any care location

- Clinicians & CHWs
- Self-tests
- Pharmacies

Any digital tool

- Chatbots
- Native apps
- Mobile web

HealthPulse AI

- Image quality analysis
- Result interpretation
- Anomaly detection

Actionable insights

- Public health surveillance
- Telehealth
- Quality assurance

Self tester in-app flow example

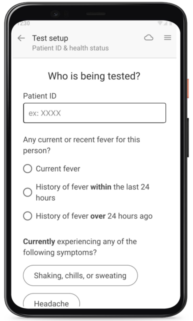

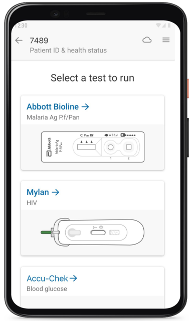

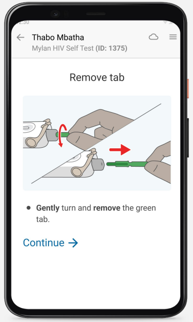

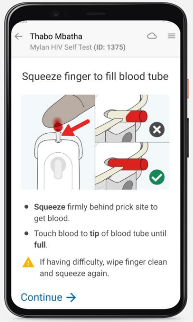

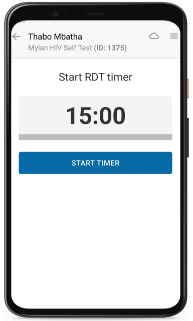

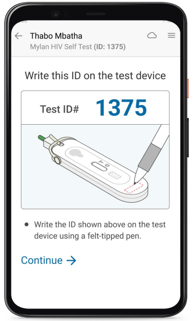

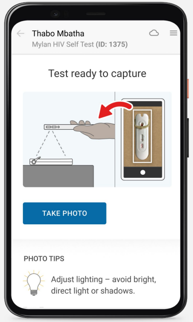

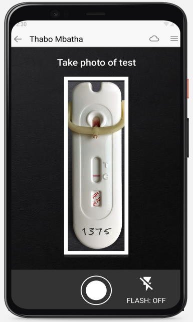

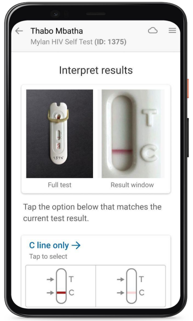

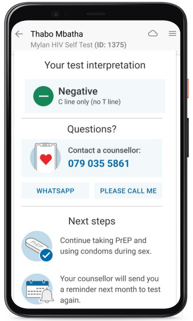

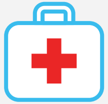

Patients needing care are directed to local CHWs (via WhatsApp or other channels)

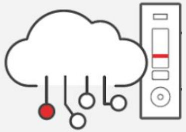

AI used for image quality assurance and interpretation verification

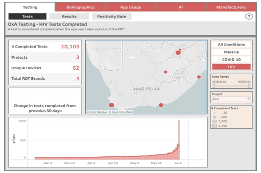

AI powered interpretation checks enable CHWs to identify care needs

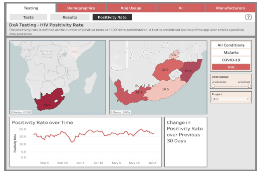

Program stakeholders monitor overall disease & testing trends

Self tester fills out patient info & selects a test to run → Test administration instructions → Processing timer & Patient-test identifiers → Photo capture → Guided Interpretation → Connection to care → Data surveillance & insights

TestNow App Access

Prototype for use in the DASH study

Connecting to ProtoPie prototype

- Search for ProtoPie in the Google Play Store or Apple App Store
- Install ProtoPie
- Open ProtoPie and go to the **Studio** page.
  - **URL**: Tap the purple on-screen dropdown and select **Cloud Pie URL.** Copy the below URL into the field: https://cloud.protopie.io/p/2e31c712d6224a98976bb8ab
  - **Use QR code:** Use the QR code at the bottom of this screen. May be a little tricky to scan from a screen. May work better from a printed sheet.
- Tap the **Open this Pie** dialog at the bottom of the screen
- The prototype will open full screen.
  - ***TIP***: In order to get back out to the menu, double tap on the screen quickly using **two** fingers.
- From the menu, tap **Make Available Offline**, which will switch to **Available Offline** with a green arrow.
- **Troubleshooting**: If ProtoPie has issues (such as freezing, not scanning) force ProtoPie to close and reopen.

QR code to access prototype from within the ProtoPie app

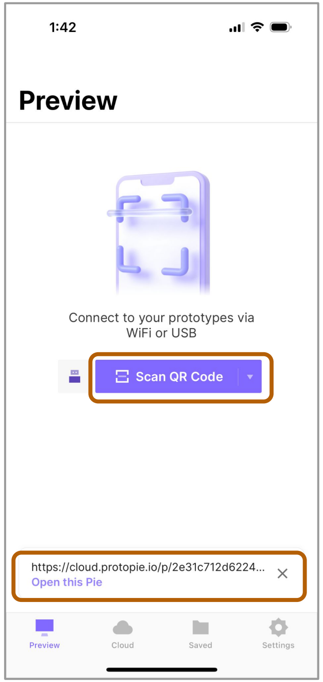

Scan **QR** code & click **Open this Pie**

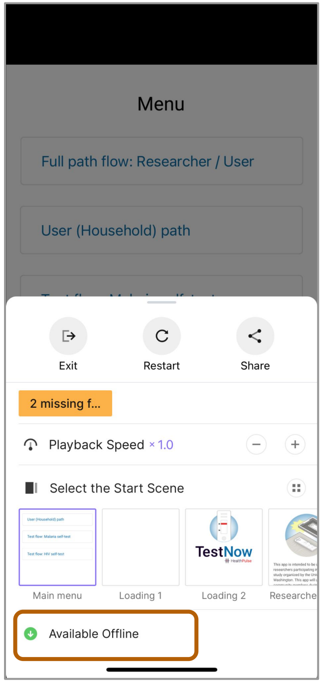

Select **Available Offline**

Using the Prototype

- **Who is being tested** page:
  - Enter any patient ID. It will be shown on the next pages in the header
  - Other fields are not tappable on this page, go to the bottom and click Submit.
- **Select a test to run** page: Select the malaria or HIV test.
- **Timer** page: Tap on timer to run down the clock
- **Interpretation** page: Select positive, negative, or invalid
- **Test results** page: Final page in a flow. Tap **Run a different test** to go back to the **Select a test to run page** (if you want to first show malaria and then HIV for instance)
- If anything goes awry - 2 finger tap to get to the main menu of ProtoPie. Then tap restart to get to the main menu of the prototype.
- **Back button:** The back button in the top left is functional through most of the flow.

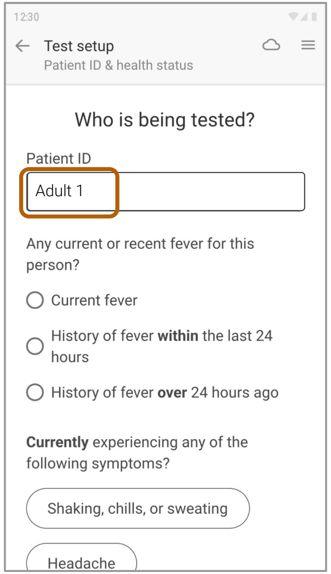

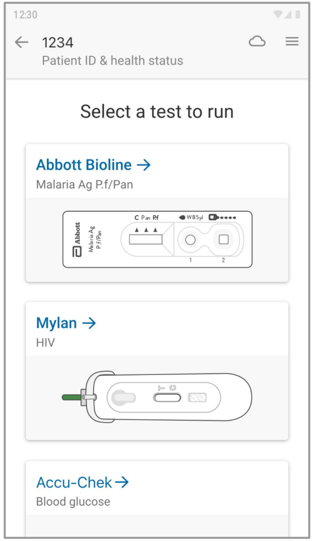

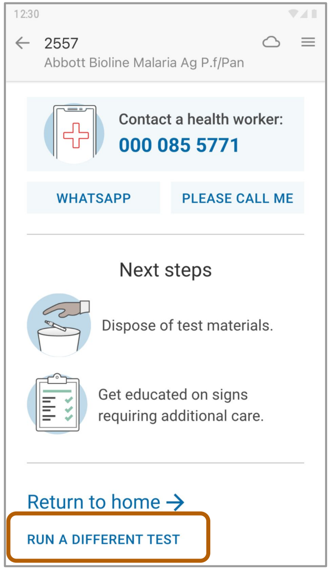

Returning to the ProtoPie prototype

After setting the prototype as “Available Offline,” you can reopen it by:

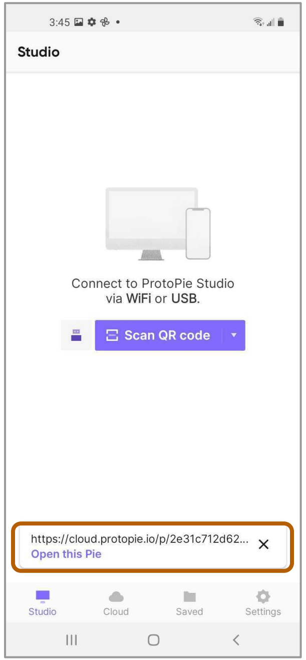

Use a link on the **Studio** page (if showing)

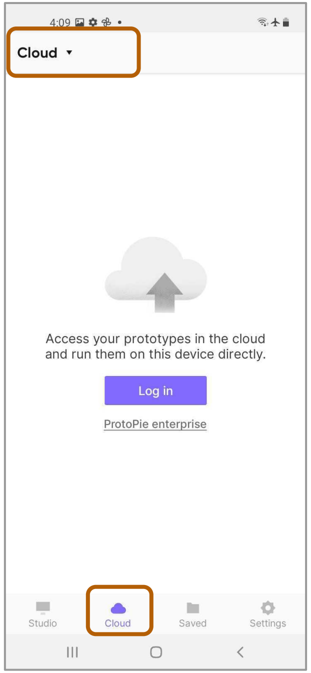

Tap **Cloud** on the bottom and then on the top

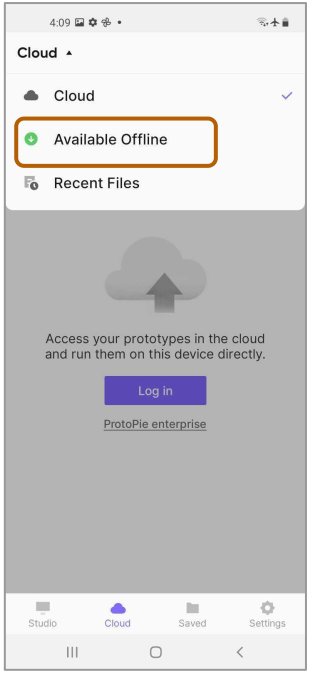

Select **Available Offline**

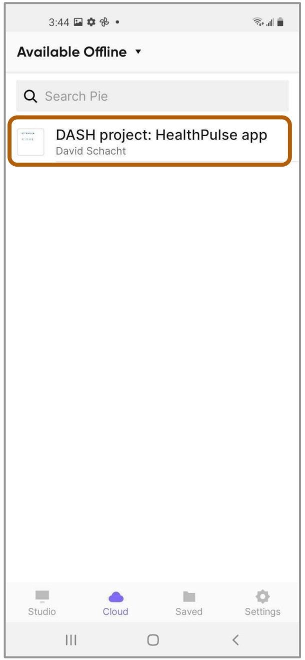

Select the **DASH** project

Running through the TestNow prototype

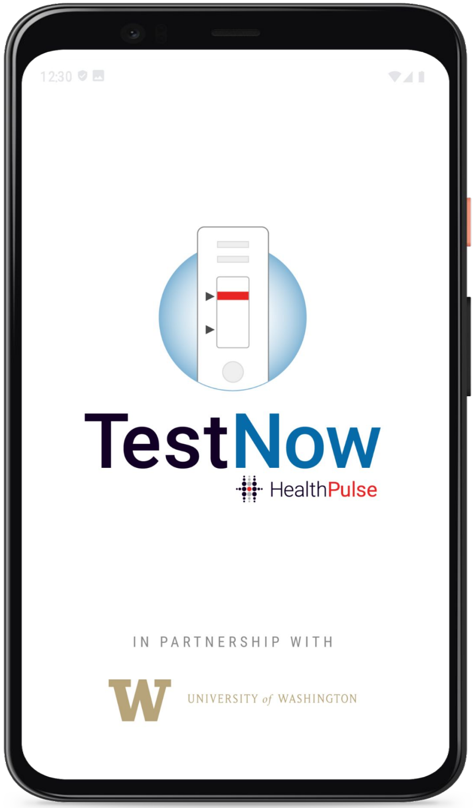

Let's take the
TestNow prototype
for a spin

Dashboard examples for use in interviews

→ **Policy makers**
Community stakeholders
Provider stakeholders

Stakeholders could view overall population stats and cross-disease data at the country, county, and community level

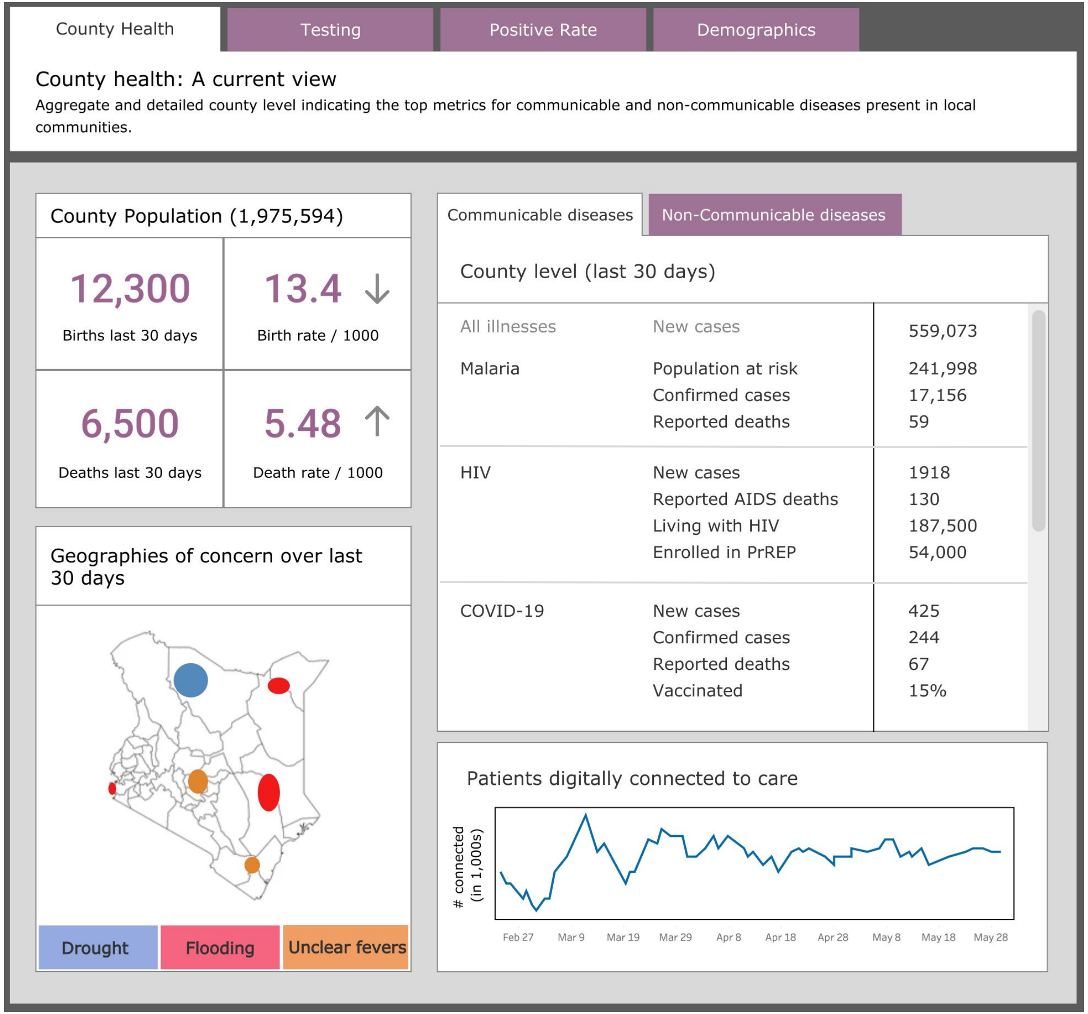

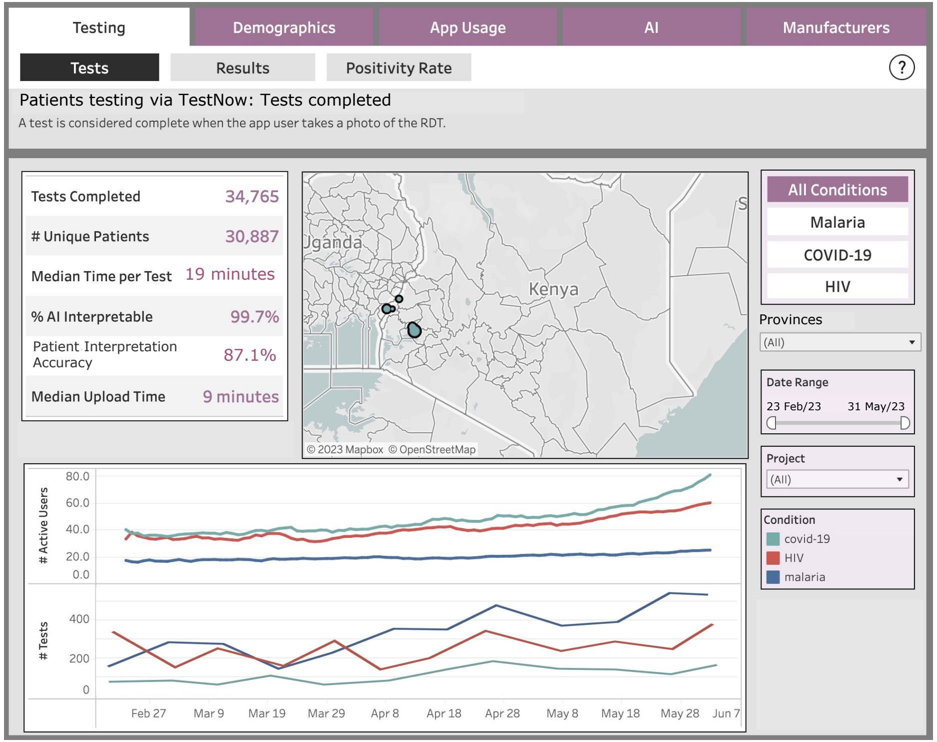

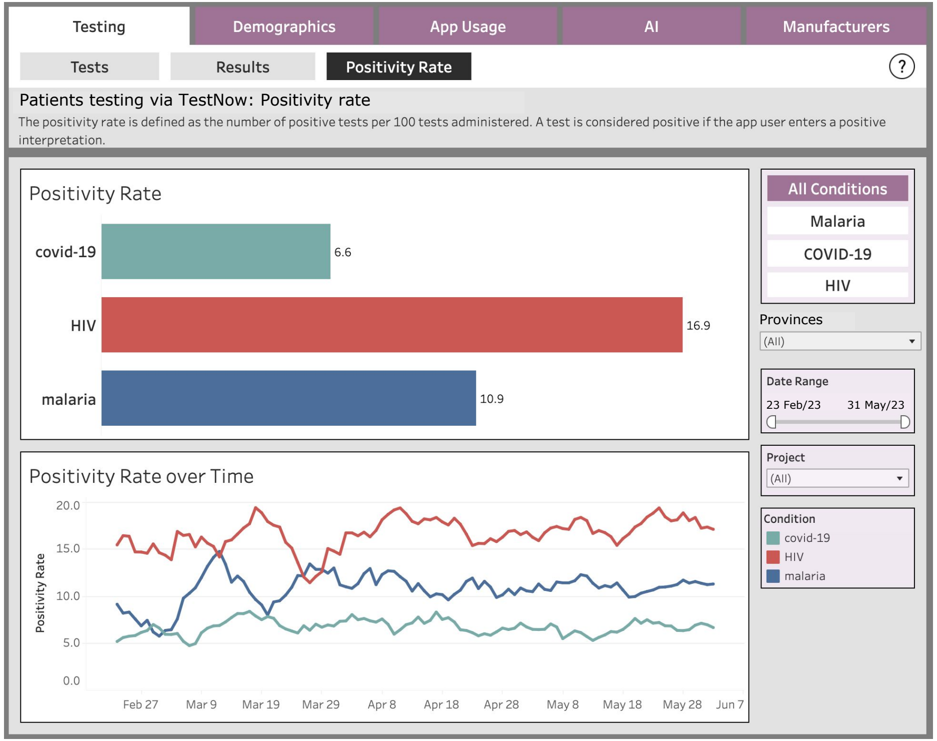

Auto-confirmation of the patient’s RDT interpretation based on the photo

Geospatial mapping of community rapid diagnostic testing

Understand trends in positivity rate at country and county levels

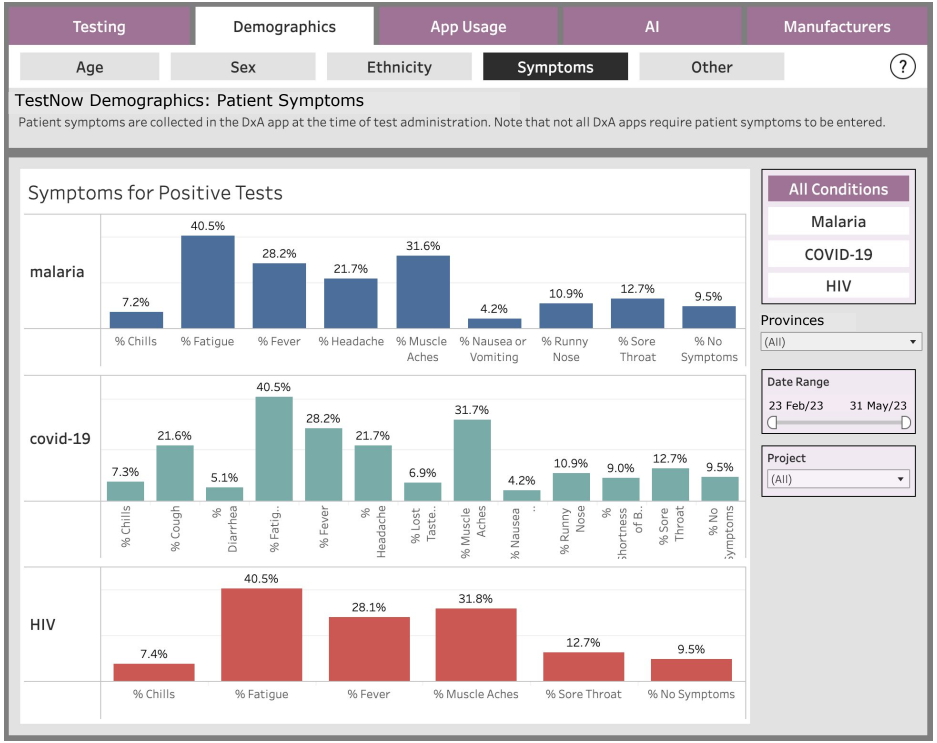

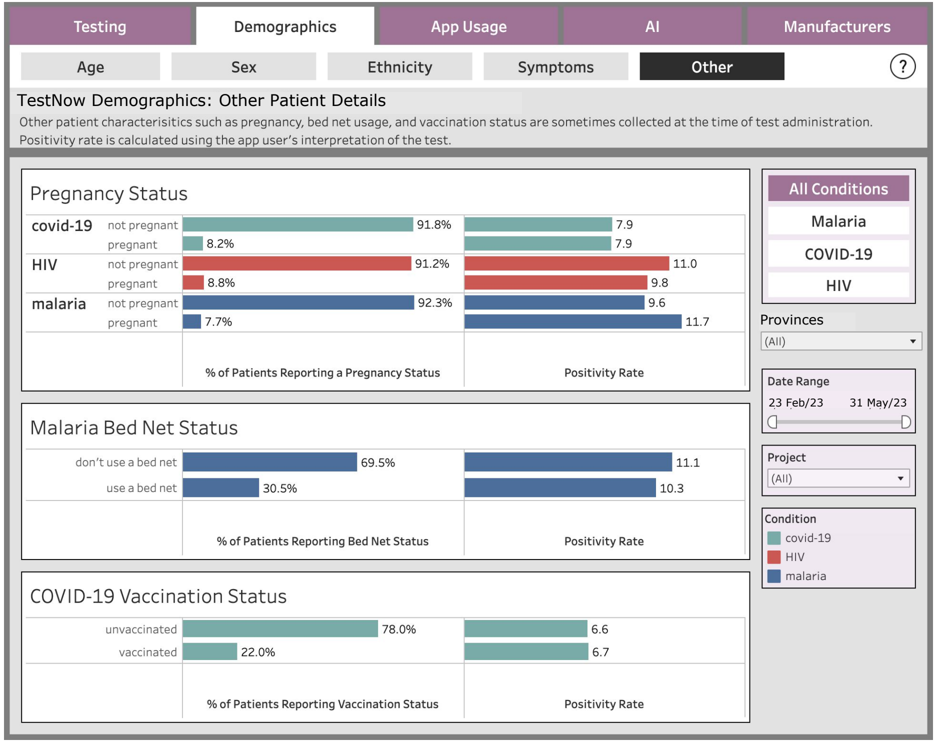

Collect current symptoms at the time of testing & review across illnesses

Use self-testing apps to collect additional data of interest

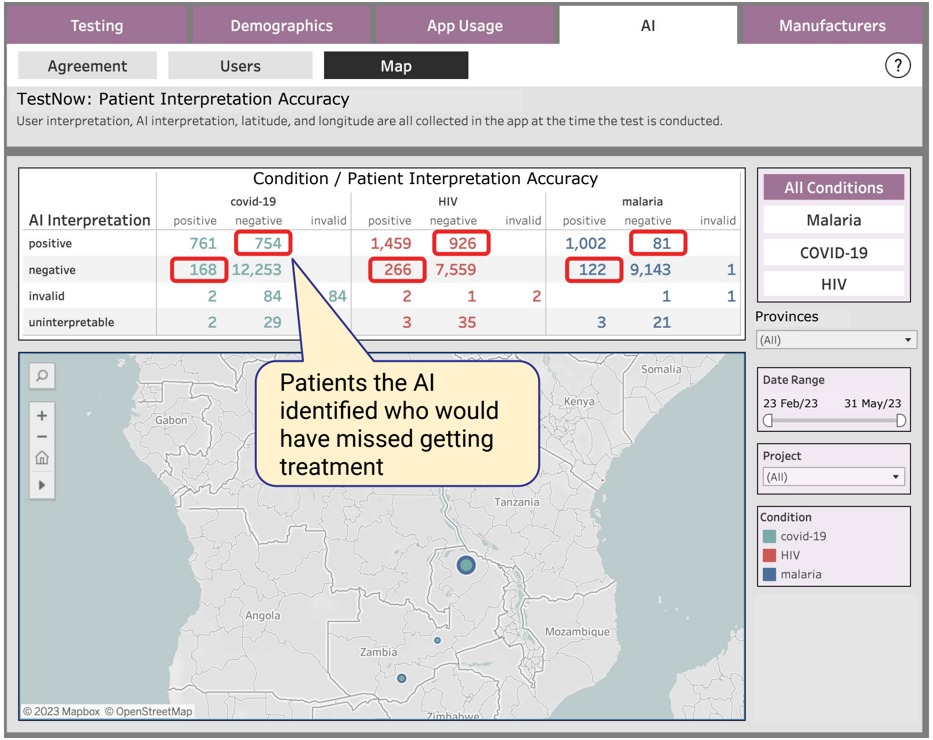

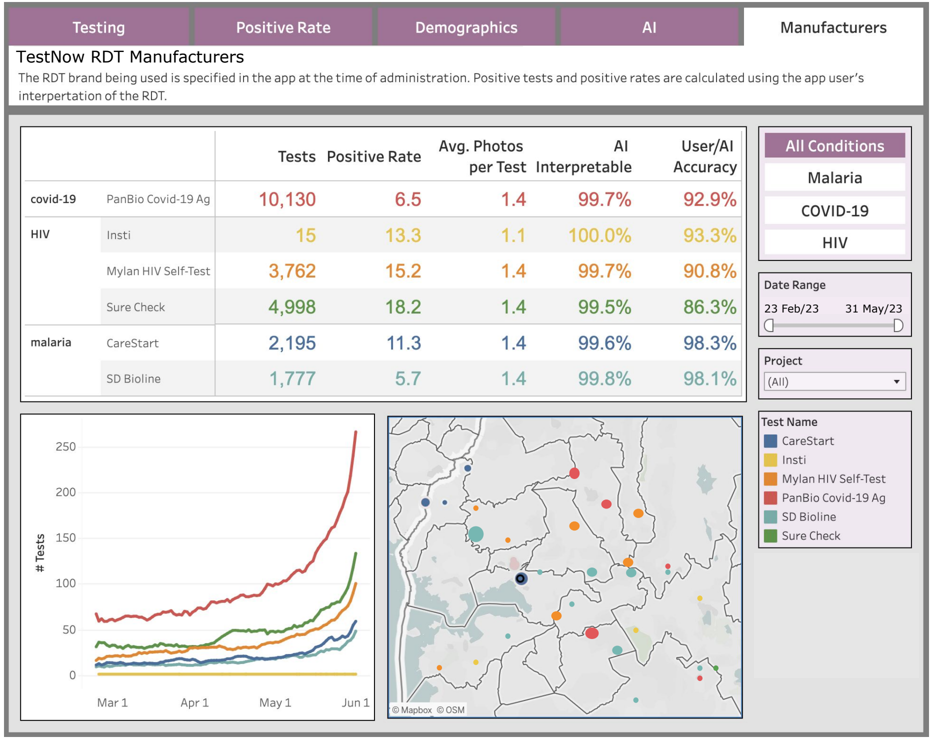

Artificial intelligence (AI) identifies results which were incorrectly interpreted by self-testers

Visibility into which test brands are preferred/available in communities

Dashboard Examples

Policy makers
→ **Community stakeholders**
Provider stakeholders

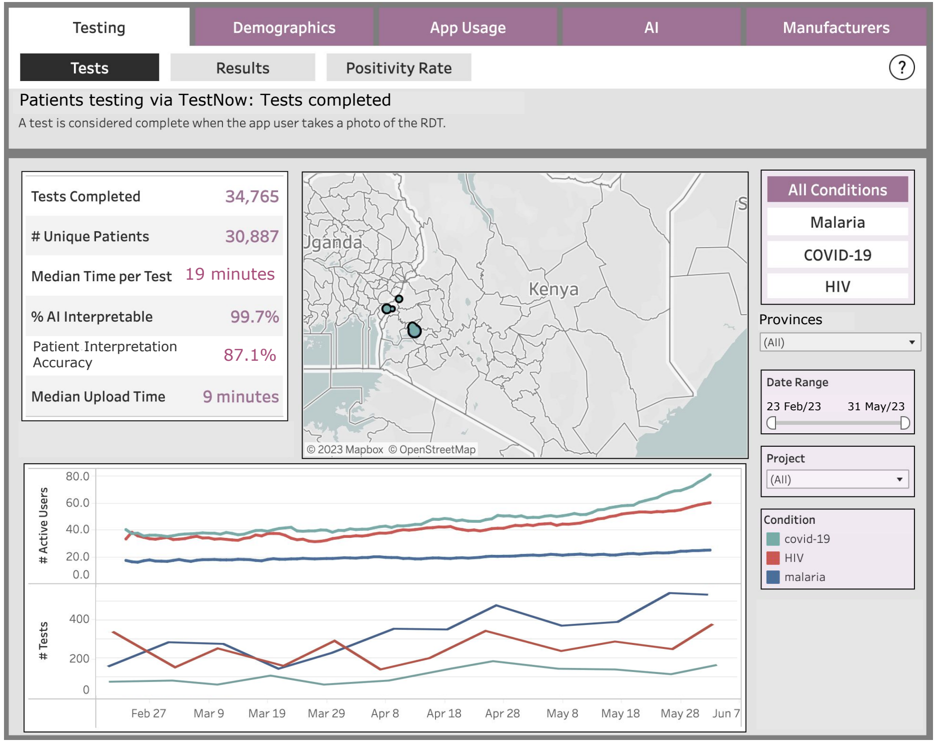

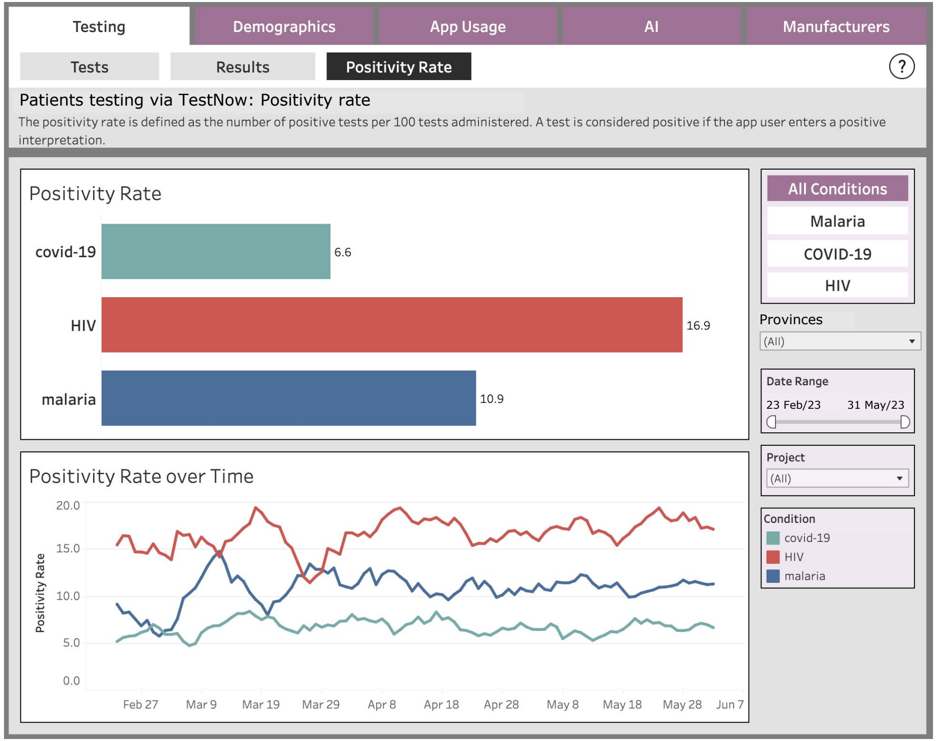

Auto-confirmation of the patient's RDT interpretation based on the photo

Geospatial mapping of community rapid diagnostic testing

Understand trends in positivity rate at country and county levels

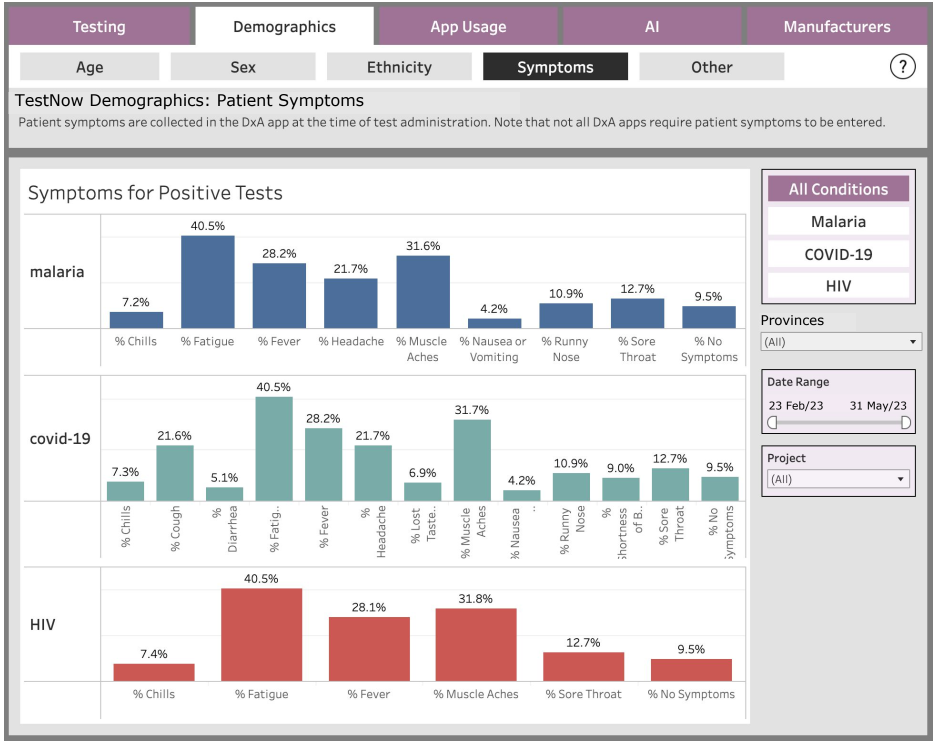

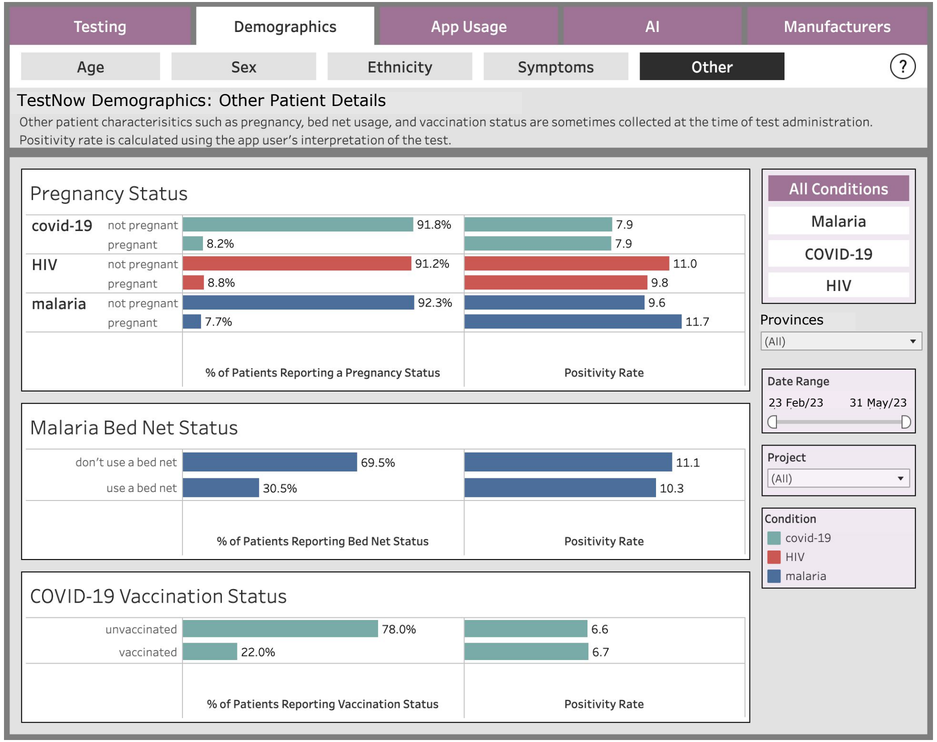

Collect current symptoms at the time of testing & review across illnesses

Use self-testing apps to collect additional data of interest

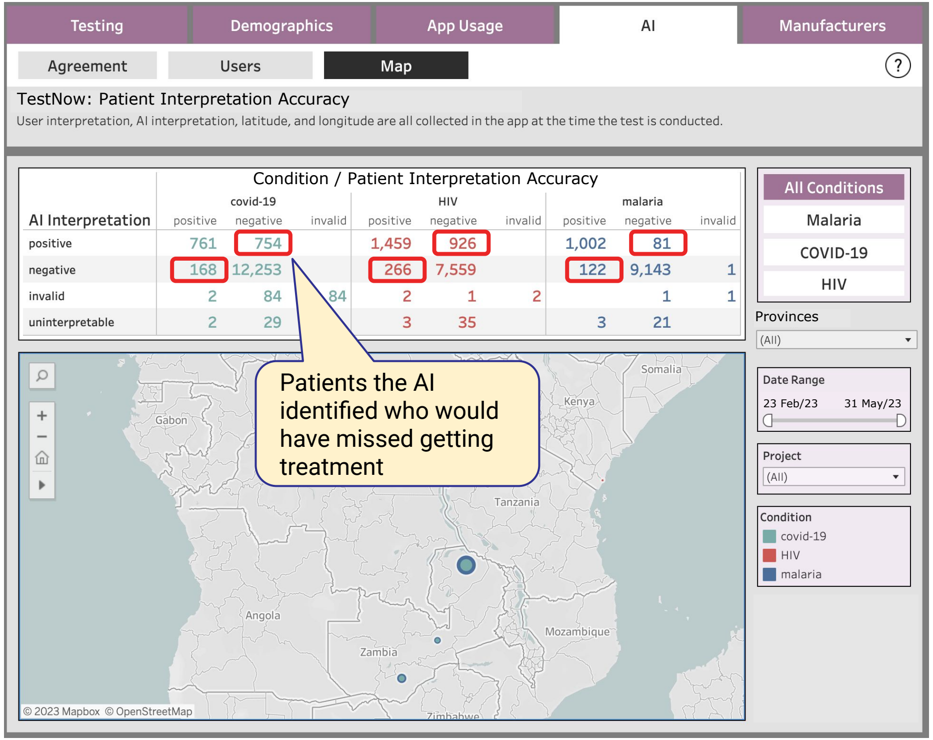

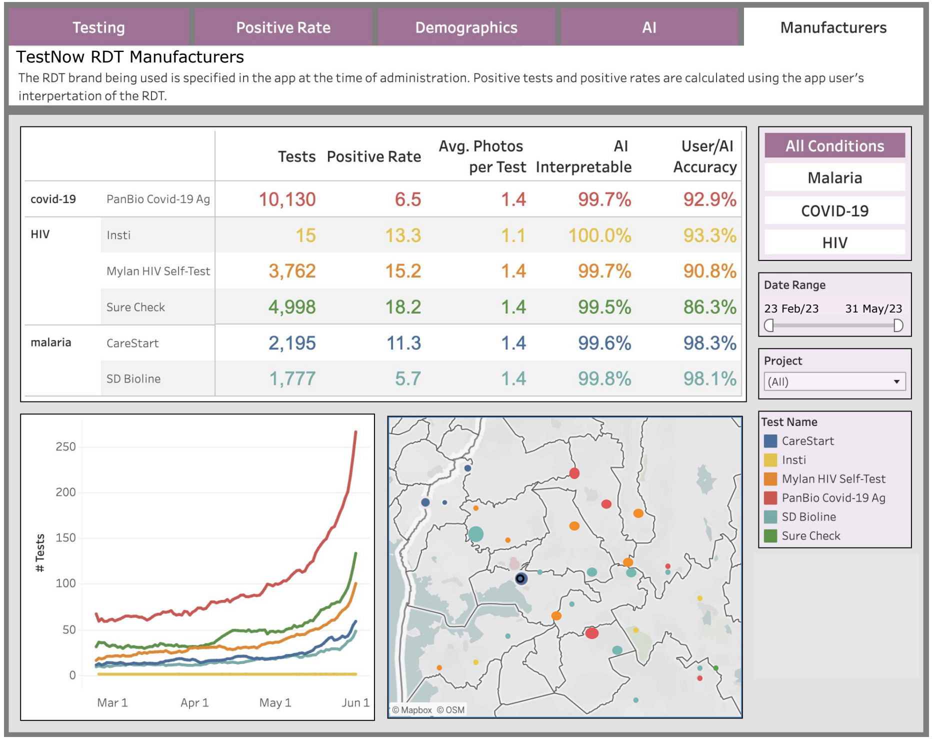

Artificial intelligence (AI) identifies results which were <u>incorrectly</u> interpreted by self-testers

Visibility into which test brands are preferred/available in communities

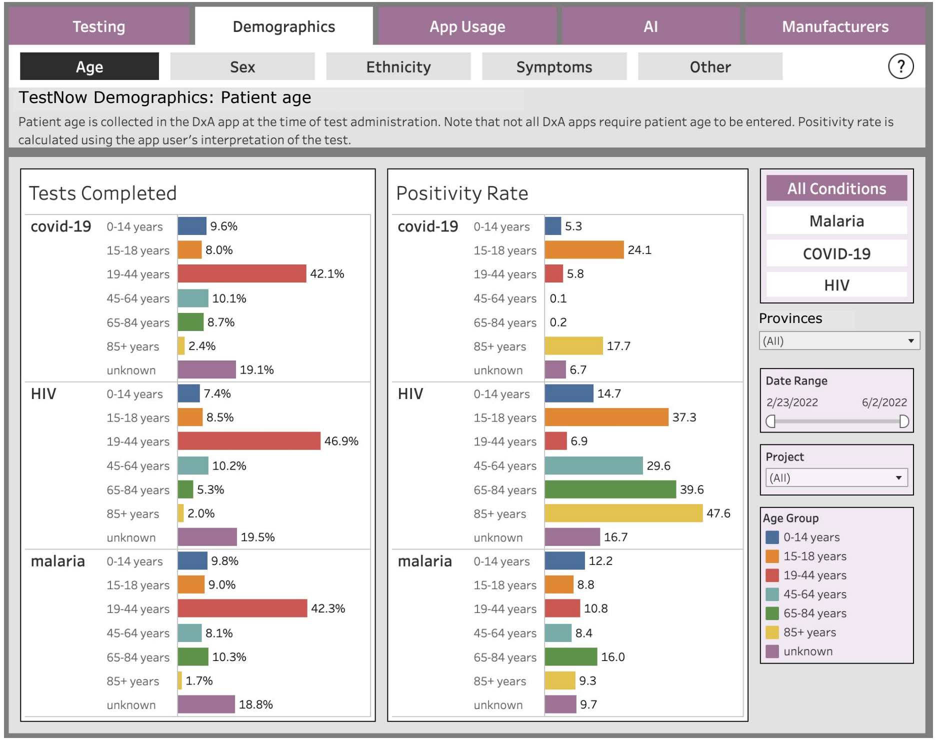

Testing & positivity rate breakdown across diseases & age

Dashboard Examples

Policy makers
Community stakeholders
→ **Provider stakeholders**

Providers have a view into the health of their community:

- **where** people are testing
- **when** they are testing
- **what** they are testing for

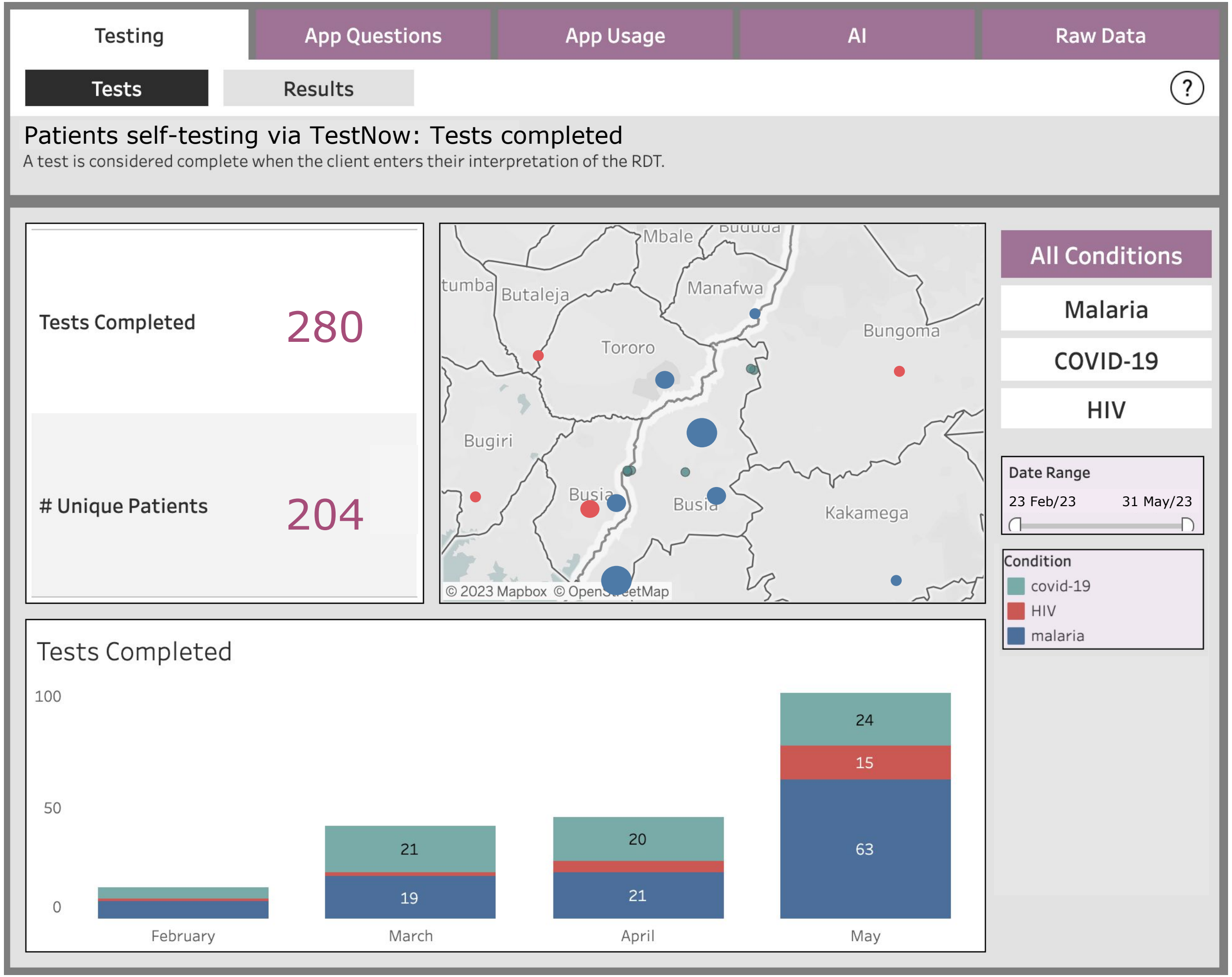

Alerted to patients who may need support or follow-up

Testing | App Questions | **App Usage** | AI | Raw Data

Retention | Devices | App Activity | **Patient List** | ?

Patients using TestNow to administer RDTs: Patient List

A test is considered complete if the client enters their interpretation of the RDT. Client/AI Agreement is calculated as the number of tests where the client interpretation and AI interpretation match divided by the number of tests where both provided an interpretation.

Patient interpretation accuracy	87.1%
Median time per test	20 min
Testing protocol adherence rate	93.4%
Test completion rate	89.2%
Tests taking excess time to complete	8.0%

PrEP Test Compliance

Patient ID	February	March	April	Status
6	✓			Test Missing
698	✓	✓	✓	
704	✓	✓		Test Missing
715		✓	✓	
737	✓	✓		Test Missing
747	✓	✓	✓	
768	✓	✓	✓	
1279	✓	✓	✓	
1283	✓	✓	✓	

Start Dt

23 Feb/23 31 May/23

Search for patient

Condition
- covid-19
- HIV
- malaria

Patients to Review

Patient ID	Device	Last test run	Avg time per test (mins)	Alert	Patient interpretation accuracy
6	itel A16 Plus	HIV	22	Missing HIV test	100.0%
698	TECNO S6S	malaria	26	Test misinterpreted	85.0%
704	TECNO S6S	HIV	27	Missing HIV test	100.0%
715	Nokia 2.3	COVID-19	18	Test misinterpreted	78.0%
737	SM-A013G	malaria	22	Missing HIV test	100.0%
747	TECNO LA7	HIV	51	Long test time	100.0%
768	NEON_RAY	COVID-19	21	Missing HIV test	100.0%
1279	itel W5002	malaria	23	Test misinterpreted	56.0%
[illegible]	[illegible]	[illegible]	[illegible]	[illegible]	[illegible]

Sample clinical decision support app for providers

Alerted to review self-test results if a positive is identified by the patient or the system

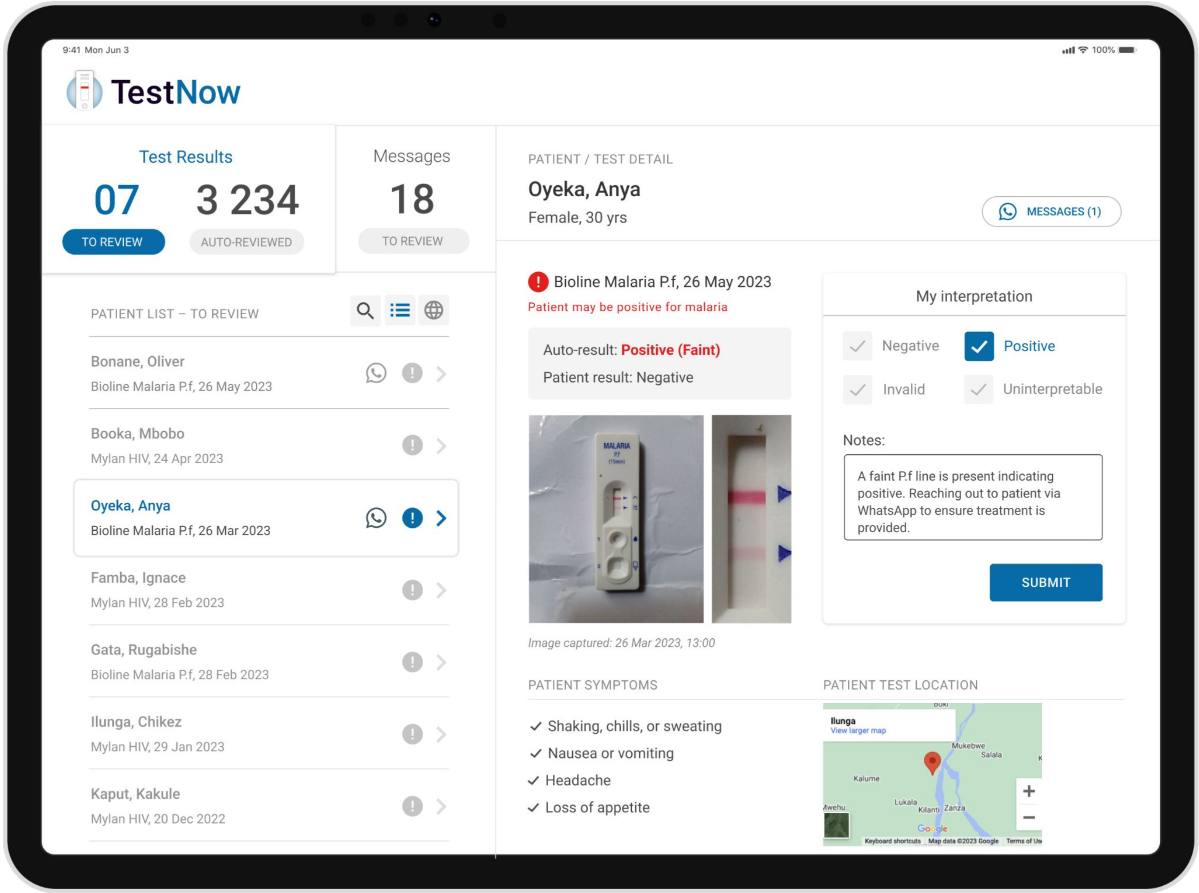

Provider interprets image

Sample clinical decision support app for providers

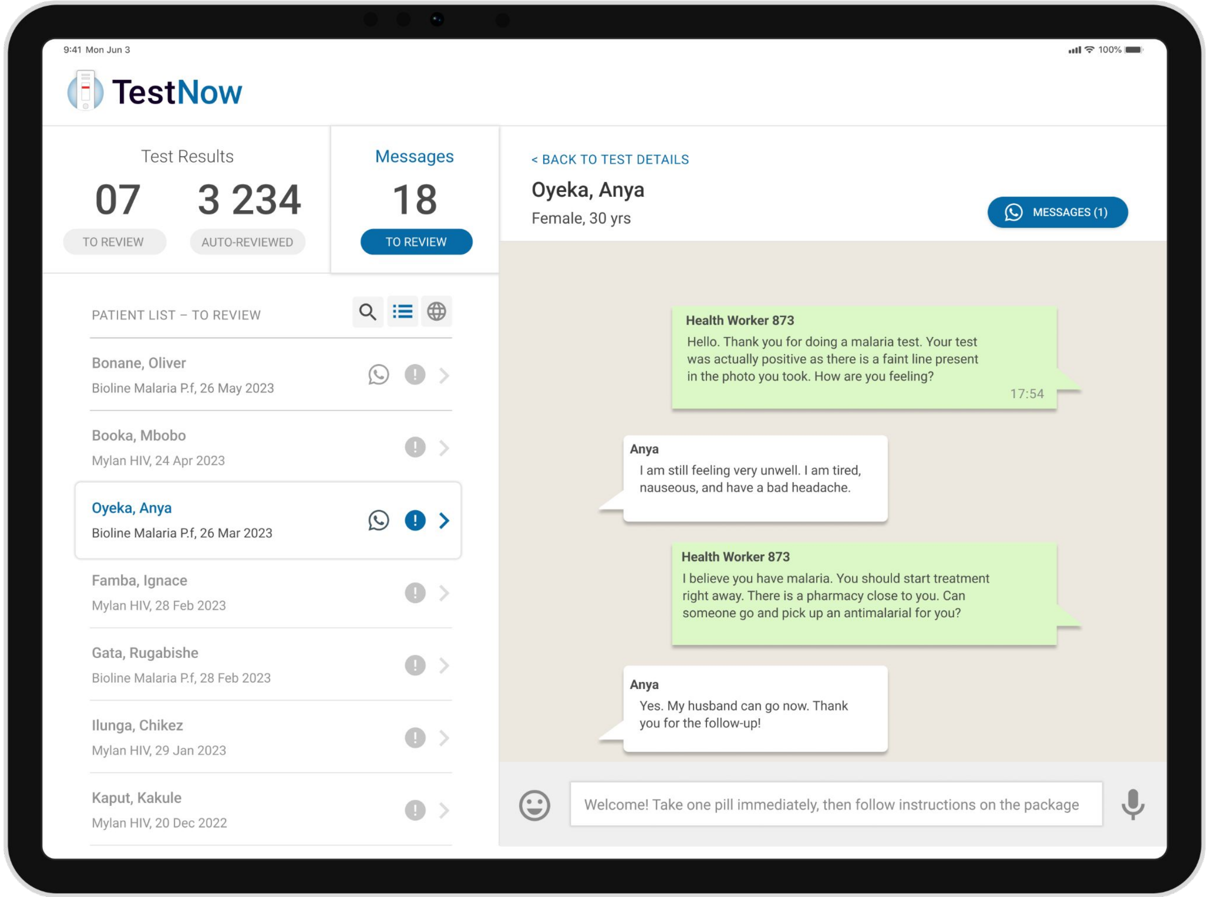

Chat with patients via WhatsApp to provide care guidance

HealthPulse
