# Supplementary material for: Perspectives on App-Assisted Self-Testing Using Rapid Diagnostic Tests Among Community Members, Health Care Providers, and Public Health Leaders in Kenya, South Africa, and Zambia: Qualitative Study
Source: J Med Internet Res. 2025 Nov 26;27:e70273. doi: 10.2196/70273 (PMC12696451; doi:10.2196/70273)
Supplement: Multimedia Appendix 2 [file jmir_v27i1e70273_app2.zip › Multimedia 2 DASH interview guides/Tests and Screening activity cards.pptx]

## Slide 1
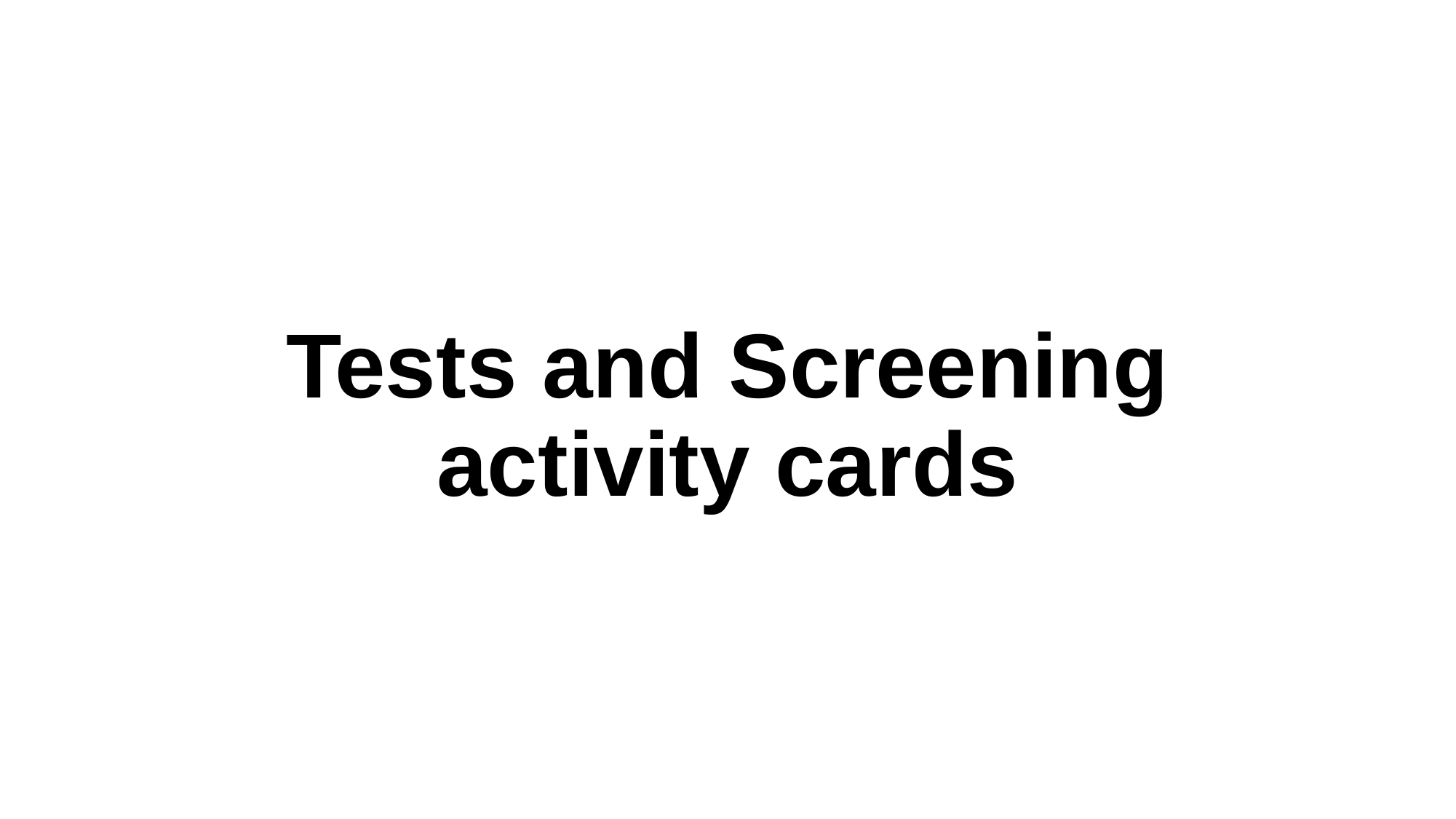

# Tests and Screening activity cards

## Slide 2
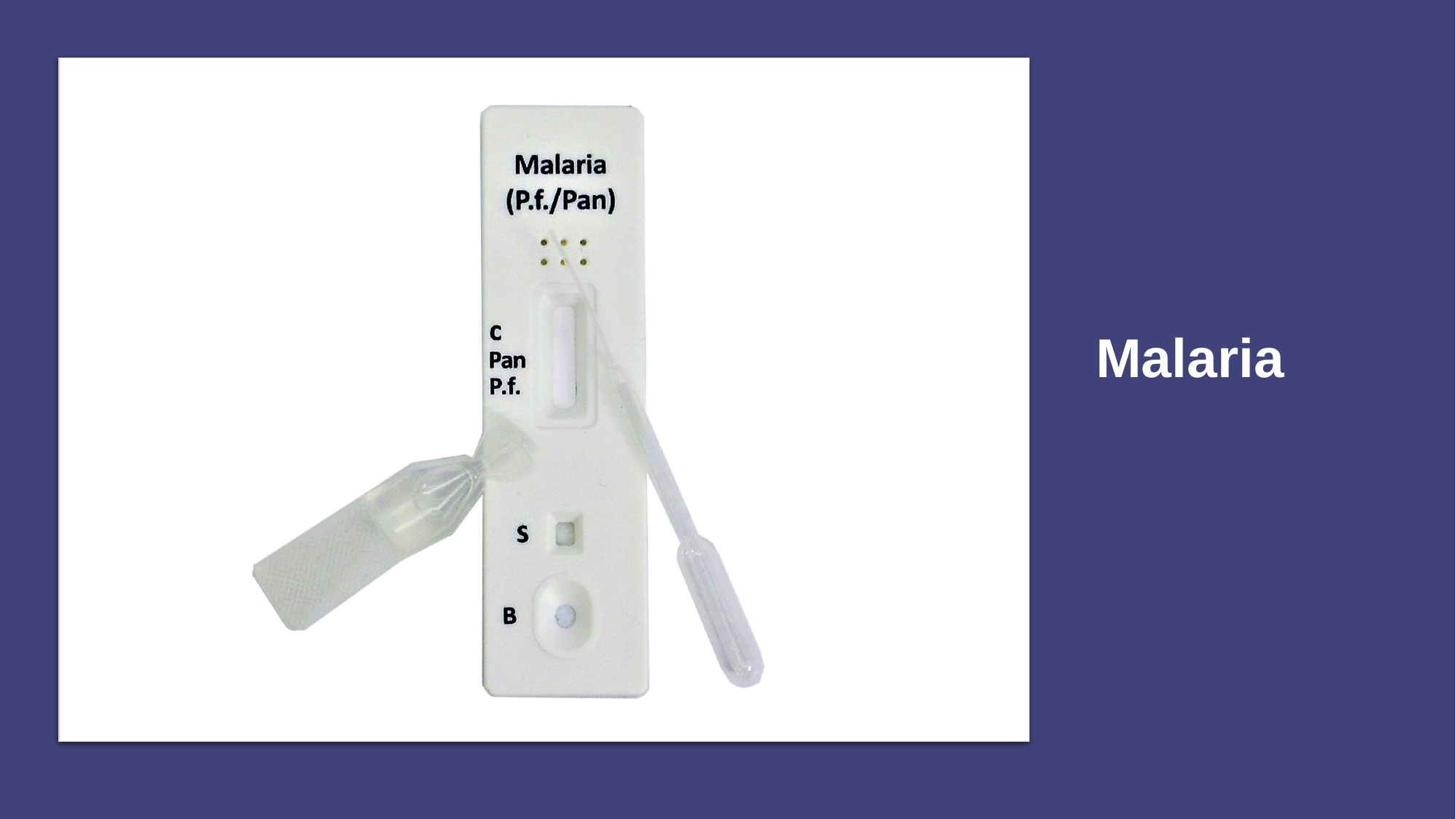

# Malaria

## Slide 3
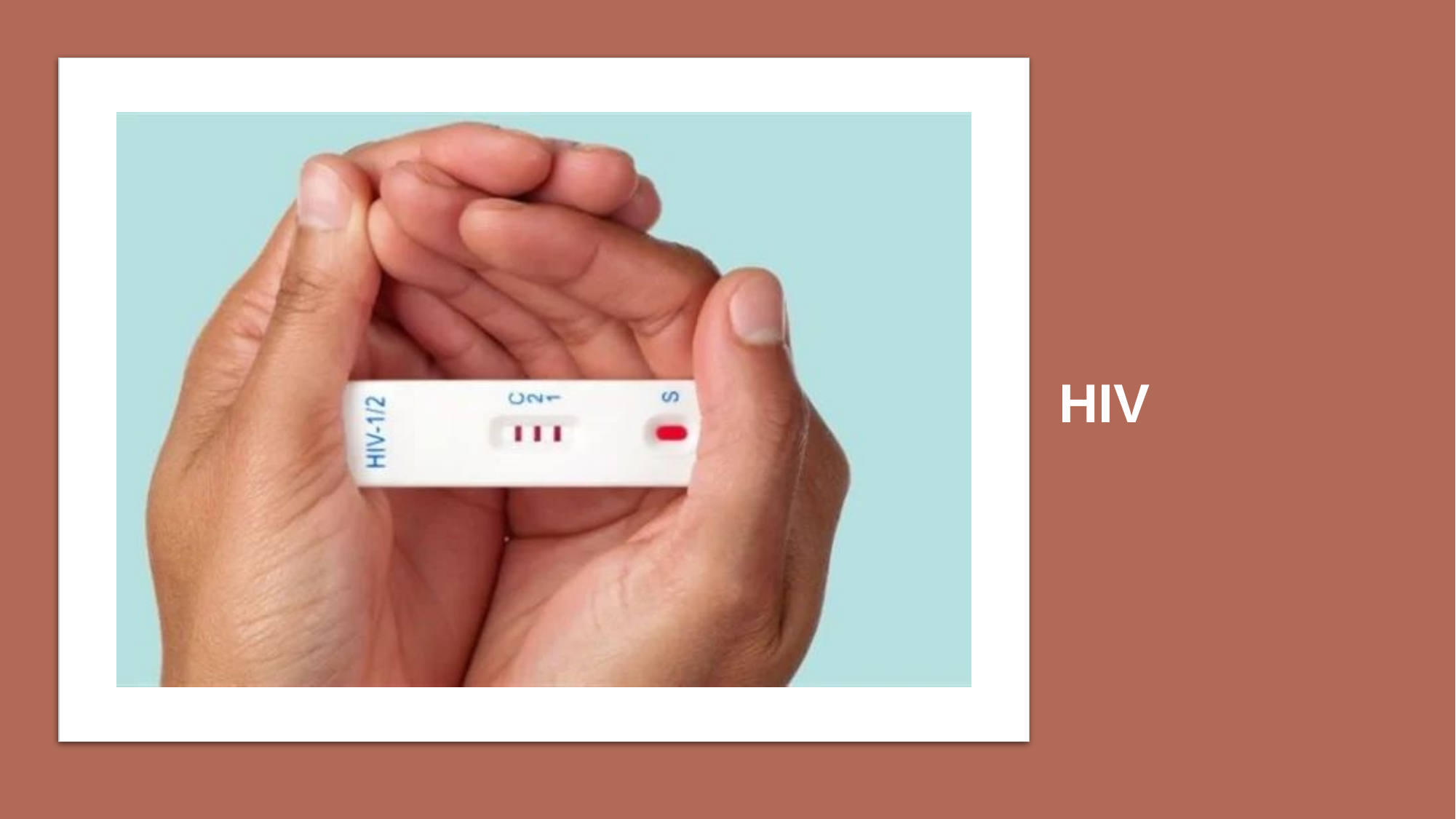

# HIV

## Slide 4
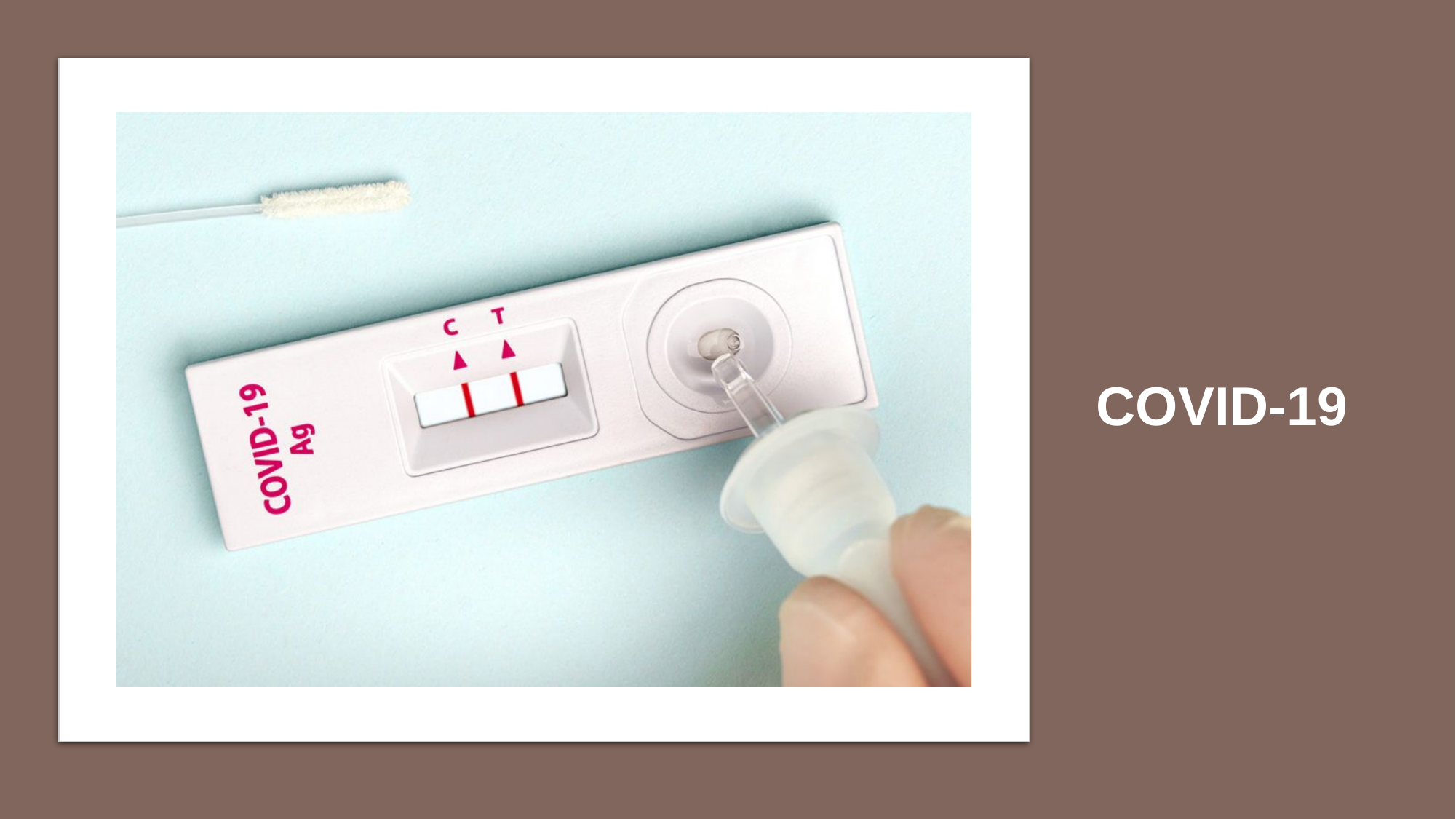

# COVID-19

## Slide 5
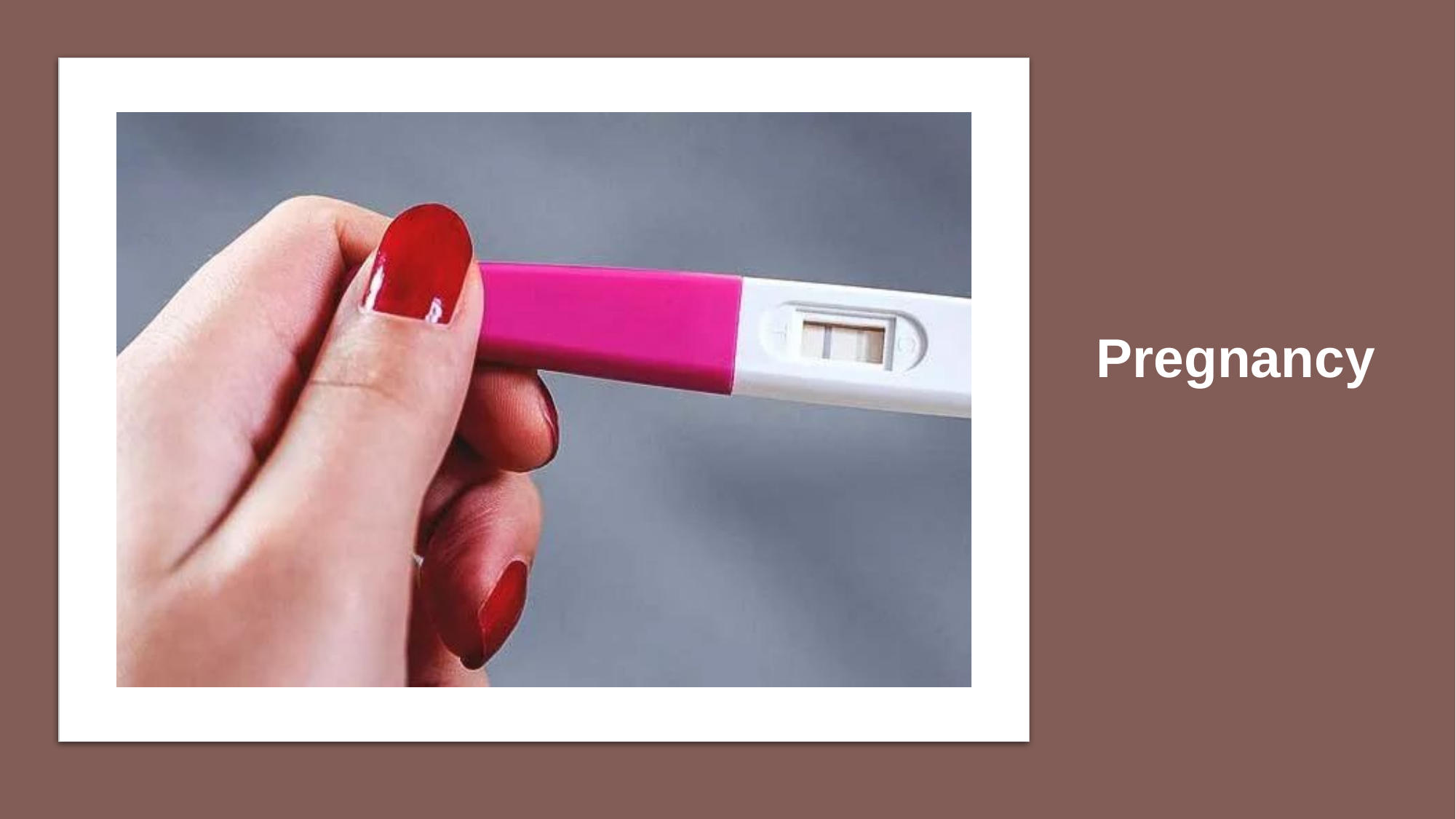

# Pregnancy

## Slide 6
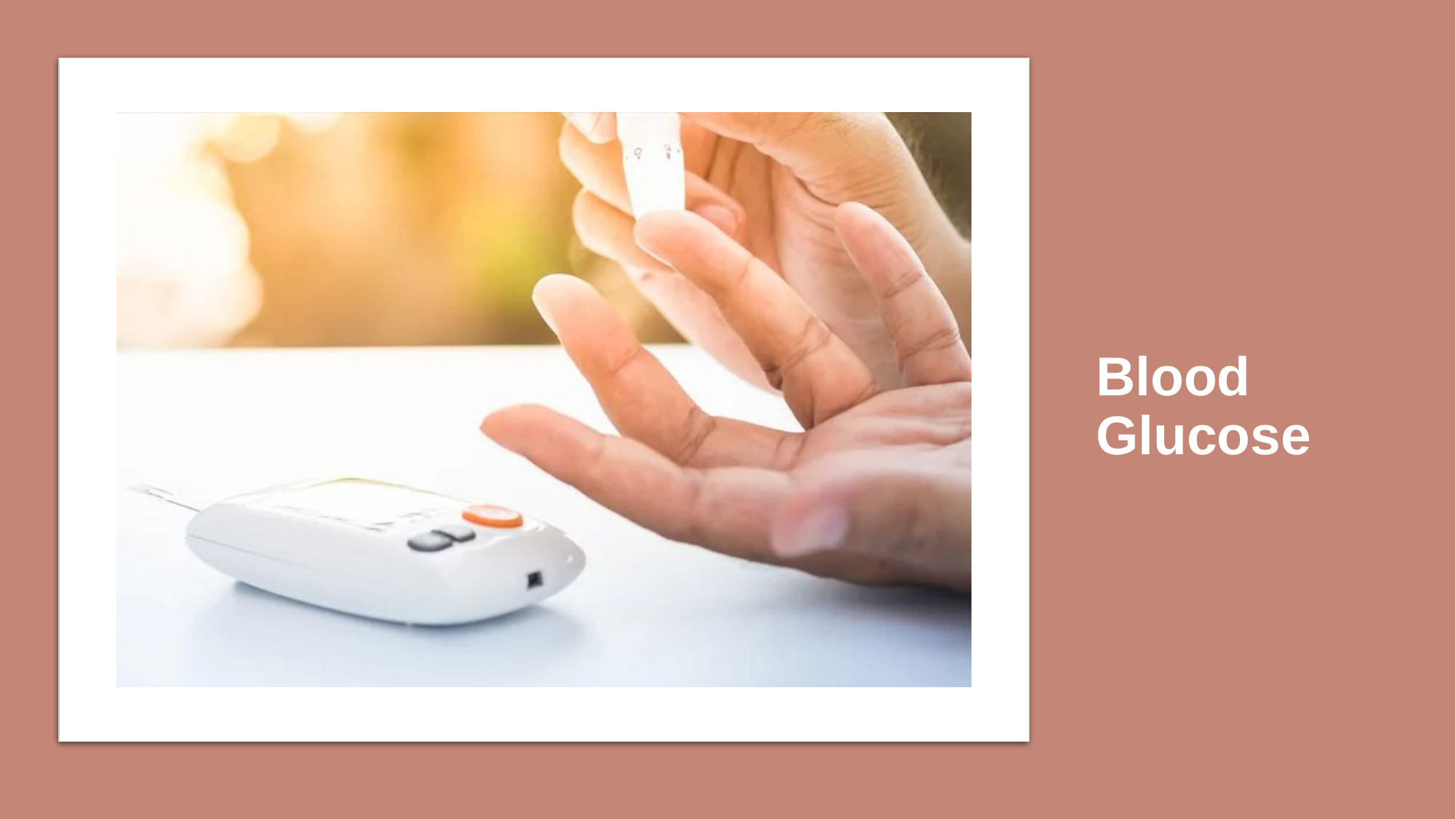

# Blood Glucose

## Slide 7
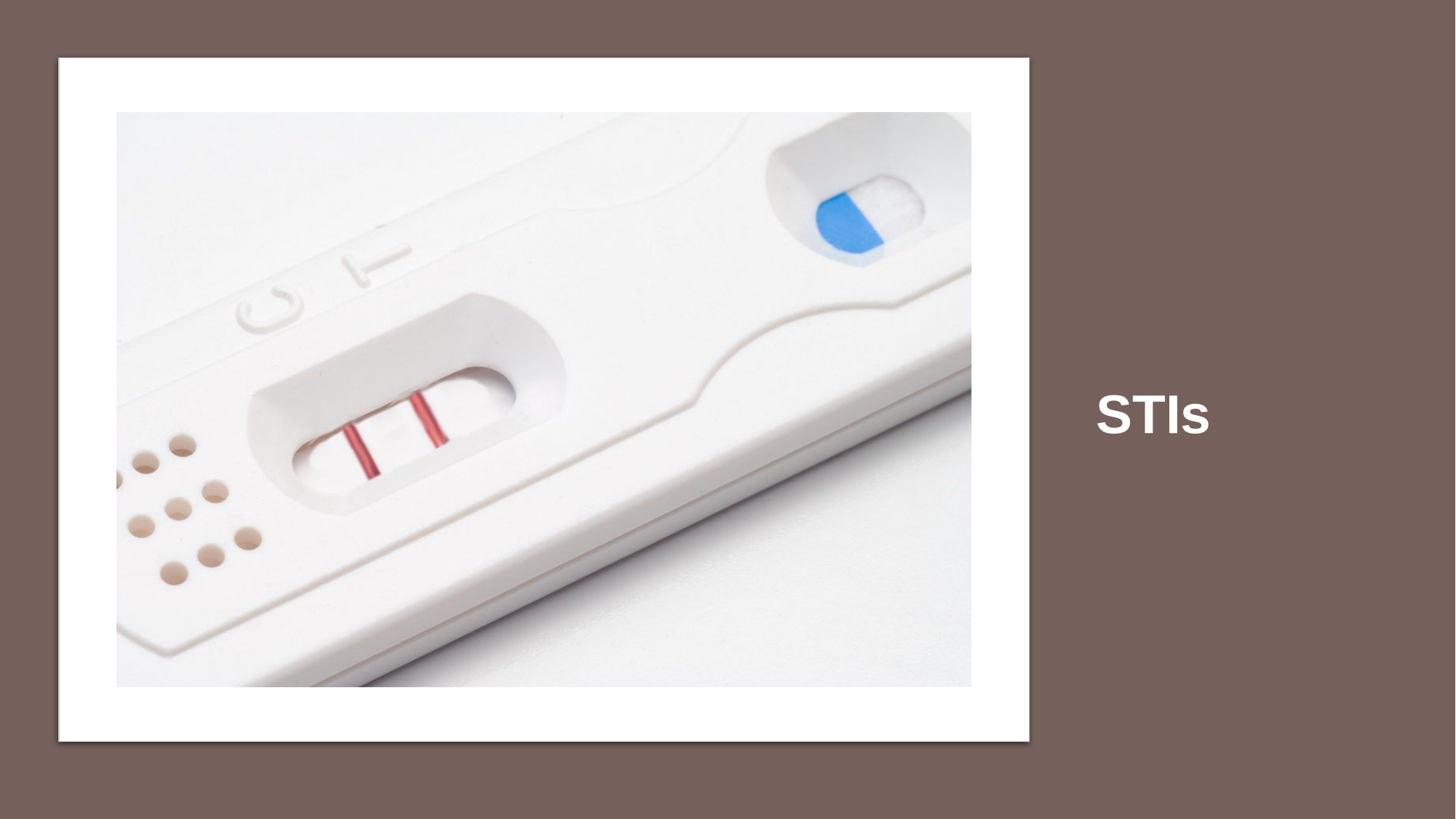

# STIs

## Slide 8
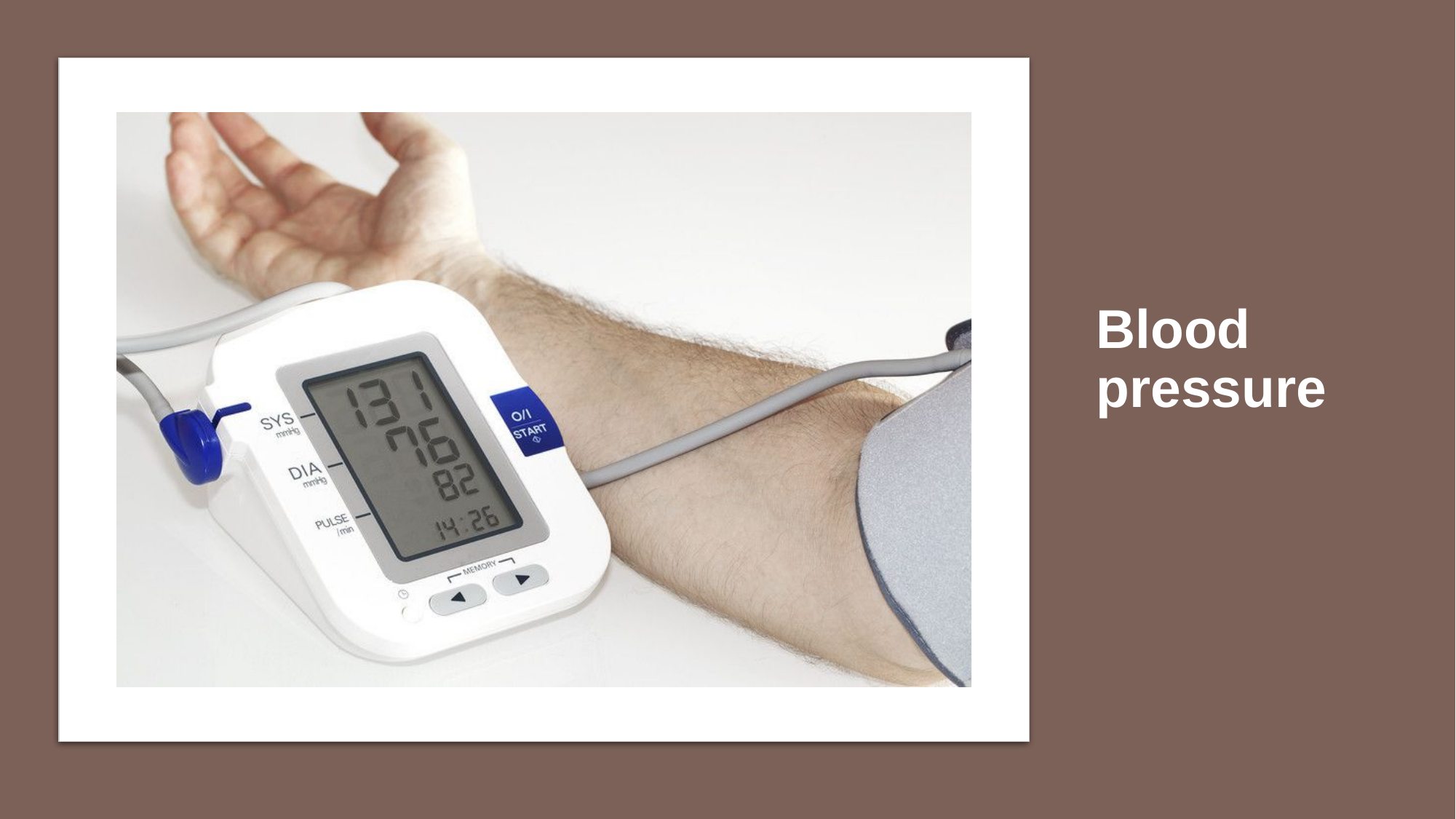

# Blood pressure

## Slide 9
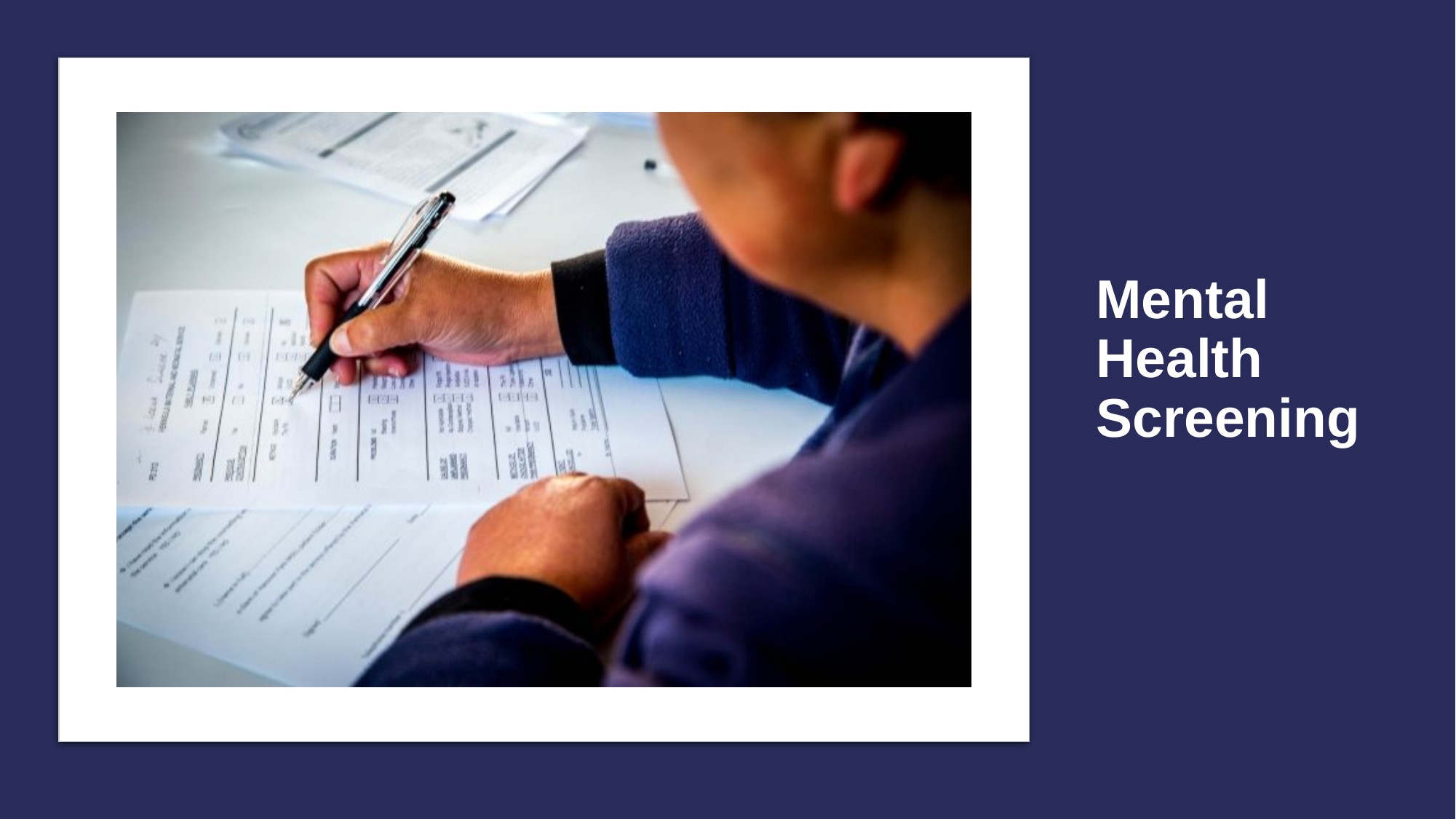

# Mental Health Screening

## Slide 10
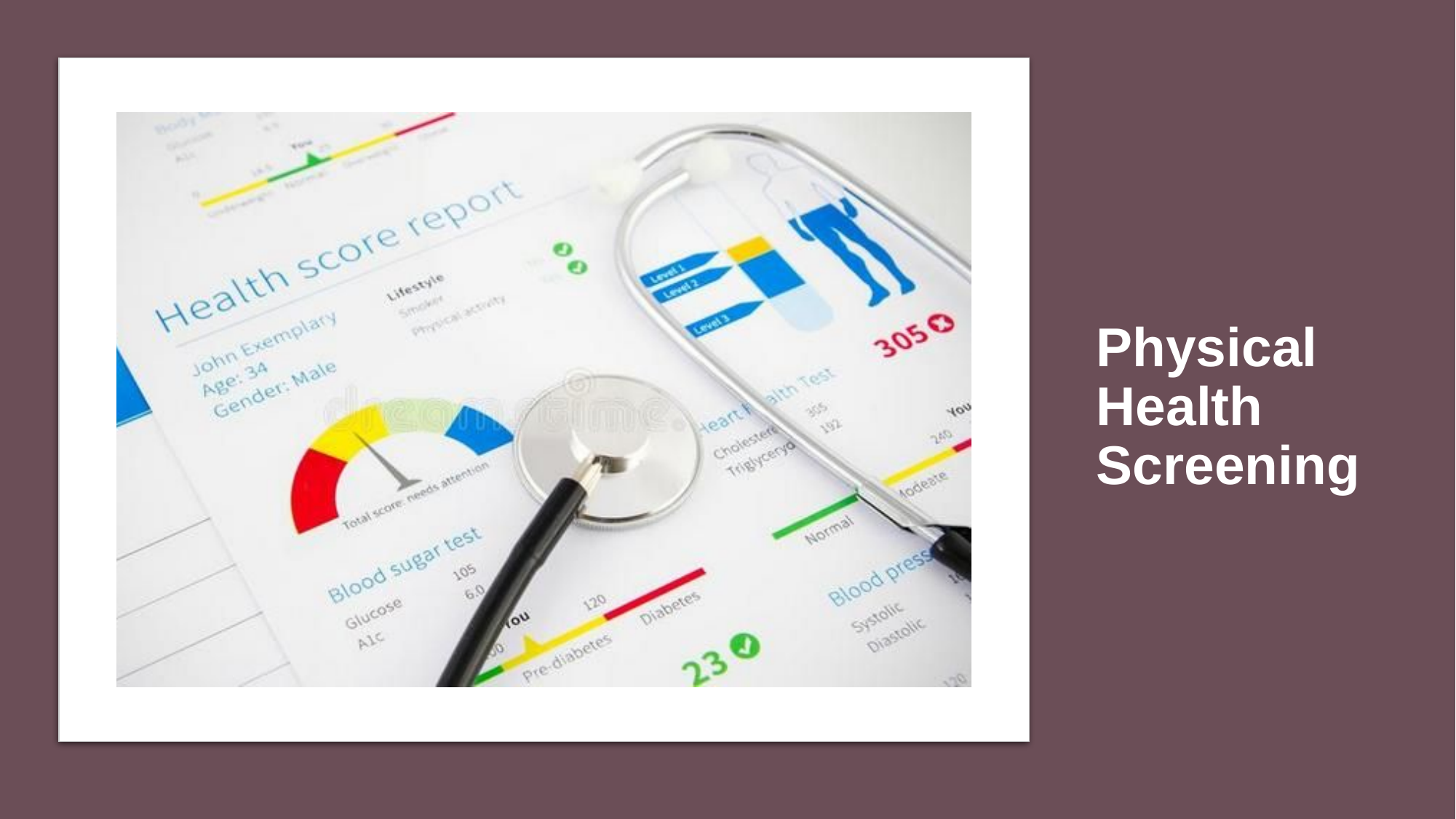

# Physical Health Screening

## Slide 11
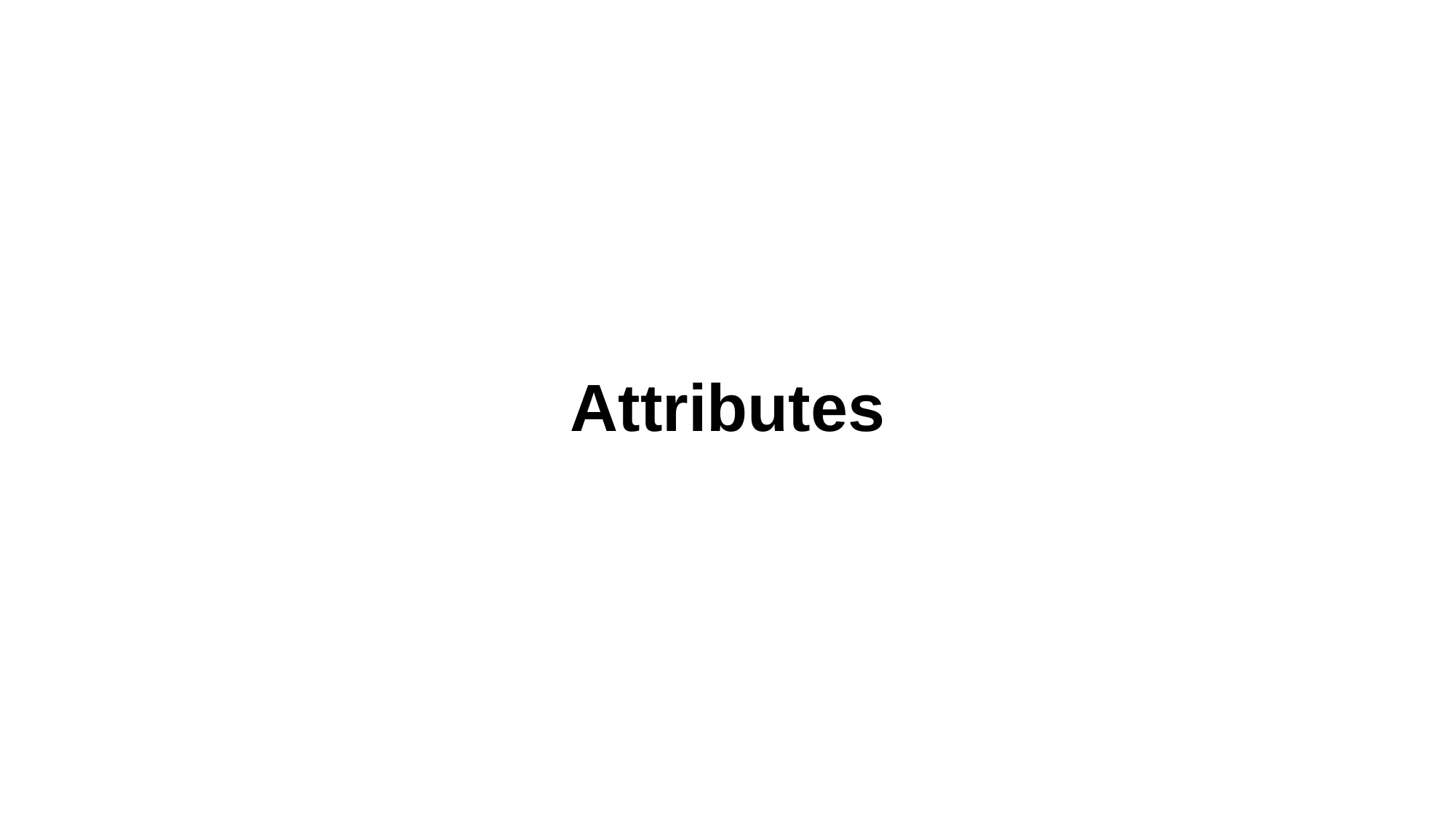

# Attributes

## Slide 12
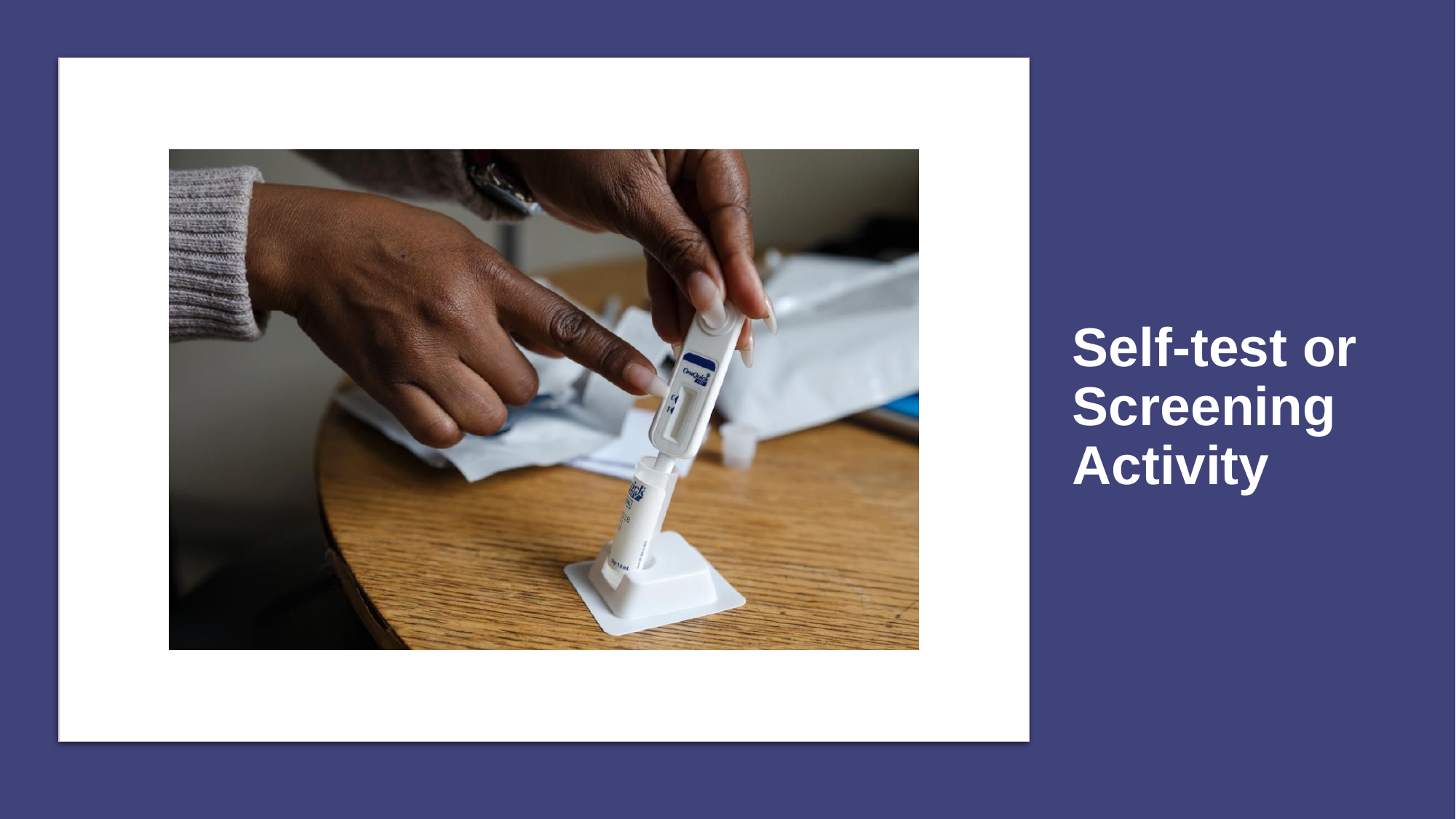

# Self-test or Screening Activity

## Slide 13
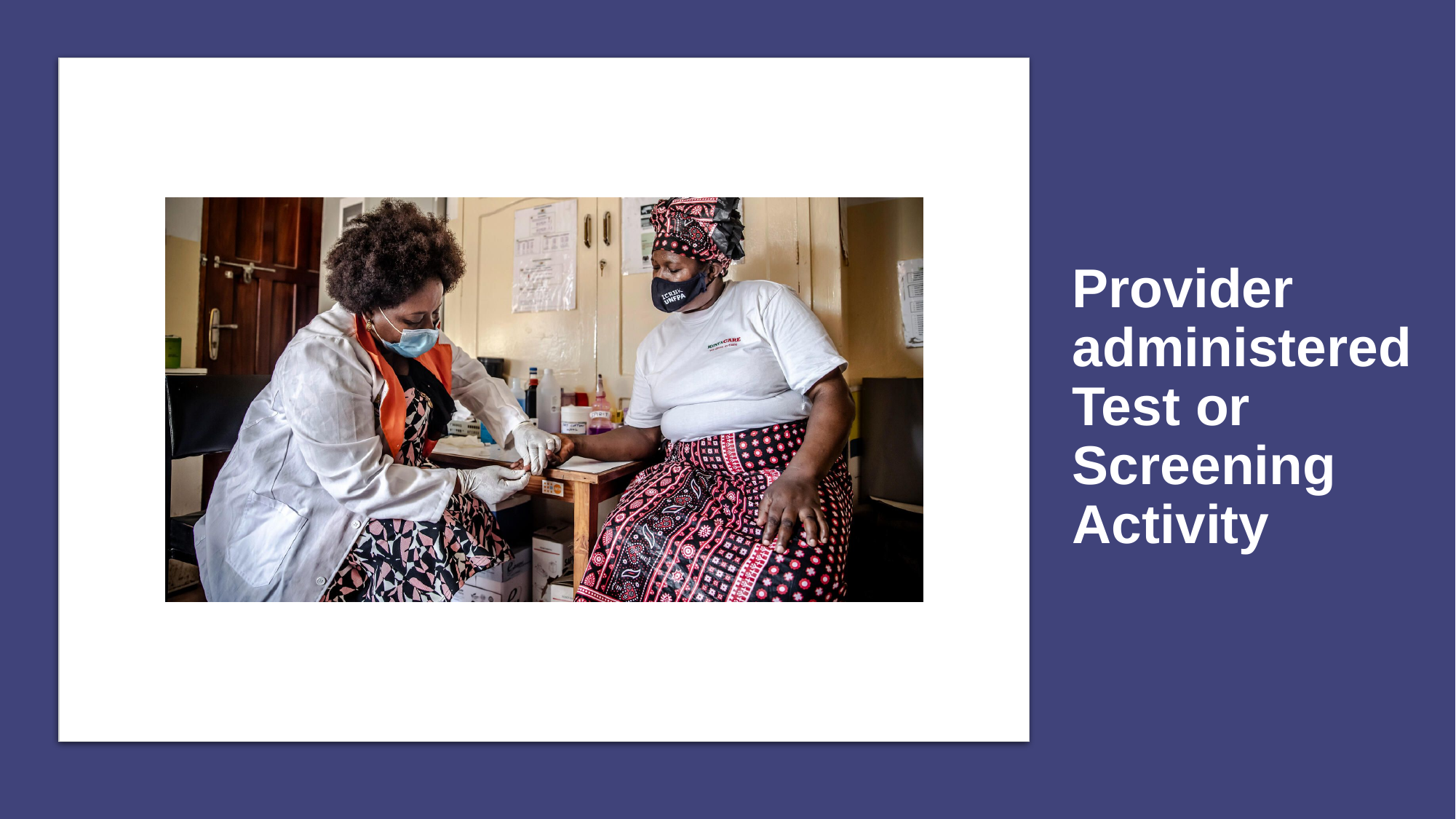

# Provider administered Test or Screening Activity

## Slide 14
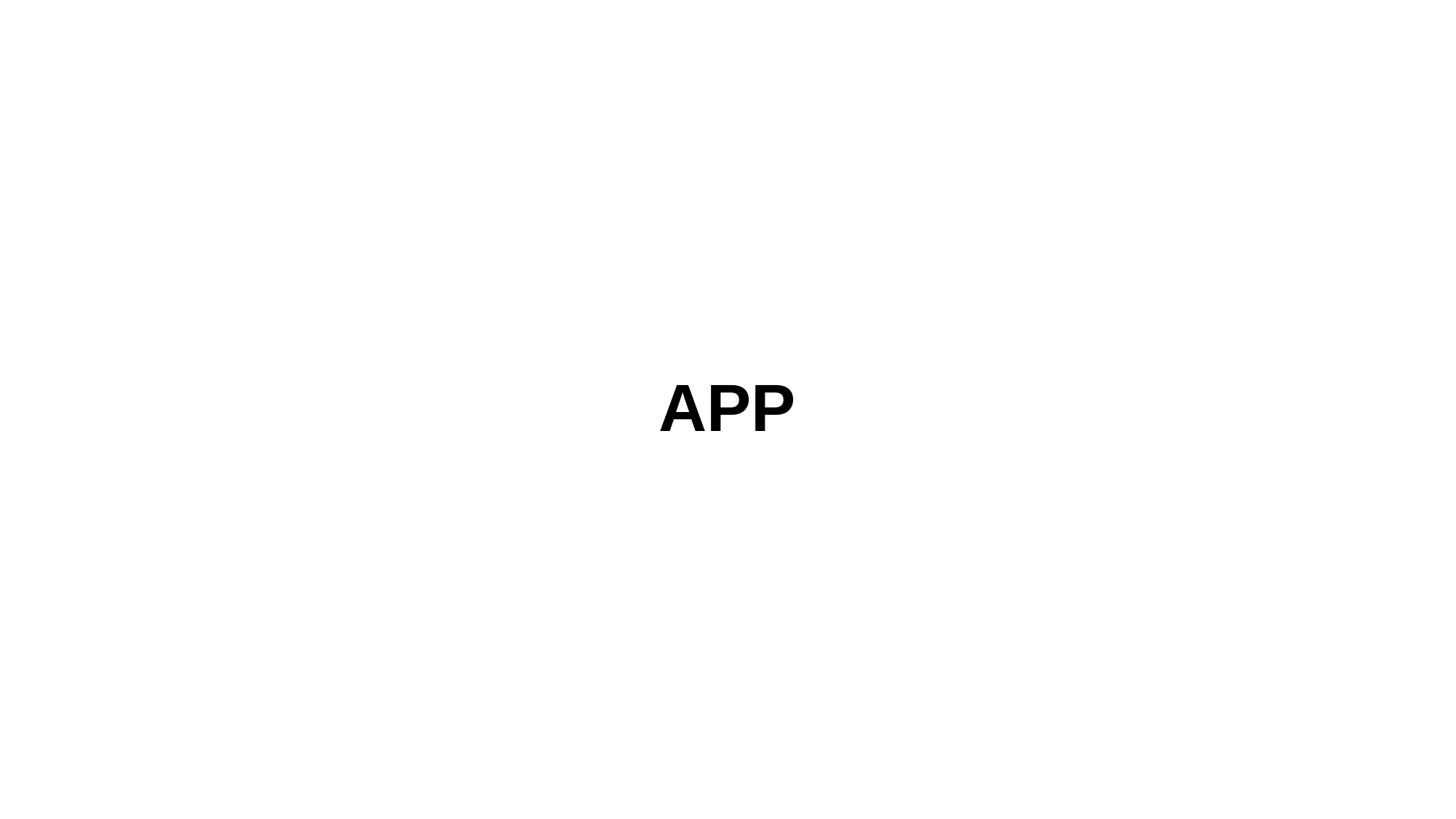

# APP

## Slide 15
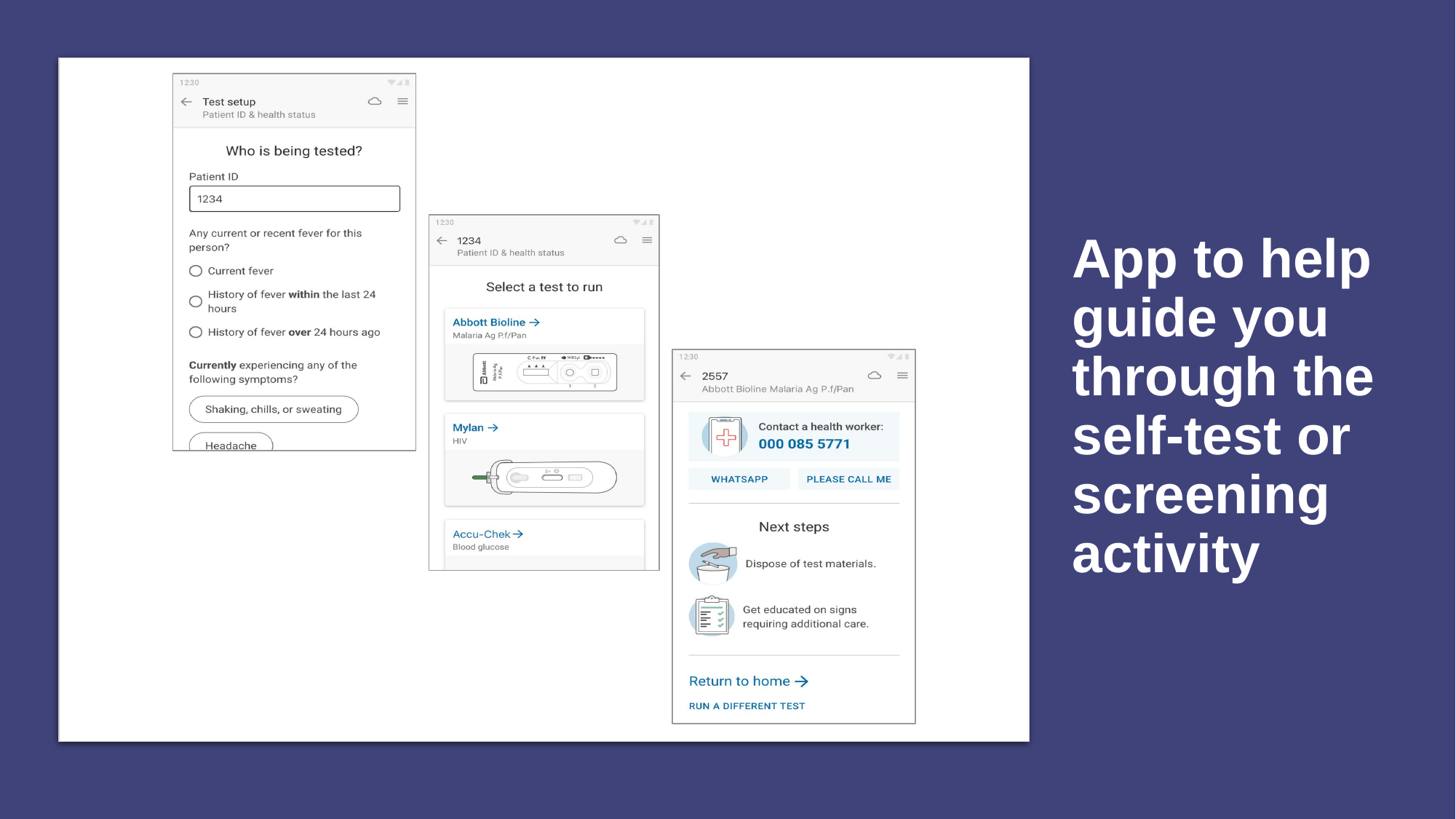

# App to help guide you through the self-test or screening activity

## Slide 16
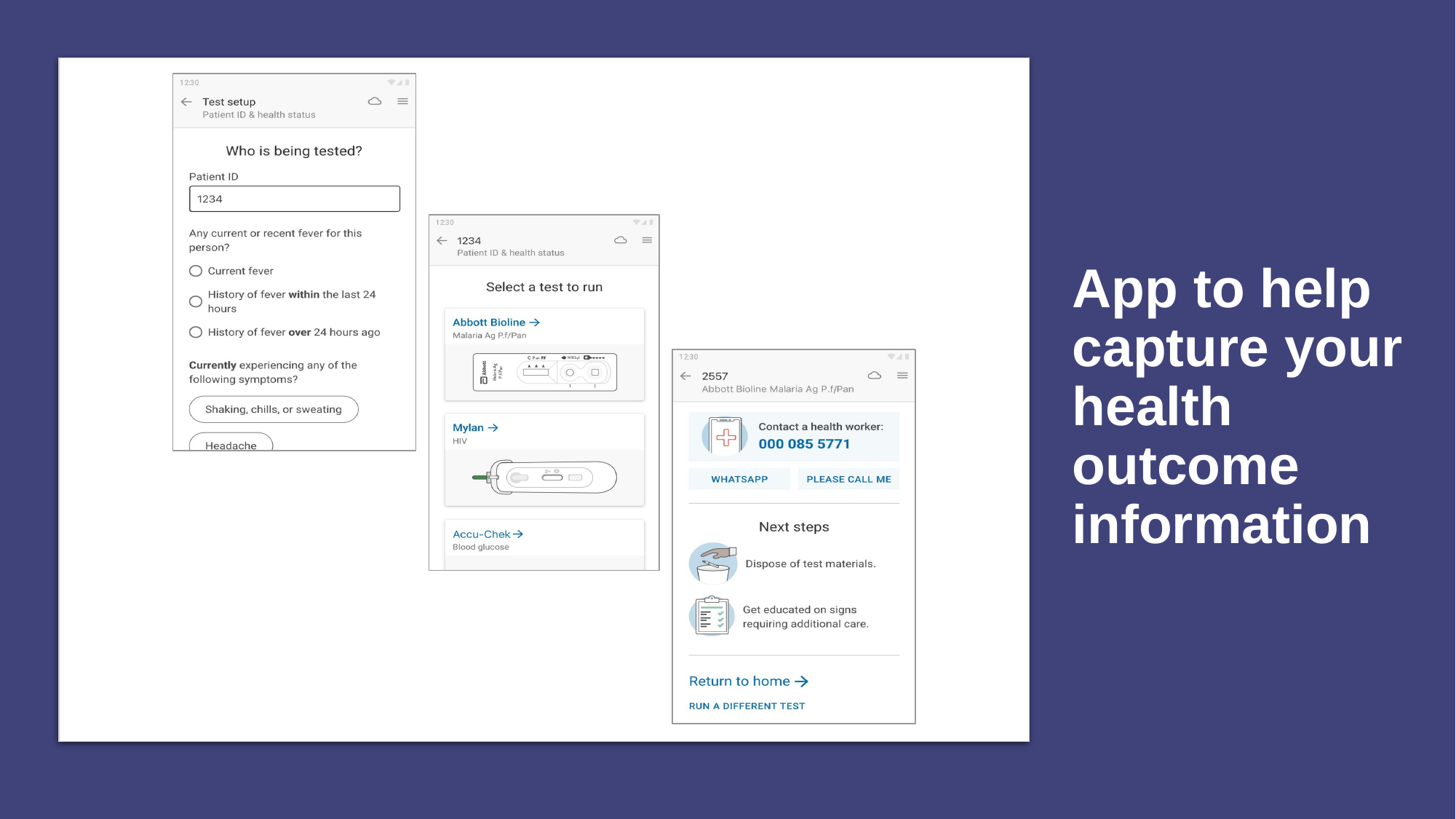

# App to help capture your health outcome information

## Slide 17
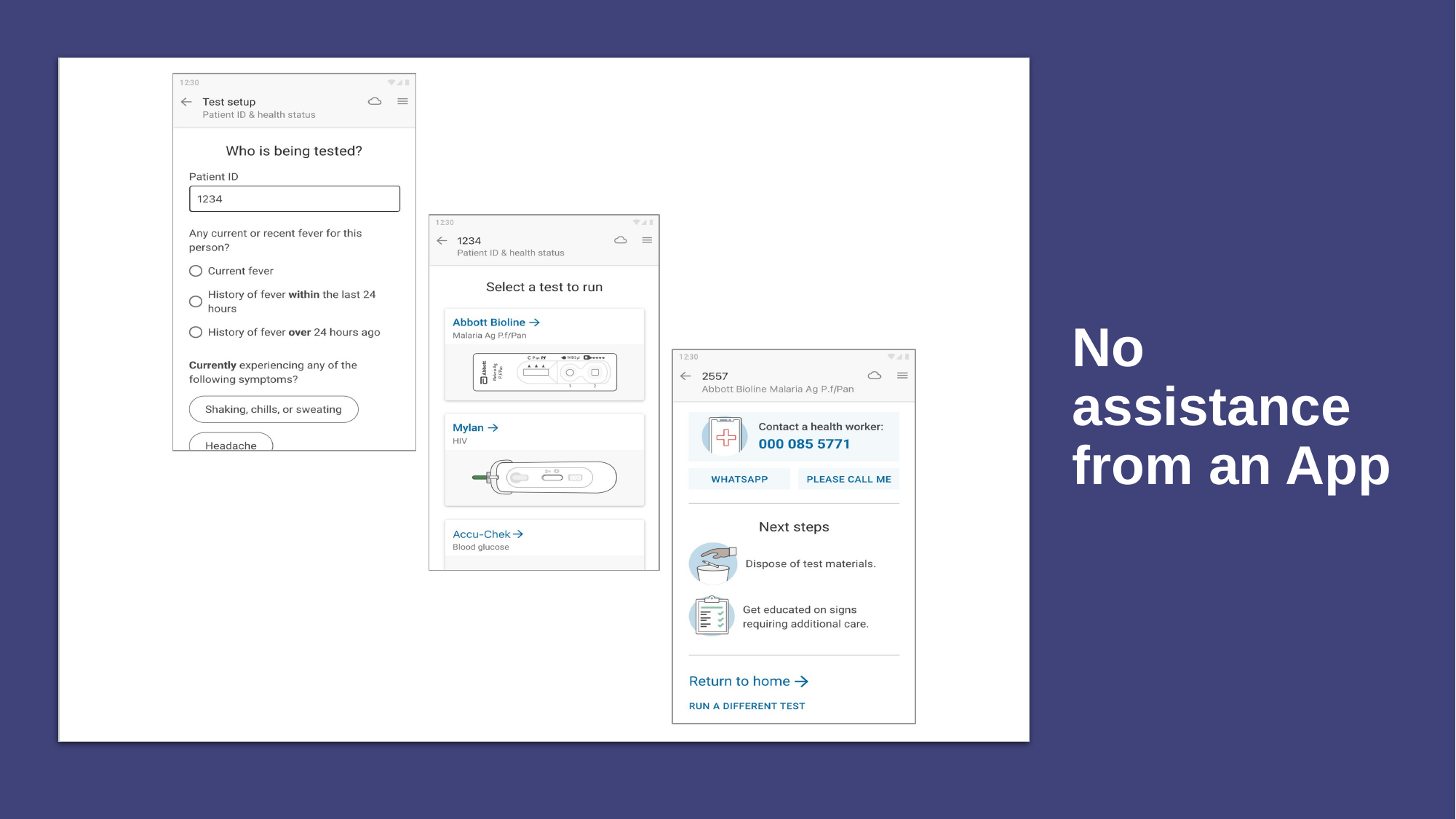

# No assistance from an App

## Slide 18
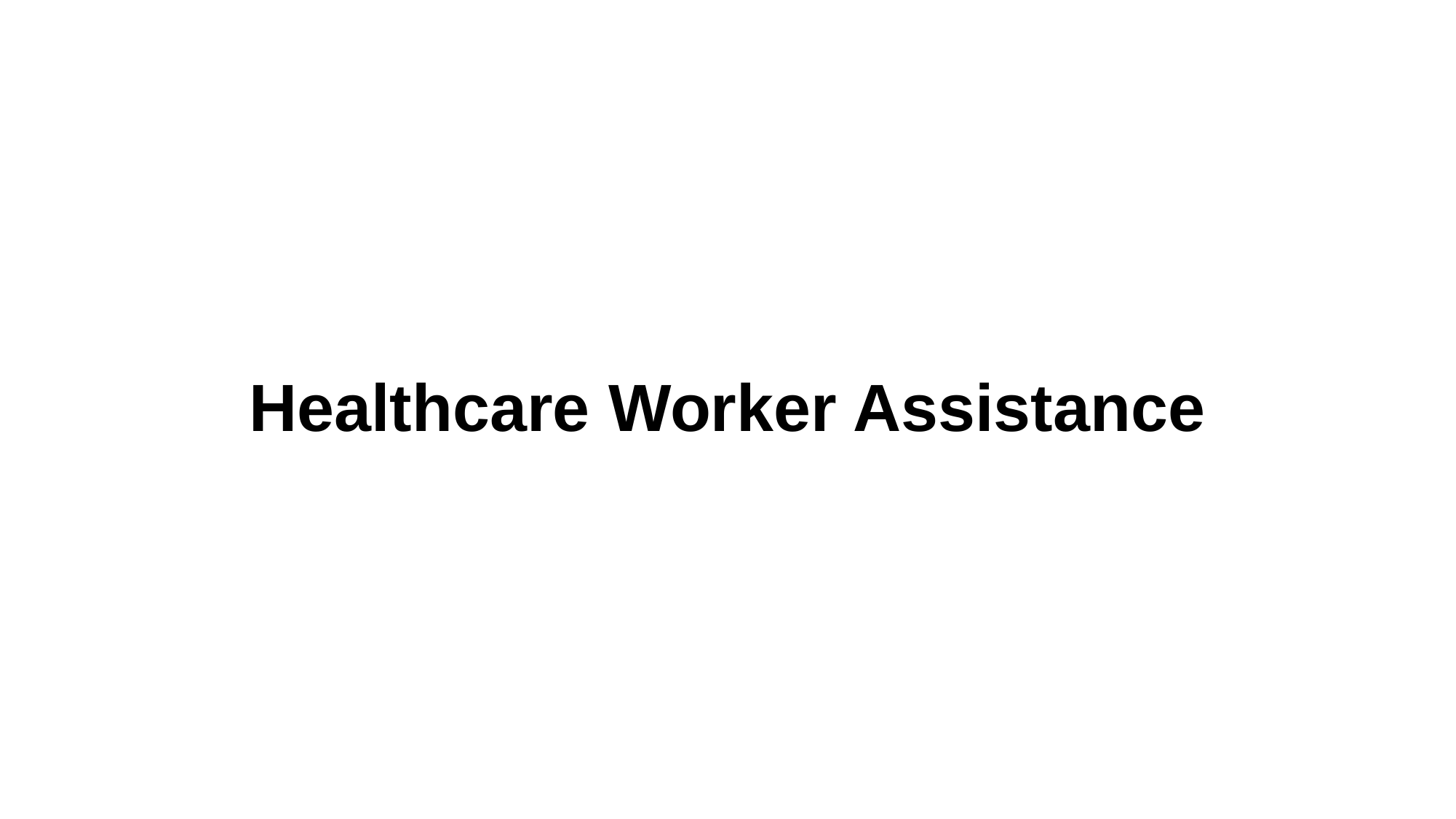

# Healthcare Worker Assistance

## Slide 19
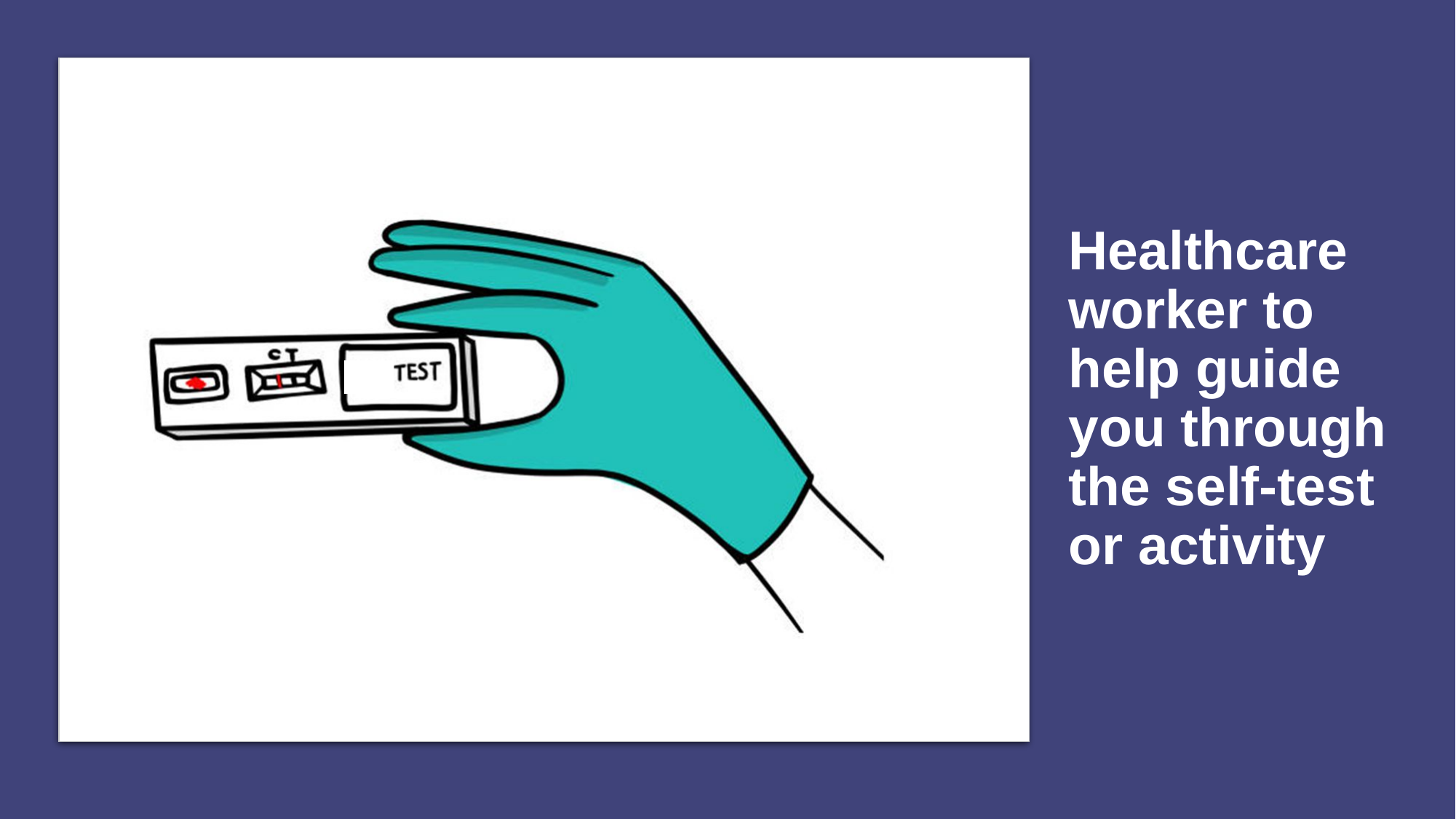

# Healthcare worker to help guide you through the self-test or activity

## Slide 20
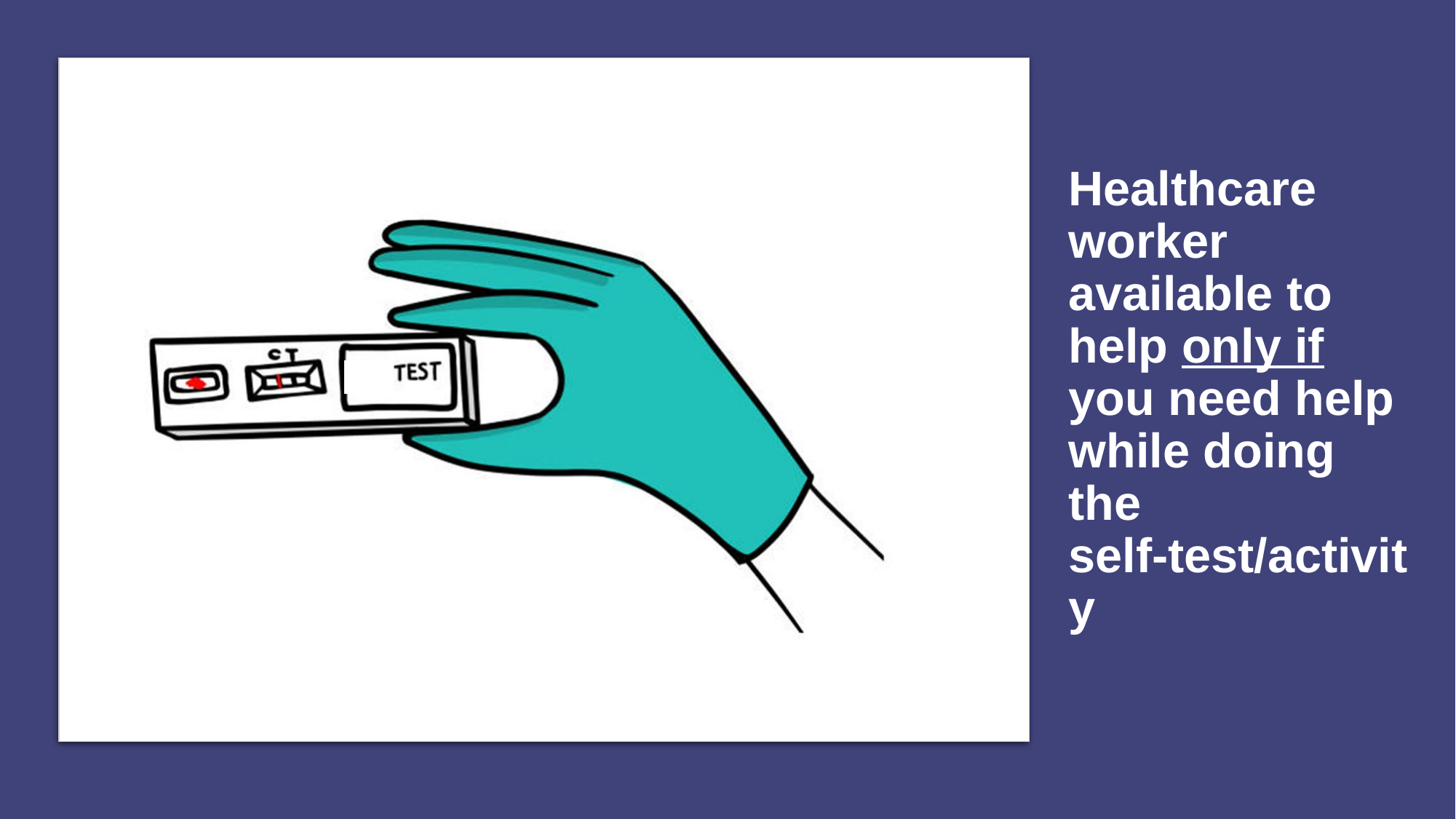

# Healthcare worker available to help only if you need help while doing the self-test/activity

## Slide 21
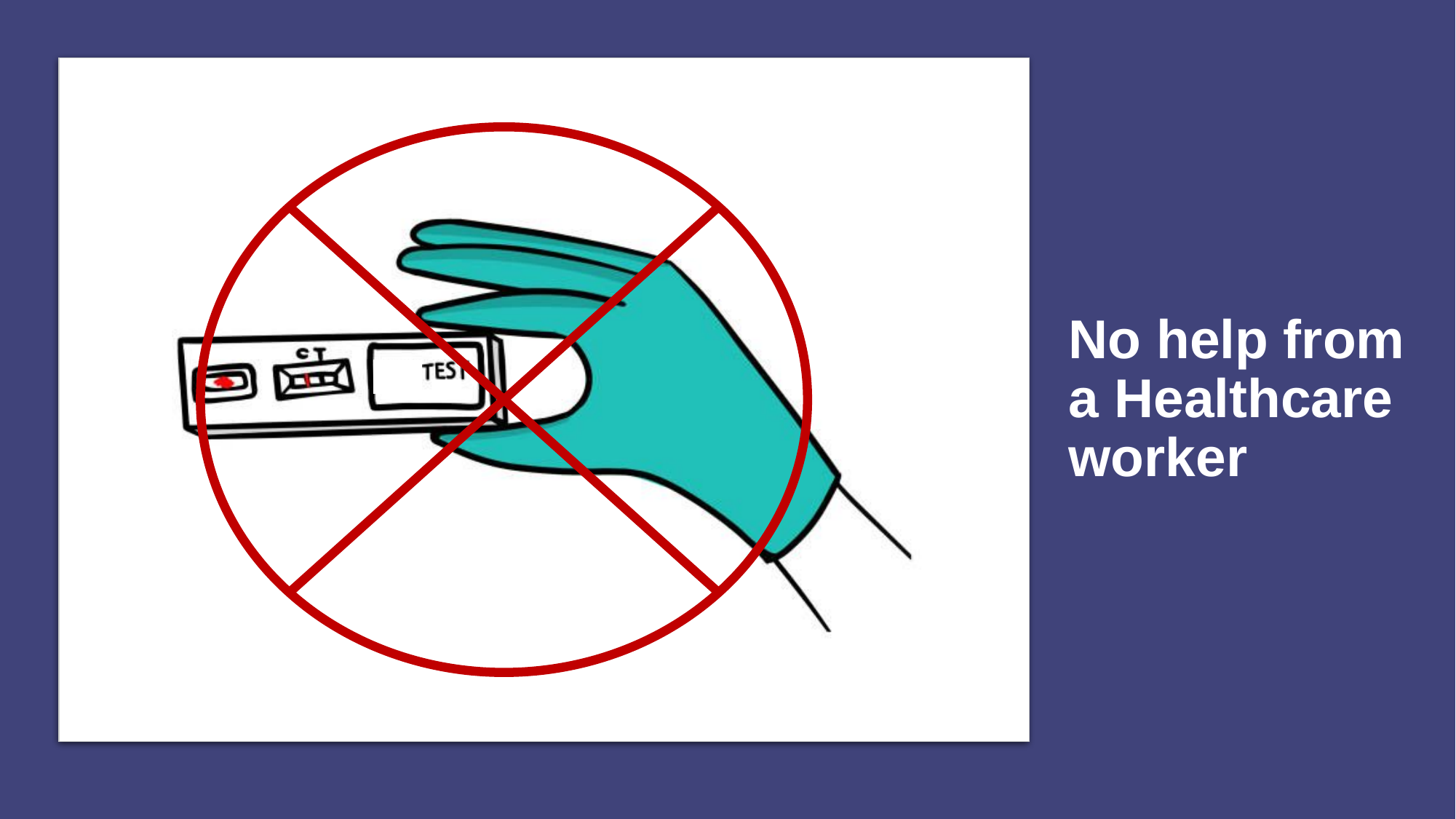

# No help from a Healthcare worker

## Slide 22
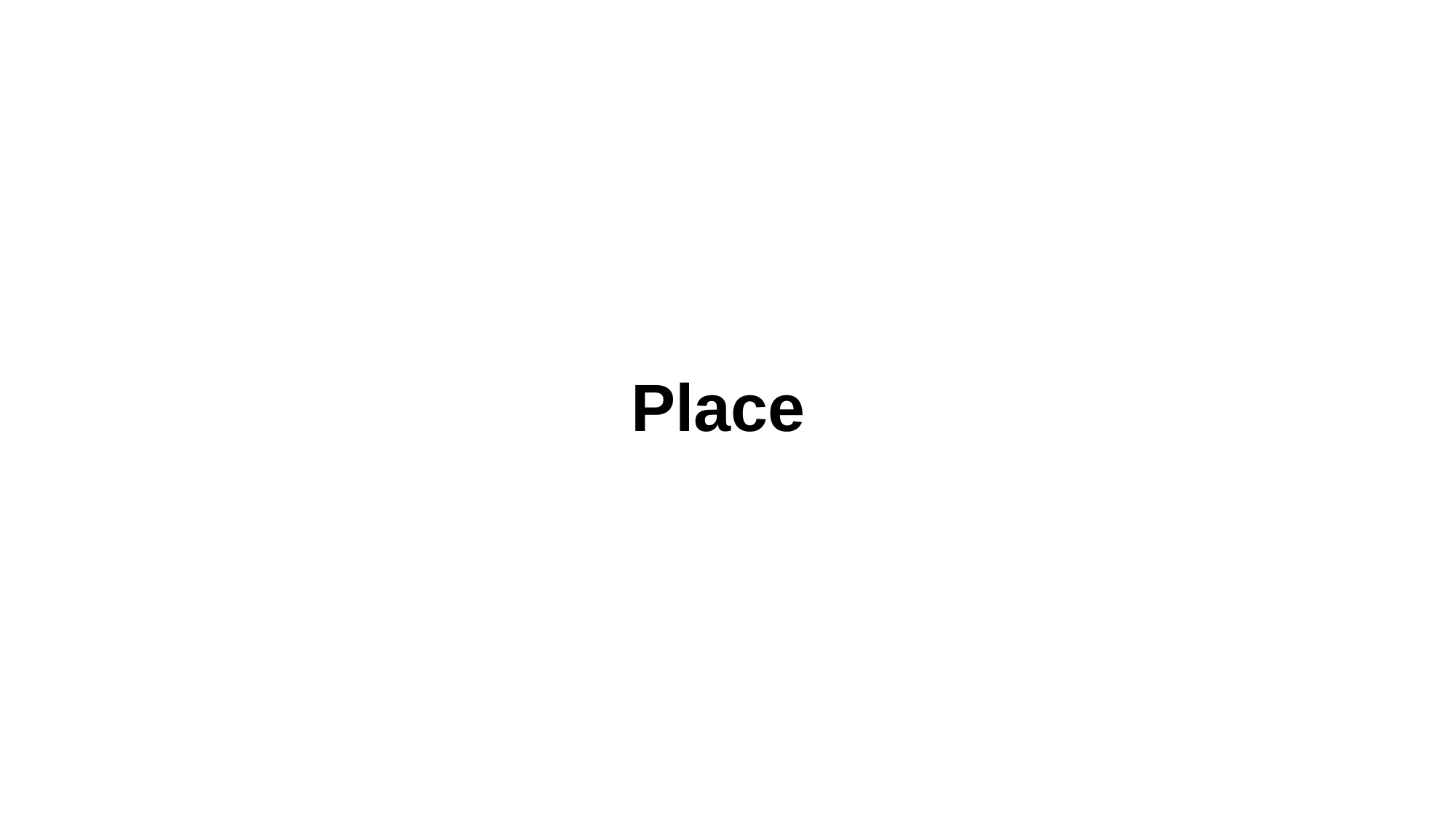

# Place

## Slide 23
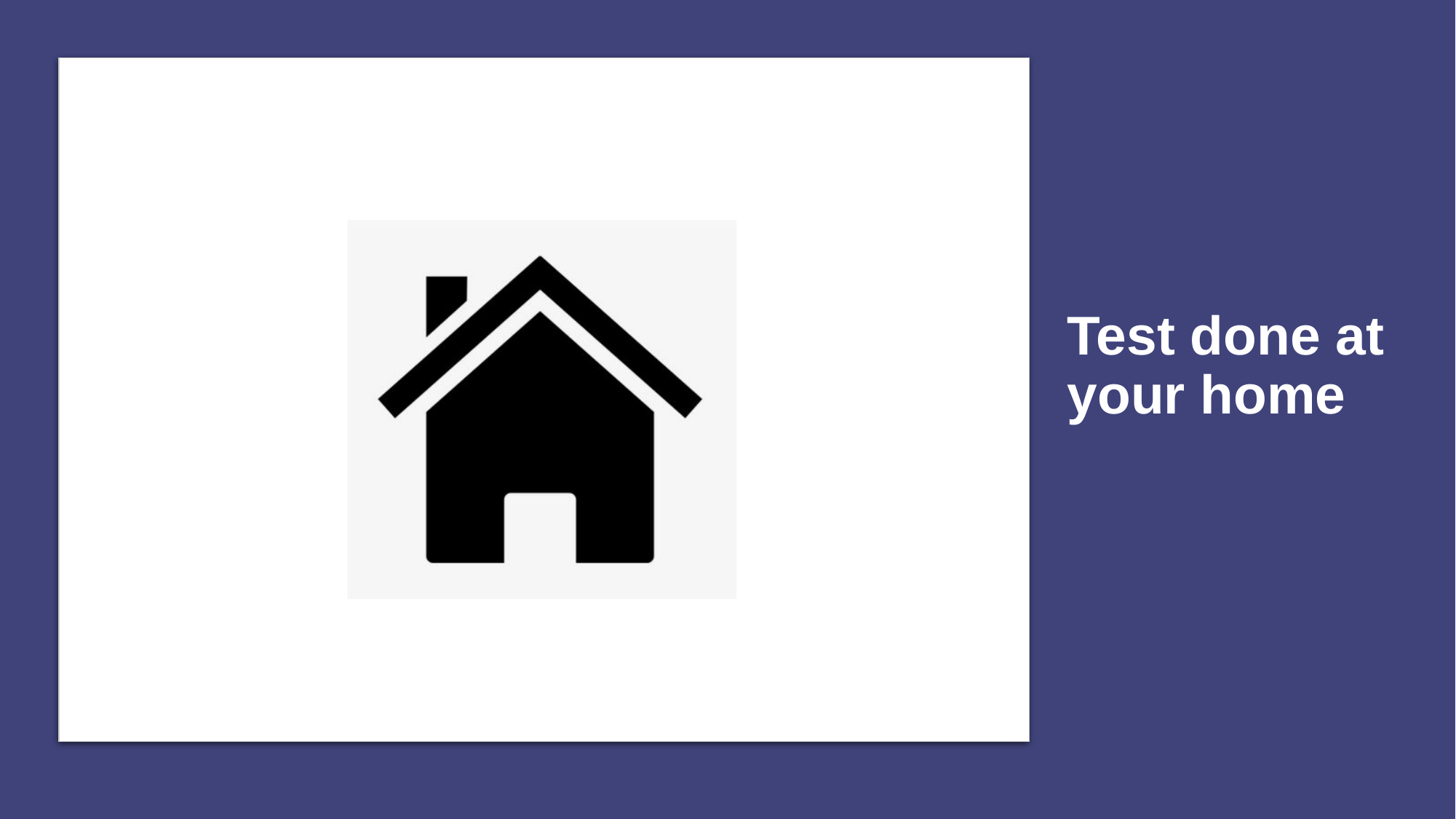

# Test done at your home

## Slide 24
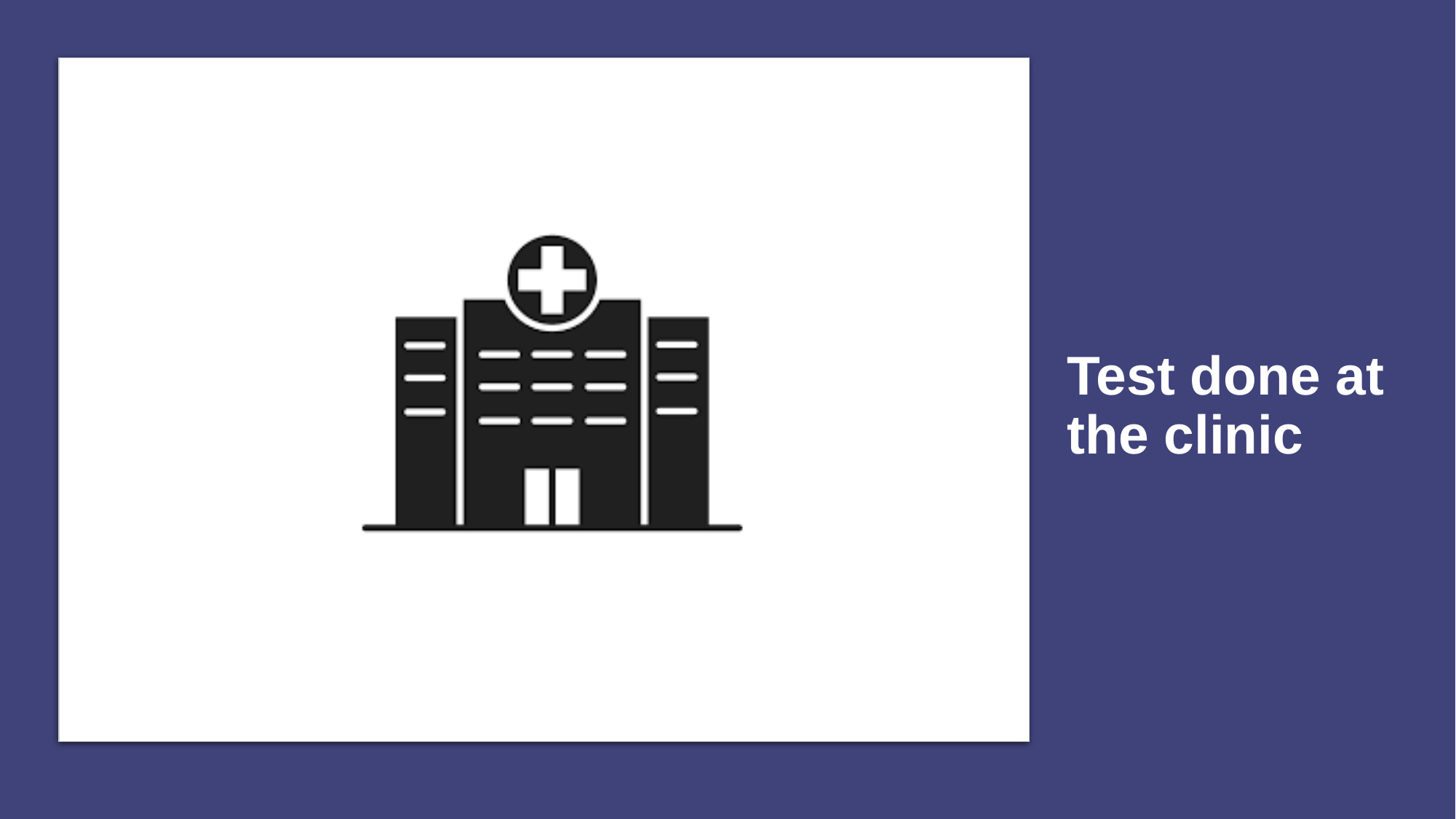

# Test done at the clinic

## Slide 25
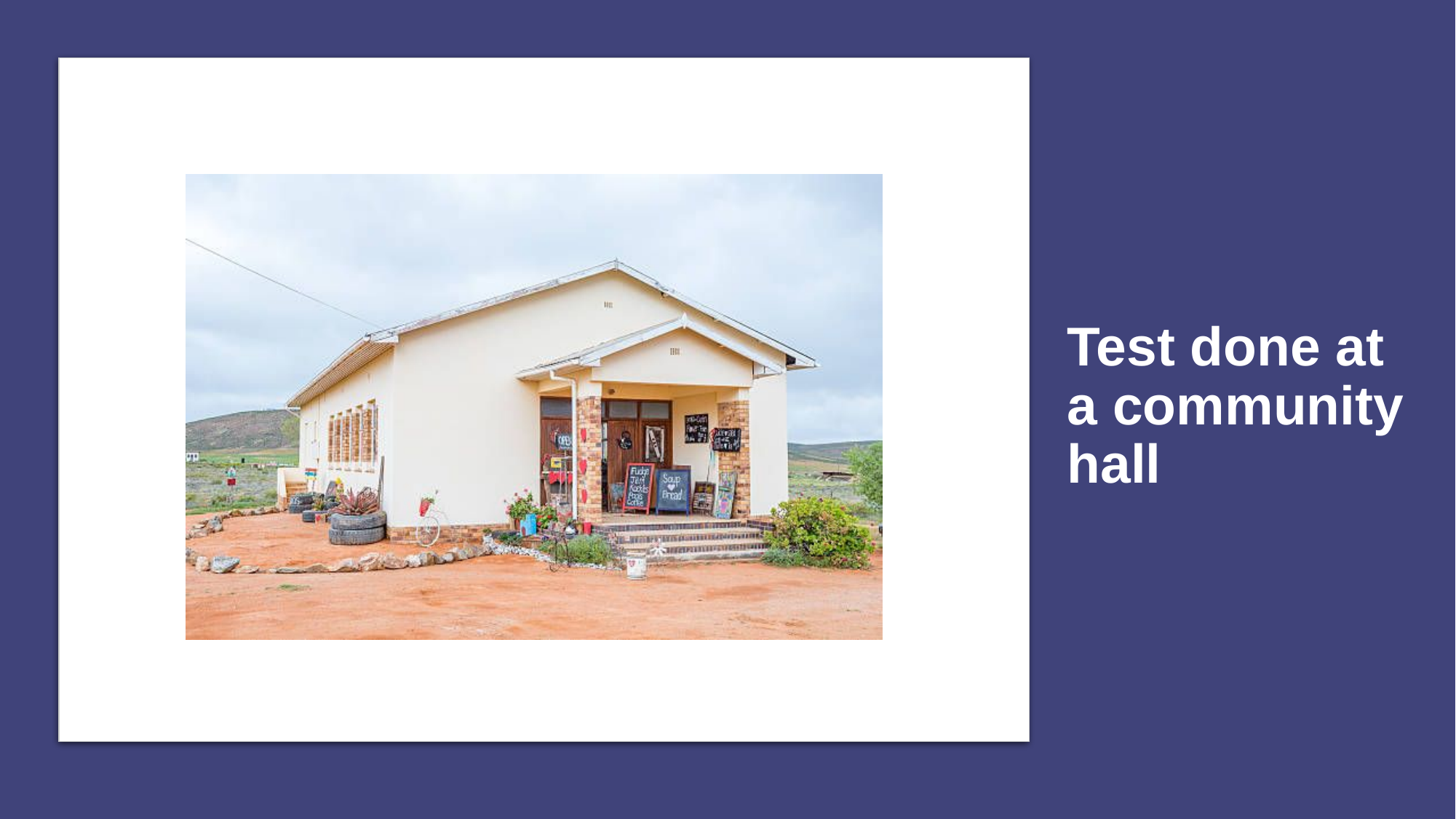

# Test done at a community hall

## Slide 26
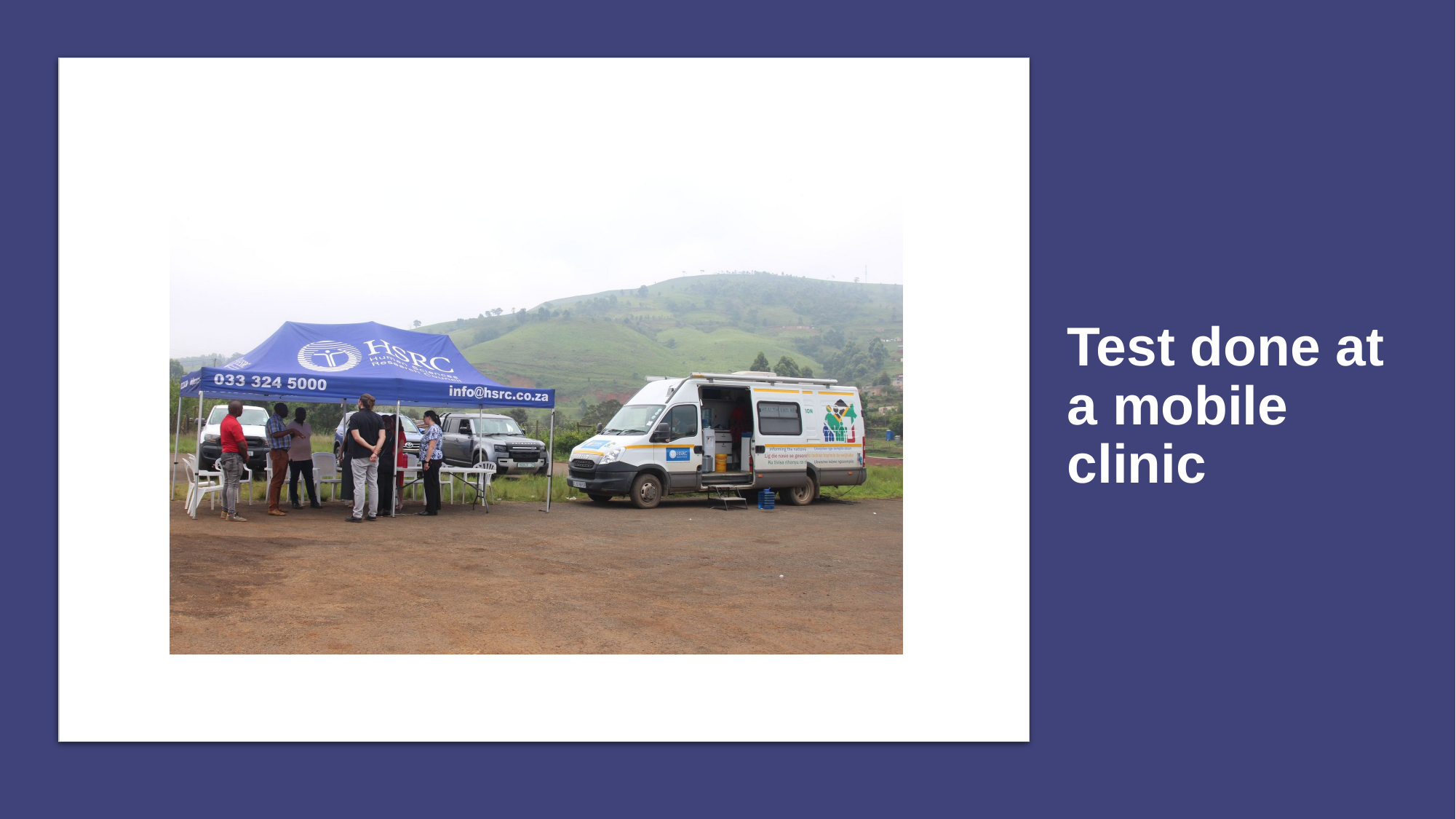

# Test done at a mobile clinic

## Slide 27
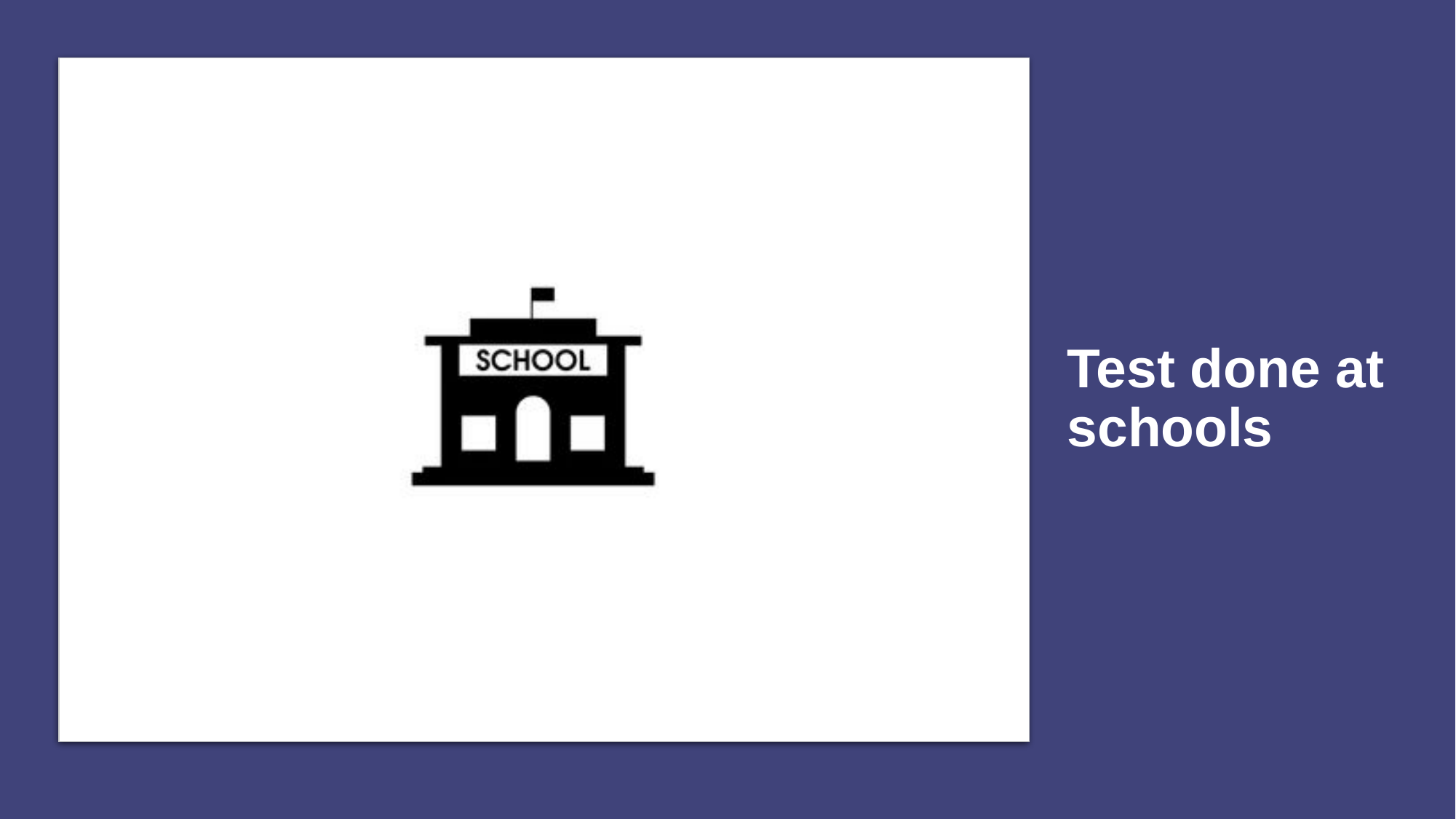

# Test done at schools

## Slide 28
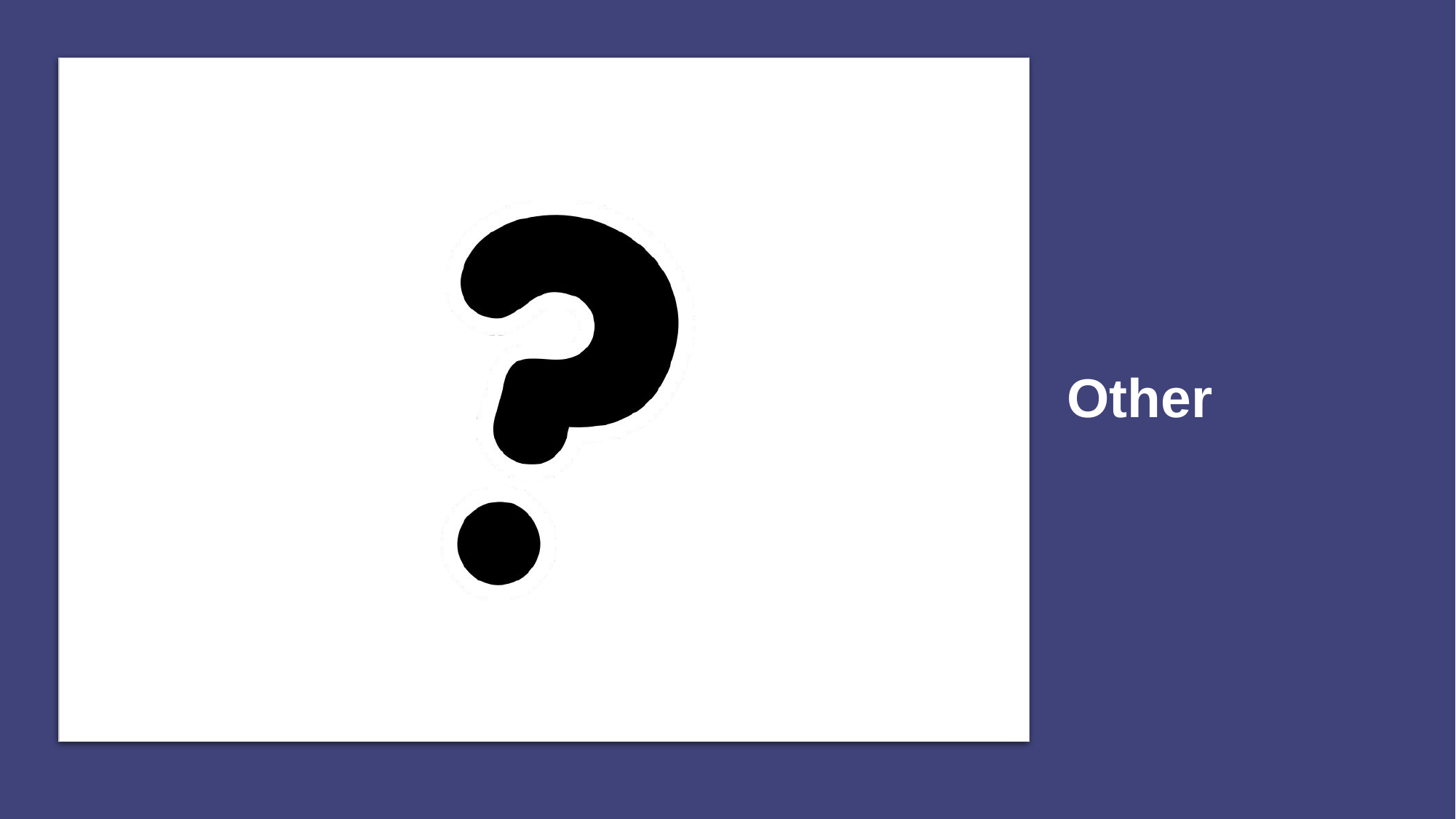

# Other
