# Supplementary material for: Perspectives on App-Assisted Self-Testing Using Rapid Diagnostic Tests Among Community Members, Health Care Providers, and Public Health Leaders in Kenya, South Africa, and Zambia: Qualitative Study
Source: J Med Internet Res. 2025 Nov 26;27:e70273. doi: 10.2196/70273 (PMC12696451; doi:10.2196/70273)
Supplement: Multimedia Appendix 2 [file jmir_v27i1e70273_app2.zip › Multimedia 2 DASH interview guides/5. Interview Guide_Policy makers Program implementers_V0.3_09042023.docx]

**INTERVIEW GUIDE**

**MOH Policy makers/Program Implementers**

**PURPOSE**

One aim of this interview guide is to understand the preferences, feedback, and perspectives of policy makers in the healthcare system to guide a proposed mobile health delivery intervention package.

**INSTRUCTIONS**

There are 2 levels of questions:

• **Numbered questions (1, a, etc):** these questions **must be asked** and discuss with participants.

• **Bulleted Probes:** to serve as suggestions for the facilitator rather than a strict list of questions that *must* be asked. So, **depending on what has already been discussed, and the IDI context, you may ask these probes or not or may phrase probes differently** to try and better understand what the participant is trying to communicate.

**MATERIALS**

1. App phone demo or print-out (**2-3 sets** **to be laminated and shared between participants**)
2. Audere assets first 4 pictures (specific to Policy makers)

- Instructions/suggestions to interviewer are in *italics*.

**Background**

1. Please tell me about your role in the MOH/organization.
   - What are your main responsibilities?

**Priority Diseases**

1. Which are the diseases that [*the country*] will focus on for the next 5 years?
2. Which other diseases/conditions would benefit from self-administered rapid diagnostic testing?

*Probe:*

- For HIV?
- For diabetes?
- For hypertension?
- For malaria?
- Pregnancy?
- STIs?

1. In order of priority, which top 3 diseases do you think should be prioritized for rapid testing in the communities that you serve, and why?
   - What purpose would it achieve?
   - How would it benefit the healthcare system?
2. What is the risk to the healthcare system if people start self-testing for various conditions?
   - 1. What concerns do you have about making home-based diagnostic tests available to communities? (*ability, trust, regulatory framework*)
   - What are the limitations of rapid self-testing?
3. Which venues are suitable for self-testing? (*where should people self-test?*)
4. What steps do you think a person must take after self-testing in order to get the best possible health outcome?

**Digital interventions**

[**Interviewer,** *please show the phone demo of the app*]

8. What do you think about patients having access to an App that can guide them through self-testing and next steps, such as not unnecessarily visiting the clinic or using it to share results and get medical advise from a healthcare provider via the app? [*show prototype of proposed study App*]

- - What purpose would it achieve?
  - How would it benefit the healthcare system?
  - What is the risk to the healthcare system/limitations of using an app get medical advice for various conditions through an app?

9. What do you think about the patient using an app to get a prescription?

- - What would be the benefit?
  - What would be the concerns?

10. What do you think about the patient using the App to order the medication for home delivery itself?

- - What would be the benefit?
  - What would be the concerns?

11. What do you think about patients having access to their medical records, for e.g., test results through this app?

- - What would be the benefit?
  - What would be the concerns?

**Innovations**

***Interviewer, ask Policy makers*:**

**12.** What are the processes for bringing in new rapid diagnostic tests and related digital health interventions, such as apps, into policy?

- - What information do you need to make these decisions?

***Interviewer, ask Program implementers*** *(NGO, Healthcare providers)*

**13.** What would be the processes for implementing policy on new rapid diagnostic tests and related digital health interventions? What challenges do you face in supporting implementation of new policy?

- - What solutions have worked in countering these challenges in the past?
