# Supplementary material for: Perspectives on App-Assisted Self-Testing Using Rapid Diagnostic Tests Among Community Members, Health Care Providers, and Public Health Leaders in Kenya, South Africa, and Zambia: Qualitative Study
Source: J Med Internet Res. 2025 Nov 26;27:e70273. doi: 10.2196/70273 (PMC12696451; doi:10.2196/70273)
Supplement: Multimedia Appendix 2 [file jmir_v27i1e70273_app2.zip › Multimedia 2 DASH interview guides/2. FGDs_ V0.3_Community members_9042023.docx]

**FGD GUIDE**

**Community members**

**Version 0.3**

**PURPOSE**

The purpose of this FGD guide is to understand the preferences, feedback, and perspectives of health care providers in the healthcare system to guide a proposed mobile health delivery intervention package.

**INSTRUCTIONS**

There are 2 levels of questions:

• **Numbered questions (1, a, etc):** these questions **must be asked** and discuss with participants.

• **Bulleted Probes:** to serve as suggestions for the facilitator rather than a strict list of questions that *must* be asked. So, **depending on what has already been discussed, and the FGD context, you may ask these probes or not or may phrase probes differently** to try and better understand what the participant is trying to communicate.

**MATERIALS**

1. Number cards 1 to 6 for each participant (**to be printed and laminated**)
2. Flip chart with/without stand and markers; notecards maybe used instead of flipchart
3. Test and screening activity cards (**2-3 sets** **to be laminated and shared between participants**)
4. App phone demo or print-out (**2-3 sets** **to be laminated and shared between participants**)

- Instructions/suggestions to facilitator are in *italics*.

*Please remind participants to not use names, rather to refer to themselves and each other by the number card provided. When they speak, participants should state their card number. Also remind participants that everything said in the room should remain confidential. However, because this is shared information/confidentiality, we cannot guarantee that participants will not repeat information. Therefore, for anything private/sensitive, participants may want to withhold information/views and share with facilitator later.*

*Set group rules. E.g., talking one at a time, being respectful, etc*

**Ice-breaker**

1. Going around the circle, in one word, express what you love about your community.

**Disease priorities for testing**

***Facilitator,*** *please make 3 columns flipchart labeled common diseases, priority of rapid testing, priority for self-testing. Note diseases on 1^st^ column. If no flipchart, notecards may be used, 1 per disease. Each person can choose which 3 cards should be on top for rapid testing and which 3 for self-testing*



***Note-taker*** *take notes on note-pad; if possible at end of session take a photo on your phone/tablet*

1. Now this time around, we would like you to go around the circle, listing what you think are common diseases for which your community should be tested? We will stop when no more diseases are named.



***Facilitator*** *Note diseases on 1^st^ column/separate notecards*

1. Of these diseases, which would be the top three that should have:
   - a test that can quickly give a diagnosis?


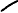


(***Facilitator*** *put a line i.e., | against for each disease listed among top three diseases by each person in 2^nd^ column, crossing out with every 5^th^ line i.e., ||||* )

- - a test that you can use yourself?

(***Facilitator*** *put a line against for each disease listed among top three diseases by each person in 3^rd^ column as above*)

1. What other conditions are suitable for home-testing?

***Facilitator****, list in different colour marker*

- - What about pregnancy test?
  - What about STIs?

**Patient Experience Map**

So far we have talked about different diseases. Now I want you to thinking about what typically happens when somebody falls ill in your community:

1. What are the steps people take when they or their family members have symptoms of an illness before going to a healthcare facility? ***Facilitator****, please note each step on flipchart/ one notecard per step*

- Self-medicate
- Home remedies
- Traditional healer
- Other

1. For each of these steps, ***Facilitator****, please note under each step on flipchart or on each notecard*
   - What are the reasons for taking these steps?
   - What maybe challenges the person faces when taking these steps?
   - What could be done to address these challenges?
2. In your experience, when you’ve decided to go to a clinic or hospital…
   - How would you describe the care that you received?
   - What challenges did you experience?
   - How equipped did you feel to take care for yourself after you left that clinic or hospital?

**Package of interventions**

Now let’s suppose **you have one of these rapid tests at home**.

***Facilitator****, show cards 1-10 from tests and screening activity cards*

1. How would you feel conducting the test yourself?

***Facilitator****, show card 12 from tests and screening activity cards*

- - Please tell me more about why you feel this way

1. How would you feel conducting the test with the help of a healthcare worker?

***Facilitator****, show cards 13 & 19 from tests and screening activity cards*

- - Please tell me more about why you feel this way

1. How would you feel conducting the test with the help of a health careworker **only if you need it? *Facilitator****, show cards 13 & 20 from tests and screening activity cards*
   - Please tell me more about why you feel this way

Now let’s suppose **you have one of these rapid tests at home along with a mobile app**. ***Facilitator****, demonstrate app or show laminated photos of process or show card 15 from tests and screening activity cards*

1. What are your views and perceptions about such an App for helping you to self-test?
   - What would be the challenges of using such an App?
   - What would be the benefits of using such an App?
   - What concerns do you have about using such an App?
2. What do you think about the usability of such an intervention?
   - How easy would it be to use the App?

- What could make it difficult to use the App?
- How appropriate is such an intervention for:
  - - Differently abled
    - Gender
    - Social status (marital/single)
  - How trustworthy would such an app be?

1. Thinking of the area you live in, what concerns would you have about the functionality of the App?

- Access to electronic device, airtime/data, insufficient band width, etc.)

1. Say you received a positive result for a self-test. What are your preferences for receiving more information about that positive result?
   - Receiving more information through an app?
   - Virtually, in real-time with a provider?
   - Virtually, video call or messaging?
   - In-person, at home?
   - In-person, at a clinic?
2. Say you received a positive result for a self-test that requires follow up care at a health facility (clinic or hospital?). What do you think would make it easier or harder to get to that facility to get the care that you need?
   - Job considerations?
   - Family commitments?
   - Proximity to the facility?
   - Transportation?

Now let’s suppose **the mobile app can be used to 1) interpret your results and guide you on what to do next**

***Facilitator****, show results page on app or on laminated photos of process or show last picture on card 15 from tests and screening activity cards*

**and 2) note your test history**

***Facilitator****, follow test history link after results page on app or on laminated photos of process*

**And the app can also be used to** 3) consult a healthcare provider, 4) send request for medication delivery to your home, and 5) note your treatment, how your health and treatment is going, and any referrals to another healthcare facility or provider.

1. Thinking of the features of the App, what challenges/barriers to getting healthcare could the App help overcome?

a. What other challenges can the app address?

1. What could be better alternatives to the App?
   1. What would you rather use or who would you rather turn to instead of using the App?
2. What existing practices/innovations could it be integrated with?

a. What other resource would make a perfect combination with the App?

19. How could the App be improved to make it more useful?

- What features should be added, and why?
  - What features should be removed, and why?
  - What other purpose could such an App serve?

**Testing Locations**

20. In your community, where do you think you would go to pick up a testing package?

- (Local pharmacy? Church or place of worship? A school? A hairdresser/barber? Community hall)

1. Would you be willing to go to one of the locations you have listed to pick up a package when you need one?
2. How does this differ when it comes to the type of test you want to pick up?
3. Are there specific individuals in your community you would trust to get a test package from?
