# Supplementary material for: Perspectives on App-Assisted Self-Testing Using Rapid Diagnostic Tests Among Community Members, Health Care Providers, and Public Health Leaders in Kenya, South Africa, and Zambia: Qualitative Study
Source: J Med Internet Res. 2025 Nov 26;27:e70273. doi: 10.2196/70273 (PMC12696451; doi:10.2196/70273)
Supplement: Multimedia Appendix 2 [file jmir_v27i1e70273_app2.zip › Multimedia 2 DASH interview guides/3. Interview Guide_Community Members_V1.2_9042023.docx]

**INTERVIEW GUIDE**

Community Members

One aim of this interview guide is to understand the preferences, feedback, and perspectives of community members to guide a proposed mobile health delivery intervention package.

**INSTRUCTIONS**

There are 2 levels of questions:

• **Numbered questions (1, a, etc):** these questions **must be asked** and discuss with participants.

• **Bulleted Probes:** to serve as suggestions for the facilitator rather than a strict list of questions that *must* be asked. So, **depending on what has already been discussed, and the interview context, you may ask these probes or not or may phrase probes differently** to try and better understand what the participant is trying to communicate.

**MATERIALS**

1. RDT (malaria/HIV) with brochure laminated
2. Test and screening activity cards (**2-3 sets** **to be laminated and shared between participants**)
3. App phone demo or print-out (**2-3 sets** **to be laminated and shared between participants**)

- Instructions/suggestions to facilitator are in *italics*.

**ICE-BREAKER**

1. Can you describe your typical day to me.

**Attributes of Intervention**

1. Now there are ways that we could bring tests for certain conditions to you, so that you could complete yourself, meaning that you would not have to come to the clinic for them. You may have heard about home pregnancy test kits for instance. I am going to take you through the steps of some of these tests and ask you some questions.

[**Facilitator,** *Go through the steps for completing the RDT*].

- 1. Where would you complete these RDT steps?
  2. When would you be best able to do it within your typical day?
  3. What concerns would you have about being able to do the test by yourself (self-efficacy beliefs)
     1. What would help you to use these RDTs more effectively?
  4. What would be the benefits of using these RDTs?
     1. what would be the value-add of these RDT to existing healthcare options.
  5. What would be the challenges of using these RDTs?
- Probe for structural/social/individual barriers)
  1. How would using these RDTs fit into your life?
     1. When would you use this instead of going to the clinic.
  2. How would your partner/family feel about you doing these tests?
     1. What sort of support would you need from them to do these tests?

1. Now if you had to do these tests at home on your own how would you prefer to receive instructions?
   1. Something like this brochure, like a step-by-step guide with pictures and words? Watch a video?

Have instructions read aloud?

Have someone guide you over the phone? In person?

**If we made rapid tests available at your home and an app on your phone could help you read your results like this:**

[**Interviewer***, show cards 1-10 from tests and screening activity card* ***&*** *show results page on app or on laminated photos of process or show last picture on card 15 from tests and screening activity cards]*

1. Would you rather test yourself at home, or would you prefer to go to a health facility for diagnosis?
   1. Would this be true for all the different tests?
      1. For HIV?
      2. For diabetes?
      3. For hypertension?
      4. For malaria? (*Zambia and Kenya only*)
   2. Would you trust these test results?
2. If you tested negative but still felt sick, what would you do next?
3. If you tested positive on these tests at home, how would you cope with the result?
   1. How would want to be supported when you have a positive result.
   2. If you tested positive and there was a medicine available,
      1. Would you want to talk to someone before getting the medicine?

If yes,

- - - 1. How would you like to talk to them (*in-person, phone, app*)
      2. What would you want to know from them?

1. If you need medication after you had the tests, how would you want to get these meds?
   1. Home delivery, pharmacy, clinic?
   2. What information would you require when receiving the meds?
      - 1. How would you like to talk to them (*in-person, phone, app*)
2. If we wanted to use an App (available for you to download on your cellphone) to store your test results and some of your information, how would you feel about this?
   1. Would you be ok with with the Department/Ministry of Health having access to this information?
   2. Would you be ok with them knowing the location of your neighbourhood? Exact location of your house?
   3. What sort of information would you want to make sure was not linked to your location at all?
      1. What about HIV test results or malaria test results?
   4. How would you want us to protect your information? (Probe for restricted access, anon information etc.)

**Remember that this intervention would provide these tests to you at home, with an app to help you conduct the tests and store your test results.**

1. What would you gain from having an app that helps you test yourself and store your results?
   1. Access to better services, feel supported when tested, etc.
2. What are some of the potential losses from having an app that helps you test yourself and store your results?
   1. Social/relational losses (*trust/privacy/reputation*)
   2. hassle factors (*interference with day to day activities/difficult in using self-tests etc.*),
   3. Comfort (*anxiety, worry about result*)/lack of immediate support
3. What would help you overcome these challenges?
4. What would make this sort of intervention most acceptable to you?
5. Would people like you in your community use this sort of service? Why/why not?
6. Would you be willing to pay for these services?
   1. Why or why not?
   2. How much money would you be willing to spend?
